# Supplementary material for: Induction of an early IFN-γ cellular response and high plasma levels of SDF-1α are inversely associated with COVID-19 severity and residence in rural areas in Kenyan patients
Source: PLoS One. 2025 Sep 11;20(9):e0316967. doi: 10.1371/journal.pone.0316967 (PMC12425234; doi:10.1371/journal.pone.0316967)
Supplement: S1 Table — Each entry represents a distinct peptide along with its associated properties. (DOCX) [file pone.0316967.s001.docx]

**Table S1.**

| **#** | **ID** | **Length** | **N-terminus** | **Sequence** | **C-terminus** | **Hydro** | **MolWt** |
| --- | --- | --- | --- | --- | --- | --- | --- |
| 1 | S_1 | 18 | H- | MFVFLVLLPLVSSQCVNL | OH | 0.89 | 2022.55 |
| 2 | S_2 | 16 | H- | PLVSSQCVNLTTRTQL | OH | 0.37 | 1760.04 |
| 3 | S_3 | 18 | H- | CVNLTTRTQLPPAYTNSF | OH | 0.37 | 2026.3 |
| 4 | S_4 | 16 | H- | QLPPAYTNSFTRGVYY | OH | 0.35 | 1877.09 |
| 5 | S_5 | 16 | H- | TNSFTRGVYYPDKVFR | OH | 0.17 | 1950.19 |
| 6 | S_6 | 18 | H- | GVYYPDKVFRSSVLHSTQ | OH | 0.27 | 2083.34 |
| 7 | S_7 | 17 | H- | FRSSVLHSTQDLFLPFF | OH | 0.56 | 2041.34 |
| 8 | S_8 | 16 | H- | STQDLFLPFFSNVTWF | OH | 0.66 | 1949.2 |
| 9 | S_9 | 15 | H- | LPFFSNVTWFHAIHV | OH | 0.75 | 1815.11 |
| 10 | S_10 | 18 | H- | NVTWFHAIHVSGTNGTKR | OH | 0.23 | 2025.26 |
| 11 | S_11 | 18 | H- | HVSGTNGTKRFDNPVLPF | OH | 0.2 | 1986.22 |
| 12 | S_12 | 16 | H- | KRFDNPVLPFNDGVYF | OH | 0.3 | 1928.18 |
| 13 | S_13 | 18 | H- | VLPFNDGVYFASTEKSNI | OH | 0.32 | 2001.23 |
| 14 | S_14 | 16 | H- | YFASTEKSNIIRGWIF | OH | 0.43 | 1932.21 |
| 15 | S_15 | 17 | H- | KSNIIRGWIFGTTLDSK | OH | 0.29 | 1936.25 |
| 16 | S_16 | 17 | H- | WIFGTTLDSKTQSLLIV | OH | 0.57 | 1922.26 |
| 17 | S_17 | 18 | H- | DSKTQSLLIVNNATNVVI | OH | 0.29 | 1929.2 |
| 18 | S_18 | 17 | H- | IVNNATNVVIKVCEFQF | OH | 0.46 | 1938.28 |
| 19 | S_19 | 18 | H- | VVIKVCEFQFCNDPFLGV | OH | 0.63 | 2057.47 |
| 20 | S_20 | 17 | H- | QFCNDPFLGVYYHKNNK | OH | 0.23 | 2087.35 |
| 21 | S_21 | 18 | H- | LGVYYHKNNKSWMESEFR | OH | 0.15 | 2288.58 |
| 22 | S_22 | 18 | H- | NKSWMESEFRVYSSANNC | OH | 0.12 | 2152.36 |
| 23 | S_23 | 15 | H- | FRVYSSANNCTFEYV | OH | 0.32 | 1799.98 |
| 24 | S_24 | 18 | H- | SANNCTFEYVSQPFLMDL | OH | 0.43 | 2079.34 |
| 25 | S_25 | 18 | H- | YVSQPFLMDLEGKQGNFK | OH | 0.24 | 2101.42 |
| 26 | S_26 | 18 | H- | DLEGKQGNFKNLREFVFK | OH | 0.04 | 2169.47 |
| 27 | S_27 | 18 | H- | FKNLREFVFKNIDGYFKI | OH | 0.31 | 2278.69 |
| 28 | S_28 | 17 | H- | FKNIDGYFKIYSKHTPI | OH | 0.31 | 2071.41 |
| 29 | S_29 | 16 | H- | FKIYSKHTPINLVRDL | OH | 0.33 | 1944.31 |
| 30 | S_30 | 17 | H- | HTPINLVRDLPQGFSAL | OH | 0.4 | 1878.16 |
| 31 | S_31 | 18 | H- | RDLPQGFSALEPLVDLPI | OH | 0.44 | 1980.29 |
| 32 | S_32 | 17 | H- | ALEPLVDLPIGINITRF | OH | 0.56 | 1881.25 |
| 33 | S_33 | 18 | H- | LPIGINITRFQTLLALHR | OH | 0.55 | 2076.52 |
| 34 | S_34 | 18 | H- | RFQTLLALHRSYLTPGDS | OH | 0.32 | 2075.36 |
| 35 | S_35 | 18 | H- | HRSYLTPGDSSSGWTAGA | OH | 0.16 | 1849.94 |
| 36 | S_36 | 18 | H- | DSSSGWTAGAAAYYVGYL | OH | 0.34 | 1838.95 |
| 37 | S_37 | 18 | H- | GAAAYYVGYLQPRTFLLK | OH | 0.45 | 2031.39 |
| 38 | S_38 | 17 | H- | YLQPRTFLLKYNENGTI | OH | 0.32 | 2070.38 |
| 39 | S_39 | 18 | H- | LLKYNENGTITDAVDCAL | OH | 0.28 | 1953.2 |
| 40 | S_40 | 17 | H- | TITDAVDCALDPLSETK | OH | 0.23 | 1791.99 |
| 41 | S_41 | 18 | H- | CALDPLSETKCTLKSFTV | OH | 0.39 | 1956.32 |
| 42 | S_42 | 15 | H- | TKCTLKSFTVEKGIY | OH | 0.26 | 1718.05 |
| 43 | S_43 | 17 | H- | KSFTVEKGIYQTSNFRV | OH | 0.16 | 2004.28 |
| 44 | S_44 | 18 | H- | GIYQTSNFRVQPTESIVR | OH | 0.22 | 2095.35 |
| 45 | S_45 | 17 | H- | RVQPTESIVRFPNITNL | OH | 0.29 | 1984.29 |
| 46 | S_46 | 17 | H- | IVRFPNITNLCPFGEVF | OH | 0.62 | 1966.34 |
| 47 | S_47 | 18 | H- | TNLCPFGEVFNATRFASV | OH | 0.41 | 1973.24 |
| 48 | S_48 | 18 | H- | VFNATRFASVYAWNRKRI | OH | 0.26 | 2199.55 |
| 49 | S_49 | 17 | H- | SVYAWNRKRISNCVADY | OH | 0.2 | 2045.31 |
| 50 | S_50 | 18 | H- | KRISNCVADYSVLYNSAS | OH | 0.21 | 1990.23 |
| 51 | S_51 | 17 | H- | DYSVLYNSASFSTFKCY | OH | 0.38 | 1995.2 |
| 52 | S_52 | 17 | H- | SASFSTFKCYGVSPTKL | OH | 0.35 | 1823.11 |
| 53 | S_53 | 18 | H- | KCYGVSPTKLNDLCFTNV | OH | 0.36 | 2002.35 |
| 54 | S_54 | 18 | H- | KLNDLCFTNVYADSFVIR | OH | 0.39 | 2118.45 |
| 55 | S_55 | 17 | H- | NVYADSFVIRGDEVRQI | OH | 0.18 | 1981.2 |
| 56 | S_56 | 18 | H- | VIRGDEVRQIAPGQTGKI | OH | 0.13 | 1937.23 |
| 57 | S_57 | 17 | H- | QIAPGQTGKIADYNYKL | OH | 0.17 | 1880.13 |
| 58 | S_58 | 18 | H- | GKIADYNYKLPDDFTGCV | OH | 0.23 | 2019.27 |
| 59 | S_59 | 18 | H- | KLPDDFTGCVIAWNSNNL | OH | 0.36 | 2007.26 |
| 60 | S_60 | 18 | H- | CVIAWNSNNLDSKVGGNY | OH | 0.25 | 1954.15 |
| 61 | S_61 | 18 | H- | NLDSKVGGNYNYLYRLFR | OH | 0.18 | 2192.47 |
| 62 | S_62 | 17 | H- | NYNYLYRLFRKSNLKPF | OH | 0.25 | 2236.61 |
| 63 | S_63 | 18 | H- | LFRKSNLKPFERDISTEI | OH | 0.15 | 2193.54 |
| 64 | S_64 | 18 | H- | PFERDISTEIYQAGSTPC | OH | 0.25 | 2014.2 |
| 65 | S_65 | 16 | H- | EIYQAGSTPCNGVEGF | OH | 0.26 | 1671.81 |
| 66 | S_66 | 16 | H- | STPCNGVEGFNCYFPL | OH | 0.48 | 1747.97 |
| 67 | S_67 | 15 | H- | VEGFNCYFPLQSYGF | OH | 0.54 | 1770.99 |
| 68 | S_68 | 18 | H- | CYFPLQSYGFQPTNGVGY | OH | 0.49 | 2041.27 |
| 69 | S_69 | 18 | H- | GFQPTNGVGYQPYRVVVL | OH | 0.41 | 1994.28 |
| 70 | S_70 | 15 | H- | GYQPYRVVVLSFELL | OH | 0.56 | 1783.1 |
| 71 | S_71 | 16 | H- | RVVVLSFELLHAPATV | OH | 0.56 | 1751.1 |
| 72 | S_72 | 15 | H- | FELLHAPATVCGPKK | OH | 0.35 | 1610.94 |
| 73 | S_73 | 18 | H- | APATVCGPKKSTNLVKNK | OH | 0.07 | 1856.22 |
| 74 | S_74 | 16 | H- | KKSTNLVKNKCVNFNF | OH | 0.07 | 1884.24 |
| 75 | S_75 | 18 | H- | VKNKCVNFNFNGLTGTGV | OH | 0.24 | 1912.2 |
| 76 | S_76 | 18 | H- | NFNGLTGTGVLTESNKKF | OH | 0.14 | 1927.15 |
| 77 | S_77 | 18 | H- | GVLTESNKKFLPFQQFGR | OH | 0.23 | 2096.42 |
| 78 | S_78 | 18 | H- | KFLPFQQFGRDIADTTDA | OH | 0.21 | 2070.29 |
| 79 | S_79 | 17 | H- | GRDIADTTDAVRDPQTL | OH | -0.01 | 1843.97 |
| 80 | S_80 | 15 | H- | TDAVRDPQTLEILDI | OH | 0.23 | 1698.89 |
| 81 | S_81 | 18 | H- | DPQTLEILDITPCSFGGV | OH | 0.47 | 1905.16 |
| 82 | S_82 | 18 | H- | DITPCSFGGVSVITPGTN | OH | 0.42 | 1764.98 |
| 83 | S_83 | 18 | H- | GVSVITPGTNTSNQVAVL | OH | 0.35 | 1756.97 |
| 84 | S_84 | 18 | H- | TNTSNQVAVLYQDVNCTE | OH | 0.17 | 1999.14 |
| 85 | S_85 | 15 | H- | VLYQDVNCTEVPVAI | OH | 0.48 | 1662.93 |
| 86 | S_86 | 15 | H- | VNCTEVPVAIHADQL | OH | 0.38 | 1608.84 |
| 87 | S_87 | 17 | H- | VPVAIHADQLTPTWRVY | OH | 0.49 | 1966.27 |
| 88 | S_88 | 17 | H- | DQLTPTWRVYSTGSNVF | OH | 0.33 | 1971.16 |
| 89 | S_89 | 18 | H- | RVYSTGSNVFQTRAGCLI | OH | 0.32 | 1972.26 |
| 90 | S_90 | 15 | H- | VFQTRAGCLIGAEHV | OH | 0.39 | 1600.86 |
| 91 | S_91 | 18 | H- | AGCLIGAEHVNNSYECDI | OH | 0.31 | 1908.1 |
| 92 | S_92 | 16 | H- | HVNNSYECDIPIGAGI | OH | 0.33 | 1701.88 |
| 93 | S_93 | 18 | H- | ECDIPIGAGICASYQTQT | OH | 0.38 | 1870.09 |
| 94 | S_94 | 17 | H- | GICASYQTQTNSPRRAR | OH | 0.01 | 1909.12 |
| 95 | S_95 | 18 | H- | TQTNSPRRARSVASQSII | OH | 0.03 | 1972.19 |
| 96 | S_96 | 16 | H- | ARSVASQSIIAYTMSL | OH | 0.38 | 1697.98 |
| 97 | S_97 | 18 | H- | QSIIAYTMSLGAENSVAY | OH | 0.38 | 1918.16 |
| 98 | S_98 | 17 | H- | SLGAENSVAYSNNSIAI | OH | 0.21 | 1709.83 |
| 99 | S_99 | 18 | H- | VAYSNNSIAIPTNFTISV | OH | 0.44 | 1911.14 |
| 100 | S_100 | 17 | H- | AIPTNFTISVTTEILPV | OH | 0.58 | 1816.13 |
| 101 | S_101 | 17 | H- | ISVTTEILPVSMTKTSV | OH | 0.44 | 1806.15 |
| 102 | S_102 | 16 | H- | LPVSMTKTSVDCTMYI | OH | 0.49 | 1789.17 |
| 103 | S_103 | 18 | H- | KTSVDCTMYICGDSTECS | OH | 0.28 | 1943.19 |
| 104 | S_104 | 16 | H- | YICGDSTECSNLLLQY | OH | 0.45 | 1822.05 |
| 105 | S_105 | 17 | H- | TECSNLLLQYGSFCTQL | OH | 0.51 | 1920.2 |
| 106 | S_106 | 17 | H- | LQYGSFCTQLNRALTGI | OH | 0.43 | 1885.18 |
| 107 | S_107 | 16 | H- | TQLNRALTGIAVEQDK | OH | 0.07 | 1756.98 |
| 108 | S_108 | 16 | H- | LTGIAVEQDKNTQEVF | OH | 0.15 | 1791.98 |
| 109 | S_109 | 18 | H- | EQDKNTQEVFAQVKQIYK | OH | -0.04 | 2196.45 |
| 110 | S_110 | 17 | H- | VFAQVKQIYKTPPIKDF | OH | 0.35 | 2022.42 |
| 111 | S_111 | 18 | H- | IYKTPPIKDFGGFNFSQI | OH | 0.4 | 2072.4 |
| 112 | S_112 | 16 | H- | DFGGFNFSQILPDPSK | OH | 0.28 | 1768.95 |
| 113 | S_113 | 17 | H- | FSQILPDPSKPSKRSFI | OH | 0.27 | 1947.27 |
| 114 | S_114 | 18 | H- | PSKPSKRSFIEDLLFNKV | OH | 0.18 | 2105.47 |
| 115 | S_115 | 18 | H- | FIEDLLFNKVTLADAGFI | OH | 0.53 | 2026.37 |
| 116 | S_116 | 17 | H- | KVTLADAGFIKQYGDCL | OH | 0.32 | 1842.15 |
| 117 | S_117 | 18 | H- | GFIKQYGDCLGDIAARDL | OH | 0.28 | 1955.23 |
| 118 | S_118 | 16 | H- | CLGDIAARDLICAQKF | OH | 0.4 | 1737.08 |
| 119 | S_119 | 16 | H- | ARDLICAQKFNGLTVL | OH | 0.39 | 1762.11 |
| 120 | S_120 | 18 | H- | AQKFNGLTVLPPLLTDEM | OH | 0.42 | 1987.35 |
| 121 | S_121 | 18 | H- | VLPPLLTDEMIAQYTSAL | OH | 0.56 | 1975.34 |
| 122 | S_122 | 15 | H- | EMIAQYTSALLAGTI | OH | 0.47 | 1581.85 |
| 123 | S_123 | 16 | H- | YTSALLAGTITSGWTF | OH | 0.56 | 1688.9 |
| 124 | S_124 | 18 | H- | AGTITSGWTFGAGAALQI | OH | 0.47 | 1721.93 |
| 125 | S_125 | 18 | H- | TFGAGAALQIPFAMQMAY | OH | 0.54 | 1888.24 |
| 126 | S_126 | 17 | H- | QIPFAMQMAYRFNGIGV | OH | 0.49 | 1943.33 |
| 127 | S_127 | 16 | H- | MAYRFNGIGVTQNVLY | OH | 0.4 | 1846.14 |
| 128 | S_128 | 16 | H- | GIGVTQNVLYENQKLI | OH | 0.31 | 1789.06 |
| 129 | S_129 | 18 | H- | NVLYENQKLIANQFNSAI | OH | 0.27 | 2079.34 |
| 130 | S_130 | 17 | H- | LIANQFNSAIGKIQDSL | OH | 0.31 | 1832.09 |
| 131 | S_131 | 17 | H- | SAIGKIQDSLSSTASAL | OH | 0.22 | 1648.83 |
| 132 | S_132 | 17 | H- | DSLSSTASALGKLQDVV | OH | 0.2 | 1690.87 |
| 133 | S_133 | 17 | H- | SALGKLQDVVNQNAQAL | OH | 0.16 | 1768.99 |
| 134 | S_134 | 17 | H- | DVVNQNAQALNTLVKQL | OH | 0.19 | 1868.12 |
| 135 | S_135 | 17 | H- | QALNTLVKQLSSNFGAI | OH | 0.33 | 1804.08 |
| 136 | S_136 | 18 | H- | KQLSSNFGAISSVLNDIL | OH | 0.35 | 1906.17 |
| 137 | S_137 | 16 | H- | AISSVLNDILSRLDKV | OH | 0.3 | 1743.04 |
| 138 | S_138 | 18 | H- | NDILSRLDKVEAEVQIDR | OH | 0.03 | 2113.36 |
| 139 | S_139 | 16 | H- | KVEAEVQIDRLITGRL | OH | 0.17 | 1840.16 |
| 140 | S_140 | 17 | H- | QIDRLITGRLQSLQTYV | OH | 0.32 | 2004.32 |
| 141 | S_141 | 16 | H- | GRLQSLQTYVTQQLIR | OH | 0.27 | 1904.2 |
| 142 | S_142 | 15 | H- | QTYVTQQLIRAAEIR | OH | 0.21 | 1790.05 |
| 143 | S_143 | 15 | H- | QQLIRAAEIRASANL | OH | 0.16 | 1653.9 |
| 144 | S_144 | 15 | H- | AAEIRASANLAATKM | OH | 0.11 | 1517.77 |
| 145 | S_145 | 15 | H- | ASANLAATKMSECVL | OH | 0.28 | 1508.78 |
| 146 | S_146 | 18 | H- | AATKMSECVLGQSKRVDF | OH | 0.16 | 1970.31 |
| 147 | S_147 | 18 | H- | VLGQSKRVDFCGKGYHLM | OH | 0.28 | 2038.43 |
| 148 | S_148 | 18 | H- | DFCGKGYHLMSFPQSAPH | OH | 0.36 | 2022.3 |
| 149 | S_149 | 17 | H- | LMSFPQSAPHGVVFLHV | OH | 0.62 | 1866.22 |
| 150 | S_150 | 18 | H- | APHGVVFLHVTYVPAQEK | OH | 0.41 | 1992.31 |
| 151 | S_151 | 18 | H- | HVTYVPAQEKNFTTAPAI | OH | 0.3 | 1987.24 |
| 152 | S_152 | 18 | H- | EKNFTTAPAICHDGKAHF | OH | 0.18 | 1987.23 |
| 153 | S_153 | 17 | H- | AICHDGKAHFPREGVFV | OH | 0.3 | 1883.17 |
| 154 | S_154 | 18 | H- | AHFPREGVFVSNGTHWFV | OH | 0.44 | 2087.33 |
| 155 | S_155 | 16 | H- | FVSNGTHWFVTQRNFY | OH | 0.41 | 2003.21 |
| 156 | S_156 | 15 | H- | HWFVTQRNFYEPQII | OH | 0.49 | 1978.24 |
| 157 | S_157 | 17 | H- | QRNFYEPQIITTDNTFV | OH | 0.27 | 2086.29 |
| 158 | S_158 | 18 | H- | QIITTDNTFVSGNCDVVI | OH | 0.42 | 1939.18 |
| 159 | S_159 | 18 | H- | FVSGNCDVVIGIVNNTVY | OH | 0.49 | 1913.18 |
| 160 | S_160 | 17 | H- | VIGIVNNTVYDPLQPEL | OH | 0.46 | 1884.16 |
| 161 | S_161 | 17 | H- | TVYDPLQPELDSFKEEL | OH | 0.23 | 2023.23 |
| 162 | S_162 | 15 | H- | PELDSFKEELDKYFK | OH | 0.03 | 1888.11 |
| 163 | S_163 | 17 | H- | FKEELDKYFKNHTSPDV | OH | 0.02 | 2097.32 |
| 164 | S_164 | 18 | H- | YFKNHTSPDVDLGDISGI | OH | 0.23 | 1978.15 |
| 165 | S_165 | 17 | H- | DVDLGDISGINASVVNI | OH | 0.3 | 1700.87 |
| 166 | S_166 | 17 | H- | SGINASVVNIQKEIDRL | OH | 0.17 | 1856.11 |
| 167 | S_167 | 17 | H- | VNIQKEIDRLNEVAKNL | OH | 0.05 | 1996.3 |
| 168 | S_168 | 17 | H- | DRLNEVAKNLNESLIDL | OH | 0.09 | 1956.19 |
| 169 | S_169 | 16 | H- | KNLNESLIDLQELGKY | OH | 0.13 | 1877.13 |
| 170 | S_170 | 18 | H- | LIDLQELGKYEQYIKWPW | OH | 0.48 | 2322.7 |
| 171 | S_171 | 17 | H- | KYEQYIKWPWYIWLGFI | OH | 0.77 | 2333.77 |
| 172 | S_172 | 18 | H- | WPWYIWLGFIAGLIAIVM | OH | 1.11 | 2149.68 |
| 173 | S_173 | 18 | H- | FIAGLIAIVMVTIMLCCM | OH | 1.04 | 1942.62 |
| 174 | S_174 | 18 | H- | VMVTIMLCCMTSCCSCLK | OH | 0.84 | 1969.62 |
| 175 | S_175 | 18 | H- | CMTSCCSCLKGCCSCGSC | OH | 0.63 | 1779.23 |
| 176 | S_176 | 18 | H- | LKGCCSCGSCCKFDEDDS | OH | 0.22 | 1900.15 |
| 177 | S_177 | 18 | H- | SCCKFDEDDSEPVLKGVK | OH | 0.07 | 1999.26 |
| 178 | S_178 | 18 | H- | FDEDDSEPVLKGVKLHYT | OH | 0.12 | 2092.3 |
| 179 | M(ORF5) _1 | 17 | H- | MADSNGTITVEELKKLL | OH | 0.2 | 1862.18 |
| 180 | M(ORF5) _2 | 17 | H- | ITVEELKKLLEQWNLVI | OH | 0.47 | 2068.49 |
| 181 | M(ORF5) _3 | 18 | H- | KLLEQWNLVIGFLFLTWI | OH | 0.87 | 2233.73 |
| 182 | M(ORF5) _4 | 17 | H- | VIGFLFLTWICLLQFAY | OH | 1.07 | 2047.54 |
| 183 | M(ORF5) _5 | 18 | H- | TWICLLQFAYANRNRFLY | OH | 0.59 | 2292.7 |
| 184 | M(ORF5) _6 | 18 | H- | AYANRNRFLYIIKLIFLW | OH | 0.65 | 2314.81 |
| 185 | M(ORF5) _7 | 17 | H- | LYIIKLIFLWLLWPVTL | OH | 1.17 | 2144.77 |
| 186 | M(ORF5) _8 | 18 | H- | FLWLLWPVTLACFVLAAV | OH | 1.1 | 2062.6 |
| 187 | M(ORF5) _9 | 16 | H- | TLACFVLAAVYRINWI | OH | 0.77 | 1853.27 |
| 188 | M(ORF5) _10 | 18 | H- | LAAVYRINWITGGIAIAM | OH | 0.63 | 1933.35 |
| 189 | M(ORF5) _11 | 18 | H- | WITGGIAIAMACLVGLMW | OH | 0.9 | 1906.41 |
| 190 | M(ORF5) _12 | 18 | H- | AMACLVGLMWLSYFIASF | OH | 0.9 | 2023.52 |
| 191 | M(ORF5) _13 | 17 | H- | MWLSYFIASFRLFARTR | OH | 0.58 | 2165.6 |
| 192 | M(ORF5) _14 | 15 | H- | ASFRLFARTRSMWSF | OH | 0.38 | 1863.18 |
| 193 | M(ORF5) _15 | 18 | H- | FARTRSMWSFNPETNILL | OH | 0.41 | 2183.52 |
| 194 | M(ORF5) _16 | 18 | H- | SFNPETNILLNVPLHGTI | OH | 0.48 | 1979.27 |
| 195 | M(ORF5) _17 | 16 | H- | LLNVPLHGTILTRPLL | OH | 0.66 | 1770.19 |
| 196 | M(ORF5) _18 | 16 | H- | HGTILTRPLLESELVI | OH | 0.49 | 1791.12 |
| 197 | M(ORF5) _19 | 16 | H- | RPLLESELVIGAVILR | OH | 0.49 | 1778.17 |
| 198 | M(ORF5) _20 | 15 | H- | ELVIGAVILRGHLRI | OH | 0.53 | 1659.05 |
| 199 | M(ORF5) _21 | 17 | H- | AVILRGHLRIAGHHLGR | OH | 0.32 | 1876.25 |
| 200 | M(ORF5) _22 | 18 | H- | LRIAGHHLGRCDIKDLPK | OH | 0.21 | 2042.44 |
| 201 | M(ORF5) _23 | 18 | H- | GRCDIKDLPKEITVATSR | OH | 0.08 | 2002.32 |
| 202 | M(ORF5) _24 | 17 | H- | PKEITVATSRTLSYYKL | OH | 0.25 | 1970.3 |
| 203 | M(ORF5) _25 | 16 | H- | TSRTLSYYKLGASQRV | OH | 0.13 | 1830.08 |
| 204 | M(ORF5) _26 | 16 | H- | YYKLGASQRVAGDSGF | OH | 0.13 | 1718.89 |
| 205 | M(ORF5) _27 | 18 | H- | SQRVAGDSGFAAYSRYRI | OH | 0.09 | 2004.2 |
| 206 | M(ORF5) _28 | 15 | H- | GFAAYSRYRIGNYKL | OH | 0.2 | 1779.04 |
| 207 | M(ORF5) _29 | 18 | H- | SRYRIGNYKLNTDHSSSS | OH | -0.06 | 2085.23 |
| 208 | M(ORF5) _30 | 17 | H- | KLNTDHSSSSDNIALLV | OH | 0.17 | 1813.99 |
| 209 | M(ORF5) _31 | 18 | H- | KLNTDHSSSSDNIALLVQ | OH | 0.15 | 1942.12 |
| 210 | N(ORF9) _1 | 17 | H- | MSDNGPQNQRNAPRITF | OH | 0 | 1946.13 |
| 211 | N(ORF9) _2 | 18 | H- | NQRNAPRITFGGPSDSTG | OH | -0.01 | 1874.99 |
| 212 | N(ORF9) _3 | 17 | H- | TFGGPSDSTGSNQNGER | OH | -0.14 | 1710.69 |
| 213 | N(ORF9) _4 | 18 | H- | STGSNQNGERSGARSKQR | OH | -0.38 | 1919.99 |
| 214 | N(ORF9) _5 | 15 | H- | ERSGARSKQRRPQGL | OH | -0.31 | 1725.93 |
| 215 | N(ORF9) _6 | 18 | H- | RSKQRRPQGLPNNTASWF | OH | 0.01 | 2143.4 |
| 216 | N(ORF9) _7 | 18 | H- | GLPNNTASWFTALTQHGK | OH | 0.27 | 1943.15 |
| 217 | N(ORF9) _8 | 17 | H- | WFTALTQHGKEDLKFPR | OH | 0.24 | 2074.38 |
| 218 | N(ORF9) _9 | 16 | H- | HGKEDLKFPRGQGVPI | OH | 0.09 | 1778.05 |
| 219 | N(ORF9) _10 | 18 | H- | KFPRGQGVPINTNSSPDD | OH | 0.02 | 1929.08 |
| 220 | N(ORF9) _11 | 17 | H- | PINTNSSPDDQIGYYRR | OH | 0.02 | 1996.13 |
| 221 | N(ORF9) _12 | 16 | H- | PDDQIGYYRRATRRIR | OH | -0.1 | 2036.29 |
| 222 | N(ORF9) _13 | 17 | H- | YYRRATRRIRGGDGKMK | OH | -0.21 | 2084.44 |
| 223 | N(ORF9) _14 | 18 | H- | RIRGGDGKMKDLSPRWYF | OH | 0.1 | 2182.54 |
| 224 | N(ORF9) _15 | 18 | H- | MKDLSPRWYFYYLGTGPE | OH | 0.4 | 2223.54 |
| 225 | N(ORF9) _16 | 15 | H- | YFYYLGTGPEAGLPY | OH | 0.51 | 1710.91 |
| 226 | N(ORF9) _17 | 18 | H- | GTGPEAGLPYGANKDGII | OH | 0.19 | 1729.91 |
| 227 | N(ORF9) _18 | 18 | H- | PYGANKDGIIWVATEGAL | OH | 0.34 | 1875.11 |
| 228 | N(ORF9) _19 | 17 | H- | IIWVATEGALNTPKDHI | OH | 0.41 | 1878.16 |
| 229 | N(ORF9) _20 | 18 | H- | GALNTPKDHIGTRNPANN | OH | -0.04 | 1890.05 |
| 230 | N(ORF9)_21 | 17 | H- | HIGTRNPANNAAIVLQL | OH | 0.29 | 1802.07 |
| 231 | N(ORF9) _22 | 18 | H- | ANNAAIVLQLPQGTTLPK | OH | 0.33 | 1849.16 |
| 232 | N(ORF9) _23 | 18 | H- | QLPQGTTLPKGFYAEGSR | OH | 0.18 | 1950.18 |
| 233 | N(ORF9) _24 | 18 | H- | PKGFYAEGSRGGSQASSR | OH | -0.09 | 1841.96 |
| 234 | N(ORF9) _25 | 16 | H- | SRGGSQASSRSSSRSR | OH | -0.34 | 1652.71 |
| 235 | N(ORF9) _26 | 18 | H- | ASSRSSSRSRNSSRNSTP | OH | -0.3 | 1923.98 |
| 236 | N(ORF9) _27 | 18 | H- | SRNSSRNSTPGSSRGTSP | OH | -0.21 | 1834.88 |
| 237 | N(ORF9) _28 | 18 | H- | TPGSSRGTSPARMAGNGG | OH | -0.03 | 1660.79 |
| 238 | N(ORF9) _29 | 18 | H- | SPARMAGNGGDAALALLL | OH | 0.31 | 1697.98 |
| 239 | N(ORF9) _30 | 17 | H- | GGDAALALLLLDRLNQL | OH | 0.41 | 1766.07 |
| 240 | N(ORF9) _31 | 17 | H- | LLLLDRLNQLESKMSGK | OH | 0.23 | 1958.36 |
| 241 | N(ORF9) _32 | 18 | H- | NQLESKMSGKGQQQQGQT | OH | -0.16 | 1977.14 |
| 242 | N(ORF9) _33 | 18 | H- | GKGQQQQGQTVTKKSAAE | OH | -0.21 | 1874.04 |
| 243 | N(ORF9) _34 | 18 | H- | QTVTKKSAAEASKKPRQK | OH | -0.27 | 1986.31 |
| 244 | N(ORF9) _35 | 17 | H- | AEASKKPRQKRTATKAY | OH | -0.27 | 1934.23 |
| 245 | N(ORF9) _36 | 18 | H- | RQKRTATKAYNVTQAFGR | OH | -0.11 | 2096.38 |
| 246 | N(ORF9) _37 | 18 | H- | AYNVTQAFGRRGPEQTQG | OH | 0.01 | 1980.13 |
| 247 | N(ORF9) _38 | 18 | H- | GRRGPEQTQGNFGDQELI | OH | -0.04 | 2002.13 |
| 248 | N(ORF9) _39 | 17 | H- | QGNFGDQELIRQGTDYK | OH | -0.04 | 1969.1 |
| 249 | N(ORF9) _40 | 18 | H- | ELIRQGTDYKHWPQIAQF | OH | 0.29 | 2230.52 |
| 250 | N(ORF9) _41 | 18 | H- | YKHWPQIAQFAPSASAFF | OH | 0.5 | 2096.38 |
| 251 | N(ORF9) _42 | 17 | H- | QFAPSASAFFGMSRIGM | OH | 0.43 | 1805.12 |
| 252 | N(ORF9) _43 | 18 | H- | AFFGMSRIGMEVTPSGTW | OH | 0.47 | 1974.3 |
| 253 | N(ORF9) _44 | 18 | H- | GMEVTPSGTWLTYTGAIK | OH | 0.39 | 1912.2 |
| 254 | N(ORF9) _45 | 18 | H- | TWLTYTGAIKLDDKDPNF | OH | 0.27 | 2098.34 |
| 255 | N(ORF9) _46 | 17 | H- | IKLDDKDPNFKDQVILL | OH | 0.2 | 2014.35 |
| 256 | N(ORF9) _47 | 18 | H- | PNFKDQVILLNKHIDAYK | OH | 0.22 | 2156.52 |
| 257 | N(ORF9) _48 | 18 | H- | LLNKHIDAYKTFPPTEPK | OH | 0.22 | 2112.46 |
| 258 | N(ORF9) _49 | 16 | H- | YKTFPPTEPKKDKKKK | OH | -0.26 | 1963.36 |
| 259 | N(ORF9) _50 | 17 | H- | TEPKKDKKKKADETQAL | OH | -0.38 | 1958.25 |
| 260 | N(ORF9) _51 | 16 | H- | KKKADETQALPQRQKK | OH | -0.36 | 1897.21 |
| 261 | N(ORF9) _52 | 17 | H- | TQALPQRQKKQQTVTLL | OH | 0.12 | 1981.33 |
| 262 | N(ORF9) _53 | 18 | H- | QKKQQTVTLLPAADLDDF | OH | 0.16 | 2031.3 |
| 263 | N(ORF9) _54 | 18 | H- | LLPAADLDDFSKQLQQSM | OH | 0.27 | 2020.29 |
| 264 | N(ORF9) _55 | 18 | H- | DFSKQLQQSMSSADSTQA | OH | 0.01 | 1959.08 |
| 265 | ORF1a/1ab_207 | 18 | H- | DWSYSGQSTQLGIEFLKR | OH | 0.24 | 2115.34 |
| 266 | ORF1a/1ab_208 | 17 | H- | TQLGIEFLKRGDKSVYY | OH | 0.21 | 2017.32 |
| 267 | ORF1a/1ab_209 | 17 | H- | LKRGDKSVYYTSNPTTF | OH | 0.1 | 1977.21 |
| 268 | ORF1a/1ab_210 | 17 | H- | VYYTSNPTTFHLDGEVI | OH | 0.39 | 1956.14 |
| 269 | ORF1a/1ab_211 | 18 | H- | TTFHLDGEVITFDNLKTL | OH | 0.37 | 2064.33 |
| 270 | ORF1a/1ab_212 | 17 | H- | VITFDNLKTLLSLREVR | OH | 0.34 | 2017.4 |
| 271 | ORF1a/1ab_213 | 18 | H- | KTLLSLREVRTIKVFTTV | OH | 0.35 | 2104.57 |
| 272 | ORF1a/1ab_214 | 15 | H- | VRTIKVFTTVDNINL | OH | 0.34 | 1733.04 |
| 273 | ORF1a/1ab_215 | 17 | H- | VFTTVDNINLHTQVVDM | OH | 0.4 | 1946.21 |
| 274 | ORF1a/1ab_216 | 18 | H- | INLHTQVVDMSMTYGQQF | OH | 0.4 | 2112.42 |
| 275 | ORF1a/1ab_217 | 15 | H- | DMSMTYGQQFGPTYL | OH | 0.36 | 1738.96 |
| 276 | ORF1a/1ab_218 | 18 | H- | YGQQFGPTYLDGADVTKI | OH | 0.26 | 1973.17 |
| 277 | ORF1a/1ab_219 | 18 | H- | YLDGADVTKIKPHNSHEG | OH | 0.03 | 1981.16 |
| 278 | ORF1a/1ab_220 | 16 | H- | KIKPHNSHEGKTFYVL | OH | 0.15 | 1898.2 |
| 279 | ORF1a/1ab_221 | 18 | H- | SHEGKTFYVLPNDDTLRV | OH | 0.17 | 2091.31 |
| 280 | ORF1a/1ab_222 | 16 | H- | VLPNDDTLRVEAFEYY | OH | 0.26 | 1944.13 |
| 281 | ORF1a/1ab_223 | 18 | H- | TLRVEAFEYYHTTDPSFL | OH | 0.36 | 2189.41 |
| 282 | ORF1a/1ab_224 | 17 | H- | YYHTTDPSFLGRYMSAL | OH | 0.39 | 2022.27 |
| 283 | ORF1a/1ab_225 | 18 | H- | SFLGRYMSALNHTKKWKY | OH | 0.24 | 2230.63 |
| 284 | ORF1a/1ab_226 | 16 | H- | ALNHTKKWKYPQVNGL | OH | 0.17 | 1897.21 |
| 285 | ORF1a/1ab_227 | 15 | H- | KWKYPQVNGLTSIKW | OH | 0.33 | 1848.18 |
| 286 | ORF1a/1ab_228 | 17 | H- | QVNGLTSIKWADNNCYL | OH | 0.32 | 1939.18 |
| 287 | ORF1a/1ab_229 | 17 | H- | IKWADNNCYLATALLTL | OH | 0.53 | 1923.27 |
| 288 | ORF1a/1ab_230 | 17 | H- | CYLATALLTLQQIELKF | OH | 0.63 | 1968.39 |
| 289 | ORF1a/1ab_231 | 15 | H- | LTLQQIELKFNPPAL | OH | 0.47 | 1725.06 |
| 290 | ORF1a/1ab_232 | 18 | H- | IELKFNPPALQDAYYRAR | OH | 0.21 | 2165.48 |
| 291 | ORF1a/1ab_233 | 17 | H- | ALQDAYYRARAGEAANF | OH | 0.07 | 1887.04 |
| 292 | ORF1a/1ab_234 | 17 | H- | RARAGEAANFCALILAY | OH | 0.33 | 1810.11 |
| 293 | ORF1a/1ab_235 | 18 | H- | ANFCALILAYCNKTVGEL | OH | 0.53 | 1943.32 |
| 294 | ORF1a/1ab_236 | 17 | H- | AYCNKTVGELGDVRETM | OH | 0.12 | 1886.14 |
| 295 | ORF1a/1ab_237 | 18 | H- | GELGDVRETMSYLFQHAN | OH | 0.18 | 2067.27 |
| 296 | ORF1a/1ab_238 | 18 | H- | TMSYLFQHANLDSCKRVL | OH | 0.35 | 2126.49 |
| 297 | ORF1a/1ab_239 | 15 | H- | ANLDSCKRVLNVVCK | OH | 0.22 | 1662.01 |
| 298 | ORF1a/1ab_240 | 18 | H- | CKRVLNVVCKTCGQQQTT | OH | 0.24 | 2009.41 |
| 299 | ORF1a/1ab_241 | 18 | H- | CKTCGQQQTTLKGVEAVM | OH | 0.23 | 1925.28 |
| 300 | ORF1a/1ab_242 | 17 | H- | TTLKGVEAVMYMGTLSY | OH | 0.41 | 1864.22 |
| 301 | ORF1a/1ab_243 | 17 | H- | AVMYMGTLSYEQFKKGV | OH | 0.33 | 1952.33 |
| 302 | ORF1a/1ab_244 | 18 | H- | LSYEQFKKGVQIPCTCGK | OH | 0.28 | 2029.42 |
| 303 | ORF1a/1ab_245 | 17 | H- | GVQIPCTCGKQATKYLV | OH | 0.39 | 1809.19 |
| 304 | ORF1a/1ab_246 | 18 | H- | CGKQATKYLVQQESPFVM | OH | 0.29 | 2057.43 |
| 305 | ORF1a/1ab_247 | 18 | H- | LVQQESPFVMMSAPPAQY | OH | 0.45 | 2023.36 |
| 306 | ORF1a/1ab_248 | 17 | H- | VMMSAPPAQYELKHGTF | OH | 0.36 | 1907.25 |
| 307 | ORF1a/1ab_249 | 16 | H- | AQYELKHGTFTCASEY | OH | 0.22 | 1848.03 |
| 308 | ORF1a/1ab_250 | 18 | H- | HGTFTCASEYTGNYQCGH | OH | 0.24 | 1976.1 |
| 309 | ORF1a/1ab_251 | 17 | H- | EYTGNYQCGHYKHITSK | OH | 0.09 | 2029.22 |
| 310 | ORF1a/1ab_252 | 16 | H- | CGHYKHITSKETLYCI | OH | 0.37 | 1896.23 |
| 311 | ORF1a/1ab_253 | 17 | H- | ITSKETLYCIDGALLTK | OH | 0.37 | 1869.21 |
| 312 | ORF1a/1ab_254 | 18 | H- | YCIDGALLTKSSEYKGPI | OH | 0.33 | 1958.27 |
| 313 | ORF1a/1ab_255 | 16 | H- | TKSSEYKGPITDVFYK | OH | 0.1 | 1863.1 |
| 314 | ORF1a/1ab_256 | 18 | H- | KGPITDVFYKENSYTTTI | OH | 0.23 | 2077.32 |
| 315 | ORF1a/1ab_257 | 17 | H- | YKENSYTTTIKPVTYKL | OH | 0.18 | 2049.36 |
| 316 | ORF1a/1ab_258 | 18 | H- | TTIKPVTYKLDGVVCTEI | OH | 0.4 | 1980.36 |
| 317 | ORF1a/1ab_259 | 18 | H- | KLDGVVCTEIDPKLDNYY | OH | 0.24 | 2085.37 |
| 318 | ORF1a/1ab_260 | 17 | H- | EIDPKLDNYYKKDNSYF | OH | 0 | 2152.35 |
| 319 | ORF1a/1ab_261 | 18 | H- | NYYKKDNSYFTEQPIDLV | OH | 0.15 | 2237.46 |
| 320 | ORF1a/1ab_262 | 15 | H- | YFTEQPIDLVPNQPY | OH | 0.39 | 1824.02 |
| 321 | ORF1a/1ab_263 | 18 | H- | PIDLVPNQPYPNASFDNF | OH | 0.35 | 2048.24 |
| 322 | ORF1a/1ab_264 | 18 | H- | PYPNASFDNFKFVCDNIK | OH | 0.28 | 2119.39 |
| 323 | ORF1a/1ab_265 | 18 | H- | NFKFVCDNIKFADDLNQL | OH | 0.28 | 2144.44 |
| 324 | ORF1a/1ab_266 | 15 | H- | IKFADDLNQLTGYKK | OH | 0.08 | 1754.02 |
| 325 | ORF1a/1ab_267 | 18 | H- | DLNQLTGYKKPASRELKV | OH | 0.03 | 2060.39 |
| 326 | ORF1a/1ab_268 | 16 | H- | KKPASRELKVTFFPDL | OH | 0.17 | 1876.23 |
| 327 | ORF1a/1ab_269 | 17 | H- | ELKVTFFPDLNGDVVAI | OH | 0.43 | 1877.17 |
| 328 | ORF1a/1ab_270 | 15 | H- | PDLNGDVVAIDYKHY | OH | 0.2 | 1718.89 |
| 329 | ORF1a/1ab_271 | 16 | H- | DVVAIDYKHYTPSFKK | OH | 0.17 | 1911.19 |
| 330 | ORF1a/1ab_272 | 17 | H- | YKHYTPSFKKGAKLLHK | OH | 0.1 | 2046.45 |
| 331 | ORF1a/1ab_273 | 16 | H- | FKKGAKLLHKPIVWHV | OH | 0.4 | 1901.38 |
| 332 | ORF1a/1ab_274 | 16 | H- | LLHKPIVWHVNNATNK | OH | 0.33 | 1884.22 |
| 333 | ORF1a/1ab_275 | 18 | H- | VWHVNNATNKATYKPNTW | OH | 0.2 | 2144.38 |
| 334 | ORF1a/1ab_276 | 16 | H- | NKATYKPNTWCIRCLW | OH | 0.42 | 1997.38 |
| 335 | ORF1a/1ab_277 | 15 | H- | PNTWCIRCLWSTKPV | OH | 0.58 | 1804.17 |
| 336 | ORF1a/1ab_278 | 18 | H- | IRCLWSTKPVETSNSFDV | OH | 0.34 | 2082.37 |
| 337 | ORF1a/1ab_279 | 18 | H- | PVETSNSFDVLKSEDAQG | OH | 0.04 | 1923.02 |
| 338 | ORF1a/1ab_280 | 18 | H- | DVLKSEDAQGMDNLACED | OH | 0 | 1953.09 |
| 339 | ORF1a/1ab_281 | 18 | H- | QGMDNLACEDLKPVSEEV | OH | 0.13 | 1977.2 |
| 340 | ORF1a/1ab_282 | 18 | H- | EDLKPVSEEVVENPTIQK | OH | 0.05 | 2054.29 |
| 341 | ORF1a/1ab_283 | 18 | H- | EVVENPTIQKDVLECNVK | OH | 0.15 | 2057.36 |
| 342 | ORF1a/1ab_284 | 18 | H- | QKDVLECNVKTTEVVGDI | OH | 0.15 | 1990.26 |
| 343 | ORF1a/1ab_285 | 18 | H- | VKTTEVVGDIILKPANNS | OH | 0.21 | 1898.19 |
| 344 | ORF1a/1ab_286 | 17 | H- | DIILKPANNSLKITEEV | OH | 0.23 | 1897.2 |
| 345 | ORF1a/1ab_287 | 16 | H- | NNSLKITEEVGHTDLM | OH | 0.13 | 1801.01 |
| 346 | ORF1a/1ab_288 | 18 | H- | TEEVGHTDLMAAYVDNSS | OH | 0.12 | 1939.05 |
| 347 | ORF1a/1ab_289 | 15 | H- | LMAAYVDNSSLTIKK | OH | 0.25 | 1653.96 |
| 348 | ORF1a/1ab_290 | 18 | H- | VDNSSLTIKKPNELSRVL | OH | 0.14 | 2013.33 |
| 349 | ORF1a/1ab_291 | 15 | H- | KKPNELSRVLGLKTL | OH | 0.12 | 1696.07 |
| 350 | ORF1a/1ab_292 | 18 | H- | LSRVLGLKTLATHGLAAV | OH | 0.44 | 1820.21 |
| 351 | ORF1a/1ab_293 | 18 | H- | TLATHGLAAVNSVPWDTI | OH | 0.46 | 1866.11 |
| 352 | ORF1a/1ab_294 | 18 | H- | AVNSVPWDTIANYAKPFL | OH | 0.44 | 2006.29 |
| 353 | ORF1a/1ab_295 | 18 | H- | TIANYAKPFLNKVVSTTT | OH | 0.3 | 1968.28 |
| 354 | ORF1a/1ab_296 | 17 | H- | FLNKVVSTTTNIVTRCL | OH | 0.43 | 1909.28 |
| 355 | ORF1a/1ab_297 | 18 | H- | TTTNIVTRCLNRVCTNYM | OH | 0.35 | 2103.48 |
| 356 | ORF1a/1ab_298 | 18 | H- | CLNRVCTNYMPYFFTLLL | OH | 0.76 | 2211.71 |
| 357 | ORF1a/1ab_299 | 17 | H- | YMPYFFTLLLQLCTFTR | OH | 0.81 | 2157.63 |
| 358 | ORF1a/1ab_300 | 17 | H- | LLLQLCTFTRSTNSRIK | OH | 0.37 | 1994.39 |
| 359 | ORF1a/1ab_301 | 17 | H- | FTRSTNSRIKASMPTTI | OH | 0.17 | 1911.21 |
| 360 | ORF1a/1ab_302 | 18 | H- | RIKASMPTTIAKNTVKSV | OH | 0.15 | 1945.36 |
| 361 | ORF1a/1ab_303 | 15 | H- | TIAKNTVKSVGKFCL | OH | 0.28 | 1608.97 |
| 362 | ORF1a/1ab_304 | 18 | H- | TVKSVGKFCLEASFNYLK | OH | 0.32 | 2034.41 |
| 363 | ORF1a/1ab_305 | 18 | H- | CLEASFNYLKSPNFSKLI | OH | 0.41 | 2074.44 |
| 364 | ORF1a/1ab_306 | 18 | H- | LKSPNFSKLINIIIWFLL | OH | 0.78 | 2159.69 |
| 365 | ORF1a/1ab_307 | 18 | H- | LINIIIWFLLLSVCLGSL | OH | 1.09 | 2030.6 |
| 366 | ORF1a/1ab_308 | 17 | H- | LLLSVCLGSLIYSTAAL | OH | 0.77 | 1737.14 |
| 367 | ORF1a/1ab_309 | 17 | H- | GSLIYSTAALGVLMSNL | OH | 0.55 | 1710.03 |
| 368 | ORF1a/1ab_310 | 15 | H- | AALGVLMSNLGMPSY | OH | 0.51 | 1523.84 |
| 369 | ORF1a/1ab_311 | 18 | H- | LMSNLGMPSYCTGYREGY | OH | 0.36 | 2042.35 |
| 370 | ORF1a/1ab_312 | 18 | H- | SYCTGYREGYLNSTNVTI | OH | 0.26 | 2041.23 |
| 371 | ORF1a/1ab_313 | 18 | H- | GYLNSTNVTIATYCTGSI | OH | 0.41 | 1878.09 |
| 372 | ORF1a/1ab_314 | 16 | H- | TIATYCTGSIPCSVCL | OH | 0.67 | 1631.96 |
| 373 | ORF1a/1ab_315 | 16 | H- | TGSIPCSVCLSGLDSL | OH | 0.53 | 1551.8 |
| 374 | ORF1a/1ab_316 | 16 | H- | SVCLSGLDSLDTYPSL | OH | 0.45 | 1669.87 |
| 375 | ORF1a/1ab_317 | 17 | H- | LDSLDTYPSLETIQITI | OH | 0.46 | 1922.16 |
| 376 | ORF1a/1ab_318 | 17 | H- | PSLETIQITISSFKWDL | OH | 0.5 | 1978.28 |
| 377 | ORF1a/1ab_319 | 16 | H- | ITISSFKWDLTAFGLV | OH | 0.63 | 1798.12 |
| 378 | ORF1a/1ab_320 | 18 | H- | KWDLTAFGLVAEWFLAYI | OH | 0.71 | 2143.52 |
| 379 | ORF1a/1ab_321 | 18 | H- | LVAEWFLAYILFTRFFYV | OH | 0.91 | 2298.76 |
| 380 | ORF1a/1ab_322 | 17 | H- | YILFTRFFYVLGLAAIM | OH | 0.87 | 2038.53 |
| 381 | ORF1a/1ab_323 | 17 | H- | FYVLGLAAIMQLFFSYF | OH | 0.9 | 2030.47 |
| 382 | ORF1a/1ab_324 | 15 | H- | AIMQLFFSYFAVHFI | OH | 0.86 | 1834.22 |
| 383 | ORF1a/1ab_325 | 18 | H- | FFSYFAVHFISNSWLMWL | OH | 0.91 | 2295.7 |
| 384 | ORF1a/1ab_326 | 17 | H- | FISNSWLMWLIINLVQM | OH | 0.91 | 2108.61 |
| 385 | ORF1a/1ab_327 | 18 | H- | MWLIINLVQMAPISAMVR | OH | 0.74 | 2086.67 |
| 386 | ORF1a/1ab_328 | 18 | H- | QMAPISAMVRMYIFFASF | OH | 0.67 | 2110.6 |
| 387 | ORF1a/1ab_329 | 18 | H- | VRMYIFFASFYYVWKSYV | OH | 0.73 | 2369.83 |
| 388 | ORF1a/1ab_330 | 18 | H- | SFYYVWKSYVHVVDGCNS | OH | 0.46 | 2153.41 |
| 389 | ORF1a/1ab_331 | 18 | H- | YVHVVDGCNSSTCMMCYK | OH | 0.44 | 2040.42 |
| 390 | ORF1a/1ab_332 | 17 | H- | NSSTCMMCYKRNRATRV | OH | 0.08 | 2021.4 |
| 391 | ORF1a/1ab_333 | 16 | H- | CYKRNRATRVECTTIV | OH | 0.13 | 1913.26 |
| 392 | ORF1a/1ab_334 | 18 | H- | ATRVECTTIVNGVRRSFY | OH | 0.24 | 2072.38 |
| 393 | ORF1a/1ab_335 | 17 | H- | IVNGVRRSFYVYANGGK | OH | 0.18 | 1900.17 |
| 394 | ORF1a/1ab_336 | 18 | H- | SFYVYANGGKGFCKLHNW | OH | 0.38 | 2091.39 |
| 395 | ORF1a/1ab_337 | 18 | H- | GKGFCKLHNWNCVNCDTF | OH | 0.37 | 2086.41 |
| 396 | ORF1a/1ab_338 | 17 | H- | NWNCVNCDTFCAGSTFI | OH | 0.52 | 1895.13 |
| 397 | ORF1a/1ab_339 | 18 | H- | DTFCAGSTFISDEVARDL | OH | 0.26 | 1947.11 |
| 398 | ORF1a/1ab_340 | 18 | H- | FISDEVARDLSLQFKRPI | OH | 0.28 | 2134.47 |
| 399 | ORF1a/1ab_341 | 18 | H- | DLSLQFKRPINPTDQSSY | OH | 0.16 | 2109.33 |
| 400 | ORF1a/1ab_342 | 18 | H- | PINPTDQSSYIVDSVTVK | OH | 0.25 | 1963.18 |
| 401 | ORF1a/1ab_343 | 18 | H- | SYIVDSVTVKNGSIHLYF | OH | 0.44 | 2042.33 |
| 402 | ORF1a/1ab_344 | 18 | H- | VKNGSIHLYFDKAGQKTY | OH | 0.14 | 2069.35 |
| 403 | ORF1a/1ab_345 | 18 | H- | YFDKAGQKTYERHSLSHF | OH | 0.08 | 2214.43 |
| 404 | ORF1a/1ab_346 | 17 | H- | TYERHSLSHFVNLDNLR | OH | 0.16 | 2101.31 |
| 405 | ORF1a/1ab_347 | 18 | H- | SHFVNLDNLRANNTKGSL | OH | 0.09 | 2000.21 |
| 406 | ORF1a/1ab_348 | 17 | H- | LRANNTKGSLPINVIVF | OH | 0.36 | 1856.2 |
| 407 | ORF1a/1ab_349 | 15 | H- | GSLPINVIVFDGKSK | OH | 0.3 | 1573.86 |
| 408 | ORF1a/1ab_350 | 17 | H- | NVIVFDGKSKCEESSAK | OH | 0.02 | 1841.08 |
| 409 | ORF1a/1ab_351 | 16 | H- | KSKCEESSAKSASVYY | OH | -0.05 | 1766.95 |
| 410 | ORF1a/1ab_352 | 18 | H- | SSAKSASVYYSQLMCQPI | OH | 0.37 | 1963.27 |
| 411 | ORF1a/1ab_353 | 18 | H- | YYSQLMCQPILLLDQALV | OH | 0.7 | 2111.56 |
| 412 | ORF1a/1ab_354 | 18 | H- | PILLLDQALVSDVGDSAE | OH | 0.37 | 1855.08 |
| 413 | ORF1a/1ab_355 | 16 | H- | LVSDVGDSAEVAVKMF | OH | 0.29 | 1666.92 |
| 414 | ORF1a/1ab_356 | 17 | H- | DSAEVAVKMFDAYVNTF | OH | 0.27 | 1907.13 |
| 415 | ORF1a/1ab_357 | 18 | H- | KMFDAYVNTFSSTFNVPM | OH | 0.4 | 2099.42 |
| 416 | ORF1a/1ab_358 | 17 | H- | TFSSTFNVPMEKLKTLV | OH | 0.37 | 1942.31 |
| 417 | ORF1a/1ab_359 | 17 | H- | VPMEKLKTLVATAEAEL | OH | 0.28 | 1843.22 |
| 418 | ORF1a/1ab_360 | 16 | H- | TLVATAEAELAKNVSL | OH | 0.26 | 1629.87 |
| 419 | ORF1a/1ab_361 | 18 | H- | EAELAKNVSLDNVLSTFI | OH | 0.3 | 1963.22 |
| 420 | ORF1a/1ab_362 | 18 | H- | SLDNVLSTFISAARQGFV | OH | 0.38 | 1925.18 |
| 421 | ORF1a/1ab_363 | 17 | H- | FISAARQGFVDSDVETK | OH | 0.13 | 1870.05 |
| 422 | ORF1a/1ab_364 | 18 | H- | GFVDSDVETKDVVECLKL | OH | 0.23 | 1996.27 |
| 423 | ORF1a/1ab_365 | 18 | H- | TKDVVECLKLSHQSDIEV | OH | 0.2 | 2043.33 |
| 424 | ORF1a/1ab_366 | 18 | H- | KLSHQSDIEVTGDSCNNY | OH | 0.06 | 2010.13 |
| 425 | ORF1a/1ab_367 | 17 | H- | EVTGDSCNNYMLTYNKV | OH | 0.18 | 1951.17 |
| 426 | ORF1a/1ab_368 | 18 | H- | NNYMLTYNKVENMTPRDL | OH | 0.13 | 2216.53 |
| 427 | ORF1a/1ab_369 | 18 | H- | KVENMTPRDLGACIDCSA | OH | 0.2 | 1923.22 |
| 428 | ORF1a/1ab_370 | 17 | H- | DLGACIDCSARHINAQV | OH | 0.29 | 1786.02 |
| 429 | ORF1a/1ab_371 | 18 | H- | CSARHINAQVAKSHNIAL | OH | 0.21 | 1933.23 |
| 430 | ORF1a/1ab_372 | 18 | H- | QVAKSHNIALIWNVKDFM | OH | 0.39 | 2114.51 |
| 431 | ORF1a/1ab_373 | 18 | H- | ALIWNVKDFMSLSEQLRK | OH | 0.33 | 2178.59 |
| 432 | ORF1a/1ab_374 | 18 | H- | FMSLSEQLRKQIRSAAKK | OH | 0.04 | 2121.54 |
| 433 | ORF1a/1ab_375 | 17 | H- | RKQIRSAAKKNNLPFKL | OH | -0.04 | 2012.44 |
| 434 | ORF1a/1ab_376 | 18 | H- | AKKNNLPFKLTCATTRQV | OH | 0.14 | 2033.43 |
| 435 | ORF1a/1ab_377 | 18 | H- | KLTCATTRQVVNVVTTKI | OH | 0.3 | 1975.38 |
| 436 | ORF1a/1ab_378 | 18 | H- | QVVNVVTTKIALKGGKIV | OH | 0.33 | 1867.31 |
| 437 | ORF1a/1ab_379 | 18 | H- | KIALKGGKIVNNWLKQLI | OH | 0.36 | 2036.54 |
| 438 | ORF1ab-1FS_665 | 15 | H- | EVVDKYFDCYDGGCI | OH | 0.31 | 1725.92 |
| 439 | ORF1ab-1FS_666 | 17 | H- | YFDCYDGGCINANQVIV | OH | 0.44 | 1894.12 |
| 440 | ORF1ab-1FS_667 | 15 | H- | GCINANQVIVNNLDK | OH | 0.2 | 1614.84 |
| 441 | ORF1ab-1FS_668 | 18 | H- | NQVIVNNLDKSAGFPFNK | OH | 0.17 | 2005.26 |
| 442 | ORF1ab-1FS_669 | 18 | H- | DKSAGFPFNKWGKARLYY | OH | 0.18 | 2148.46 |
| 443 | ORF1ab-1FS_670 | 15 | H- | NKWGKARLYYDSMSY | OH | 0.13 | 1882.13 |
| 444 | ORF1ab-1FS_671 | 17 | H- | ARLYYDSMSYEDQDALF | OH | 0.2 | 2087.26 |
| 445 | ORF1ab-1FS_672 | 18 | H- | MSYEDQDALFAYTKRNVI | OH | 0.19 | 2164.43 |
| 446 | ORF1ab-1FS_673 | 18 | H- | LFAYTKRNVIPTITQMNL | OH | 0.44 | 2123.55 |
| 447 | ORF1ab-1FS_674 | 17 | H- | VIPTITQMNLKYAISAK | OH | 0.41 | 1891.31 |
| 448 | ORF1ab-1FS_675 | 16 | H- | MNLKYAISAKNRARTV | OH | 0.06 | 1836.19 |
| 449 | ORF1ab-1FS_676 | 15 | H- | ISAKNRARTVAGVSI | OH | 0.1 | 1542.8 |
| 450 | ORF1ab-1FS_677 | 17 | H- | RARTVAGVSICSTMTNR | OH | 0.15 | 1823.13 |
| 451 | ORF1ab-1FS_678 | 18 | H- | VSICSTMTNRQFHQKLLK | OH | 0.29 | 2134.56 |
| 452 | ORF1ab-1FS_679 | 16 | H- | NRQFHQKLLKSIAATR | OH | 0.06 | 1911.24 |
| 453 | ORF1ab-1FS_680 | 16 | H- | KLLKSIAATRGATVVI | OH | 0.34 | 1641.03 |
| 454 | ORF1ab-1FS_681 | 16 | H- | AATRGATVVIGTSKFY | OH | 0.27 | 1641.89 |
| 455 | ORF1ab-1FS_682 | 18 | H- | TVVIGTSKFYGGWHNMLK | OH | 0.42 | 2038.41 |
| 456 | ORF1ab-1FS_683 | 16 | H- | FYGGWHNMLKTVYSDV | OH | 0.42 | 1917.18 |
| 457 | ORF1ab-1FS_684 | 18 | H- | NMLKTVYSDVENPHLMGW | OH | 0.36 | 2134.47 |
| 458 | ORF1ab-1FS_685 | 17 | H- | DVENPHLMGWDYPKCDR | OH | 0.17 | 2075.32 |
| 459 | ORF1ab-1FS_686 | 18 | H- | MGWDYPKCDRAMPNMLRI | OH | 0.34 | 2197.66 |
| 460 | ORF1ab-1FS_687 | 18 | H- | DRAMPNMLRIMASLVLAR | OH | 0.36 | 2058.57 |
| 461 | ORF1ab-1FS_688 | 18 | H- | RIMASLVLARKHTTCCSL | OH | 0.44 | 2003.49 |
| 462 | ORF1ab-1FS_689 | 17 | H- | ARKHTTCCSLSHRFYRL | OH | 0.23 | 2079.45 |
| 463 | ORF1ab-1FS_690 | 18 | H- | CSLSHRFYRLANECAQVL | OH | 0.38 | 2110.45 |
| 464 | ORF1ab-1FS_691 | 15 | H- | RLANECAQVLSEMVM | OH | 0.33 | 1694.03 |
| 465 | ORF1ab-1FS_692 | 18 | H- | CAQVLSEMVMCGGSLYVK | OH | 0.51 | 1918.36 |
| 466 | ORF1ab-1FS_693 | 18 | H- | VMCGGSLYVKPGGTSSGD | OH | 0.26 | 1714.94 |
| 467 | ORF1ab-1FS_694 | 15 | H- | VKPGGTSSGDATTAY | OH | 0.04 | 1411.49 |
| 468 | ORF1ab-1FS_695 | 17 | H- | TSSGDATTAYANSVFNI | OH | 0.19 | 1718.8 |
| 469 | ORF1ab-1FS_696 | 18 | H- | TAYANSVFNICQAVTANV | OH | 0.38 | 1886.12 |
| 470 | ORF1ab-1FS_697 | 18 | H- | NICQAVTANVNALLSTDG | OH | 0.3 | 1804.01 |
| 471 | ORF1ab-1FS_698 | 18 | H- | NVNALLSTDGNKIADKYV | OH | 0.12 | 1935.17 |
| 472 | ORF1ab-1FS_699 | 18 | H- | DGNKIADKYVRNLQHRLY | OH | 0 | 2203.49 |
| 473 | ORF1ab-1FS_700 | 17 | H- | YVRNLQHRLYECLYRNR | OH | 0.16 | 2296.65 |
| 474 | ORF1ab-1FS_701 | 17 | H- | RLYECLYRNRDVDTDFV | OH | 0.16 | 2177.43 |
| 475 | ORF1ab-1FS_702 | 18 | H- | RNRDVDTDFVNEFYAYLR | OH | 0.06 | 2293.49 |
| 476 | ORF1ab-1FS_703 | 18 | H- | FVNEFYAYLRKHFSMMIL | OH | 0.58 | 2309.79 |
| 477 | ORF1ab-1FS_704 | 18 | H- | LRKHFSMMILSDDAVVCF | OH | 0.51 | 2112.58 |
| 478 | ORF1ab-1FS_705 | 18 | H- | ILSDDAVVCFNSTYASQG | OH | 0.34 | 1890.06 |
| 479 | ORF1ab-1FS_706 | 18 | H- | CFNSTYASQGLVASIKNF | OH | 0.37 | 1950.21 |
| 480 | ORF1ab-1FS_707 | 16 | H- | QGLVASIKNFKSVLYY | OH | 0.39 | 1830.16 |
| 481 | ORF1ab-1FS_708 | 16 | H- | IKNFKSVLYYQNNVFM | OH | 0.38 | 2008.38 |
| 482 | ORF1ab-1FS_709 | 16 | H- | VLYYQNNVFMSEAKCW | OH | 0.46 | 1995.31 |
| 483 | ORF1ab-1FS_710 | 17 | H- | NVFMSEAKCWTETDLTK | OH | 0.23 | 2003.29 |
| 484 | ORF1ab-1FS_711 | 15 | H- | KCWTETDLTKGPHEF | OH | 0.18 | 1792 |
| 485 | ORF1ab-1FS_712 | 18 | H- | TDLTKGPHEFCSQHTMLV | OH | 0.33 | 2044.34 |
| 486 | ORF1ab-1FS_713 | 18 | H- | EFCSQHTMLVKQGDDYVY | OH | 0.28 | 2163.42 |
| 487 | ORF1ab-1FS_714 | 18 | H- | LVKQGDDYVYLPYPDPSR | OH | 0.23 | 2125.37 |
| 488 | ORF1ab-1FS_715 | 18 | H- | VYLPYPDPSRILGAGCFV | OH | 0.59 | 1967.32 |
| 489 | ORF1ab-1FS_716 | 15 | H- | SRILGAGCFVDDIVK | OH | 0.35 | 1592.88 |
| 490 | ORF1ab-1FS_717 | 17 | H- | AGCFVDDIVKTDGTLMI | OH | 0.42 | 1798.11 |
| 491 | ORF1ab-1FS_718 | 18 | H- | IVKTDGTLMIERFVSLAI | OH | 0.49 | 2006.44 |
| 492 | ORF1ab-1FS_719 | 17 | H- | MIERFVSLAIDAYPLTK | OH | 0.45 | 1967.36 |
| 493 | ORF1ab-1FS_720 | 16 | H- | LAIDAYPLTKHPNQEY | OH | 0.26 | 1873.1 |
| 494 | ORF1ab-1FS_721 | 18 | H- | PLTKHPNQEYADVFHLYL | OH | 0.37 | 2185.47 |
| 495 | ORF1ab-1FS_722 | 16 | H- | EYADVFHLYLQYIRKL | OH | 0.43 | 2071.41 |
| 496 | ORF1ab-1FS_723 | 18 | H- | HLYLQYIRKLHDELTGHM | OH | 0.35 | 2267.65 |
| 497 | ORF1ab-1FS_724 | 18 | H- | KLHDELTGHMLDMYSVML | OH | 0.41 | 2133.55 |
| 498 | ORF1ab-1FS_725 | 18 | H- | HMLDMYSVMLTNDNTSRY | OH | 0.26 | 2191.5 |
| 499 | ORF1ab-1FS_726 | 16 | H- | MLTNDNTSRYWEPEFY | OH | 0.23 | 2066.24 |
| 500 | ORF1ab-1FS_727 | 18 | H- | TSRYWEPEFYEAMYTPHT | OH | 0.3 | 2308.52 |
| 501 | ORF1ab-1FS_728 | 15 | H- | FYEAMYTPHTVLQAV | OH | 0.5 | 1770.04 |
| 502 | ORF1ab-1FS_729 | 15 | H- | YTPHTVLQAVGACVL | OH | 0.56 | 1571.86 |
| 503 | ORF1ab-1FS_886 | 18 | H- | RLQSLENVAFNVVNKGHF | OH | 0.23 | 2072.36 |
| 504 | ORF1ab-1FS_887 | 17 | H- | AFNVVNKGHFDGQQGEV | OH | 0.1 | 1845.99 |
| 505 | ORF1ab-1FS_888 | 15 | H- | GHFDGQQGEVPVSII | OH | 0.29 | 1582.74 |
| 506 | ORF1ab-1FS_889 | 18 | H- | QQGEVPVSIINNTVYTKV | OH | 0.28 | 1989.26 |
| 507 | ORF1ab-1FS_890 | 18 | H- | IINNTVYTKVDGVDVELF | OH | 0.37 | 2039.32 |
| 508 | ORF1ab-1FS_891 | 18 | H- | KVDGVDVELFENKTTLPV | OH | 0.21 | 2003.28 |
| 509 | ORF1ab-1FS_892 | 17 | H- | LFENKTTLPVNVAFELW | OH | 0.51 | 2021.35 |
| 510 | ORF1ab-1FS_893 | 18 | H- | LPVNVAFELWAKRNIKPV | OH | 0.42 | 2094.54 |
| 511 | ORF1ab-1FS_894 | 16 | H- | LWAKRNIKPVPEVKIL | OH | 0.36 | 1904.38 |
| 512 | ORF1ab-1FS_895 | 17 | H- | IKPVPEVKILNNLGVDI | OH | 0.4 | 1861.26 |
| 513 | ORF1ab-1FS_896 | 17 | H- | KILNNLGVDIAANTVIW | OH | 0.48 | 1854.18 |
| 514 | ORF1ab-1FS_897 | 18 | H- | VDIAANTVIWDYKRDAPA | OH | 0.24 | 2018.26 |
| 515 | ORF1ab-1FS_898 | 17 | H- | IWDYKRDAPAHISTIGV | OH | 0.33 | 1942.21 |
| 516 | ORF1ab-1FS_899 | 18 | H- | APAHISTIGVCSMTDIAK | OH | 0.4 | 1815.15 |
| 517 | ORF1ab-1FS_900 | 16 | H- | GVCSMTDIAKKPTETI | OH | 0.23 | 1694 |
| 518 | ORF1ab-1FS_901 | 18 | H- | DIAKKPTETICAPLTVFF | OH | 0.47 | 1994.39 |
| 519 | ORF1ab-1FS_902 | 18 | H- | TICAPLTVFFDGRVDGQV | OH | 0.47 | 1938.24 |
| 520 | ORF1ab-1FS_903 | 17 | H- | FFDGRVDGQVDLFRNAR | OH | 0.1 | 2012.22 |
| 521 | ORF1ab-1FS_904 | 15 | H- | GQVDLFRNARNGVLI | OH | 0.22 | 1671.92 |
| 522 | ORF1ab-1FS_905 | 18 | H- | FRNARNGVLITEGSVKGL | OH | 0.17 | 1931.23 |
| 523 | ORF1ab-1FS_906 | 17 | H- | LITEGSVKGLQPSVGPK | OH | 0.24 | 1710.01 |
| 524 | ORF1ab-1FS_907 | 17 | H- | KGLQPSVGPKQASLNGV | OH | 0.14 | 1679.94 |
| 525 | ORF1ab-1FS_908 | 18 | H- | GPKQASLNGVTLIGEAVK | OH | 0.2 | 1782.07 |
| 526 | ORF1ab-1FS_909 | 18 | H- | GVTLIGEAVKTQFNYYKK | OH | 0.21 | 2059.4 |
| 527 | ORF1ab-1FS_910 | 18 | H- | VKTQFNYYKKVDGVVQQL | OH | 0.19 | 2157.5 |
| 528 | ORF1ab-1FS_911 | 15 | H- | KKVDGVVQQLPETYF | OH | 0.2 | 1751.01 |
| 529 | ORF1ab-1FS_912 | 16 | H- | VVQQLPETYFTQSRNL | OH | 0.28 | 1923.16 |
| 530 | ORF1ab-1FS_913 | 16 | H- | ETYFTQSRNLQEFKPR | OH | 0.02 | 2044.26 |
| 531 | ORF1ab-1FS_914 | 18 | H- | SRNLQEFKPRSQMEIDFL | OH | 0.14 | 2238.56 |
| 532 | ORF1ab-1FS_915 | 18 | H- | PRSQMEIDFLELAMDEFI | OH | 0.38 | 2184.52 |
| 533 | ORF1ab-1FS_916 | 18 | H- | FLELAMDEFIERYKLEGY | OH | 0.34 | 2266.61 |
| 534 | ORF1ab-1FS_917 | 17 | H- | FIERYKLEGYAFEHIVY | OH | 0.41 | 2177.49 |
| 535 | ORF1ab-1FS_918 | 18 | H- | EGYAFEHIVYGDFSHSQL | OH | 0.33 | 2099.25 |
| 536 | ORF1ab-1FS_919 | 17 | H- | VYGDFSHSQLGGLHLLI | OH | 0.53 | 1856.12 |
| 537 | ORF1ab-1FS_920 | 17 | H- | SQLGGLHLLIGLAKRFK | OH | 0.4 | 1851.27 |
| 538 | ORF1ab-1FS_921 | 16 | H- | LLIGLAKRFKESPFEL | OH | 0.4 | 1861.26 |
| 539 | ORF1ab-1FS_922 | 16 | H- | KRFKESPFELEDFIPM | OH | 0.23 | 2013.35 |
| 540 | ORF1ab-1FS_923 | 18 | H- | PFELEDFIPMDSTVKNYF | OH | 0.39 | 2192.48 |
| 541 | ORF1ab-1FS_924 | 18 | H- | PMDSTVKNYFITDAQTGS | OH | 0.18 | 1975.17 |
| 542 | ORF1ab-1FS_925 | 18 | H- | YFITDAQTGSSKCVCSVI | OH | 0.44 | 1922.21 |
| 543 | ORF1ab-1FS_926 | 18 | H- | GSSKCVCSVIDLLLDDFV | OH | 0.51 | 1913.25 |
| 544 | ORF1ab-1FS_927 | 18 | H- | VIDLLLDDFVEIIKSQDL | OH | 0.48 | 2088.43 |
| 545 | ORF1ab-1FS_928 | 18 | H- | FVEIIKSQDLSVVSKVVK | OH | 0.34 | 2018.43 |
| 546 | ORF1ab-1FS_929 | 18 | H- | DLSVVSKVVKVTIDYTEI | OH | 0.34 | 2008.34 |
| 547 | ORF1ab-1FS_930 | 17 | H- | VKVTIDYTEISFMLWCK | OH | 0.57 | 2076.51 |
| 548 | ORF1ab-1FS_931 | 18 | H- | TEISFMLWCKDGHVETFY | OH | 0.5 | 2206.53 |
| 549 | ORF1ab-1FS_932 | 18 | H- | CKDGHVETFYPKLQSSQA | OH | 0.15 | 2038.27 |
| 550 | ORF1ab-1FS_933 | 17 | H- | FYPKLQSSQAWQPGVAM | OH | 0.41 | 1938.24 |
| 551 | ORF1ab-1FS_934 | 18 | H- | SQAWQPGVAMPNLYKMQR | OH | 0.28 | 2105.48 |
| 552 | ORF1ab-1FS_935 | 18 | H- | AMPNLYKMQRMLLEKCDL | OH | 0.35 | 2197.74 |
| 553 | ORF1ab-1FS_936 | 18 | H- | QRMLLEKCDLQNYGDSAT | OH | 0.12 | 2085.35 |
| 554 | ORF1ab-1FS_937 | 17 | H- | DLQNYGDSATLPKGIMM | OH | 0.25 | 1854.14 |
| 555 | ORF1ab-1FS_938 | 18 | H- | SATLPKGIMMNVAKYTQL | OH | 0.35 | 1966.4 |
| 556 | ORF1ab-1FS_939 | 17 | H- | MMNVAKYTQLCQYLNTL | OH | 0.46 | 2034.45 |
| 557 | ORF1ab-1FS_940 | 18 | H- | TQLCQYLNTLTLAVPYNM | OH | 0.56 | 2086.46 |
| 558 | ORF1ab-1FS_941 | 15 | H- | TLTLAVPYNMRVIHF | OH | 0.58 | 1775.15 |
| 559 | ORF1ab-1FS_942 | 18 | H- | VPYNMRVIHFGAGSDKGV | OH | 0.27 | 1947.25 |
| 560 | ORF1ab-1FS_943 | 18 | H- | HFGAGSDKGVAPGTAVLR | OH | 0.17 | 1739.95 |
| 561 | ORF1ab-1FS_944 | 18 | H- | GVAPGTAVLRQWLPTGTL | OH | 0.48 | 1837.15 |
| 562 | ORF1ab-1FS_945 | 16 | H- | LRQWLPTGTLLVDSDL | OH | 0.48 | 1827.11 |
| 563 | ORF1ab-1FS_946 | 18 | H- | TGTLLVDSDLNDFVSDAD | OH | 0.19 | 1896.98 |
| 564 | ORF1ab-1FS_947 | 18 | H- | DLNDFVSDADSTLIGDCA | OH | 0.22 | 1870.97 |
| 565 | ORF1ab-1FS_948 | 18 | H- | ADSTLIGDCATVHTANKW | OH | 0.27 | 1903.11 |
| 566 | ORF1ab-1FS_949 | 18 | H- | CATVHTANKWDLIISDMY | OH | 0.44 | 2081.4 |
| 567 | ORF1ab-1FS_950 | 17 | H- | KWDLIISDMYDPKTKNV | OH | 0.22 | 2066.41 |
| 568 | ORF1ab-1FS_951 | 17 | H- | DMYDPKTKNVTKENDSK | OH | -0.28 | 2013.22 |
| 569 | ORF1ab-1FS_952 | 17 | H- | KNVTKENDSKEGFFTYI | OH | 0 | 2020.23 |
| 570 | ORF1ab-1FS_953 | 18 | H- | DSKEGFFTYICGFIQQKL | OH | 0.39 | 2124.45 |
| 571 | ORF1ab-1FS_954 | 18 | H- | YICGFIQQKLALGGSVAI | OH | 0.58 | 1881.27 |
| 572 | ORF1ab-1FS_955 | 17 | H- | KLALGGSVAIKITEHSW | OH | 0.37 | 1810.13 |
| 573 | ORF1ab-1FS_956 | 18 | H- | VAIKITEHSWNADLYKLM | OH | 0.39 | 2132.52 |
| 574 | ORF1ab-1FS_957 | 16 | H- | SWNADLYKLMGHFAWW | OH | 0.59 | 2025.33 |
| 575 | ORF1ab-1FS_958 | 17 | H- | YKLMGHFAWWTAFVTNV | OH | 0.65 | 2071.44 |
| 576 | ORF1ab-1FS_959 | 18 | H- | AWWTAFVTNVNASSSEAF | OH | 0.43 | 1988.15 |
| 577 | ORF1ab-1FS_960 | 17 | H- | NVNASSSEAFLIGCNYL | OH | 0.37 | 1802 |
| 578 | ORF1ab-1FS_961 | 17 | H- | EAFLIGCNYLGKPREQI | OH | 0.34 | 1951.28 |
| 579 | ORF1ab-1FS_962 | 15 | H- | NYLGKPREQIDGYVM | OH | 0.15 | 1783.04 |
| 580 | ORF1ab-1FS_963 | 18 | H- | PREQIDGYVMHANYIFWR | OH | 0.36 | 2295.62 |
| 581 | ORF1ab-1FS_964 | 17 | H- | VMHANYIFWRNTNPIQL | OH | 0.49 | 2117.47 |
| 582 | ORF1ab-1FS_965 | 18 | H- | FWRNTNPIQLSSYSLFDM | OH | 0.46 | 2219.51 |
| 583 | ORF1ab-1FS_966 | 18 | H- | QLSSYSLFDMSKFPLKLR | OH | 0.37 | 2160.57 |
| 584 | ORF1ab-1FS_967 | 18 | H- | DMSKFPLKLRGTAVMSLK | OH | 0.26 | 2022.51 |
| 585 | ORF1ab-1FS_968 | 18 | H- | LRGTAVMSLKEGQINDMI | OH | 0.26 | 1976.35 |
| 586 | ORF1ab-1FS_969 | 18 | H- | LKEGQINDMILSLLSKGR | OH | 0.23 | 2015.41 |
| 587 | ORF1ab-1FS_970 | 18 | H- | MILSLLSKGRLIIRENNR | OH | 0.29 | 2126.6 |
| 588 | ORF1ab-1FS_971 | 18 | H- | GRLIIRENNRVVISSDVL | OH | 0.25 | 2053.4 |
| 589 | ORF1ab-1FS_972 | 18 | H- | IIRENNRVVISSDVLVNN | OH | 0.21 | 2054.34 |
| 590 | ORF3a_1 | 16 | H- | MDLFMRIFTIGTVTLK | OH | 0.58 | 1886.36 |
| 591 | ORF3a_2 | 15 | H- | IFTIGTVTLKQGEIK | OH | 0.37 | 1647.98 |
| 592 | ORF3a_3 | 18 | H- | TVTLKQGEIKDATPSDFV | OH | 0.17 | 1949.19 |
| 593 | ORF3a_4 | 18 | H- | IKDATPSDFVRATATIPI | OH | 0.31 | 1916.21 |
| 594 | ORF3a_5 | 18 | H- | FVRATATIPIQASLPFGW | OH | 0.61 | 1975.32 |
| 595 | ORF3a_6 | 18 | H- | PIQASLPFGWLIVGVALL | OH | 0.85 | 1894.34 |
| 596 | ORF3a_7 | 18 | H- | GWLIVGVALLAVFQSASK | OH | 0.62 | 1859.25 |
| 597 | ORF3a_8 | 18 | H- | LLAVFQSASKIITLKKRW | OH | 0.44 | 2102.6 |
| 598 | ORF3a_9 | 18 | H- | SKIITLKKRWQLALSKGV | OH | 0.28 | 2069.57 |
| 599 | ORF3a_10 | 18 | H- | RWQLALSKGVHFVCNLLL | OH | 0.62 | 2097.56 |
| 600 | ORF3a_11 | 16 | H- | GVHFVCNLLLLFVTVY | OH | 0.88 | 1837.26 |
| 601 | ORF3a_12 | 16 | H- | NLLLLFVTVYSHLLLV | OH | 0.91 | 1857.31 |
| 602 | ORF3a_13 | 18 | H- | VTVYSHLLLVAAGLEAPF | OH | 0.64 | 1900.25 |
| 603 | ORF3a_14 | 18 | H- | LVAAGLEAPFLYLYALVY | OH | 0.76 | 1986.39 |
| 604 | ORF3a_15 | 18 | H- | PFLYLYALVYFLQSINFV | OH | 0.89 | 2210.65 |
| 605 | ORF3a_16 | 18 | H- | VYFLQSINFVRIIMRLWL | OH | 0.82 | 2311.87 |
| 606 | ORF3a_17 | 17 | H- | FVRIIMRLWLCWKCRSK | OH | 0.63 | 2238.87 |
| 607 | ORF3a_18 | 15 | H- | LWLCWKCRSKNPLLY | OH | 0.63 | 1923.39 |
| 608 | ORF3a_19 | 18 | H- | KCRSKNPLLYDANYFLCW | OH | 0.42 | 2234.64 |
| 609 | ORF3a_20 | 17 | H- | LYDANYFLCWHTNCYDY | OH | 0.56 | 2204.43 |
| 610 | ORF3a_21 | 17 | H- | LCWHTNCYDYCIPYNSV | OH | 0.62 | 2094.38 |
| 611 | ORF3a_22 | 16 | H- | YDYCIPYNSVTSSIVI | OH | 0.56 | 1837.08 |
| 612 | ORF3a_23 | 18 | H- | YNSVTSSIVITSGDGTTS | OH | 0.23 | 1788.88 |
| 613 | ORF3a_24 | 17 | H- | VITSGDGTTSPISEHDY | OH | 0.18 | 1778.85 |
| 614 | ORF3a_25 | 18 | H- | TTSPISEHDYQIGGYTEK | OH | 0.1 | 2026.15 |
| 615 | ORF3a_26 | 16 | H- | DYQIGGYTEKWESGVK | OH | 0.08 | 1860.01 |
| 616 | ORF3a_27 | 18 | H- | YTEKWESGVKDCVVLHSY | OH | 0.28 | 2143.41 |
| 617 | ORF3a_28 | 18 | H- | VKDCVVLHSYFTSDYYQL | OH | 0.45 | 2180.47 |
| 618 | ORF3a_29 | 15 | H- | SYFTSDYYQLYSTQL | OH | 0.38 | 1879.01 |
| 619 | ORF3a_30 | 16 | H- | DYYQLYSTQLSTDTGV | OH | 0.25 | 1853.96 |
| 620 | ORF3a_31 | 18 | H- | STQLSTDTGVEHVTFFIY | OH | 0.41 | 2045.24 |
| 621 | ORF3a_32 | 18 | H- | GVEHVTFFIYNKIVDEPE | OH | 0.34 | 2136.39 |
| 622 | ORF3a_33 | 18 | H- | IYNKIVDEPEEHVQIHTI | OH | 0.28 | 2177.45 |
| 623 | ORF3a_34 | 17 | H- | PEEHVQIHTIDGSSGVV | OH | 0.23 | 1803.95 |
| 624 | ORF3a_35 | 18 | H- | HTIDGSSGVVNPVMEPIY | OH | 0.37 | 1915.16 |
| 625 | ORF3a_36 | 18 | H- | VVNPVMEPIYDEPTTTTS | OH | 0.32 | 1993.22 |
| 626 | ORF3a_37 | 18 | H- | PVMEPIYDEPTTTTSVPL | OH | 0.41 | 1990.26 |
| 627 | ORF8_1 | 16 | H- | MKFLVFLGIITTVAAF | OH | 0.8 | 1771.24 |
| 628 | ORF8_2 | 16 | H- | LGIITTVAAFHQECSL | OH | 0.56 | 1702.99 |
| 629 | ORF8_3 | 18 | H- | VAAFHQECSLQSCTQHQP | OH | 0.31 | 2014.23 |
| 630 | ORF8_4 | 18 | H- | SLQSCTQHQPYVVDDPCP | OH | 0.33 | 2017.23 |
| 631 | ORF8_5 | 18 | H- | QPYVVDDPCPIHFYSKWY | OH | 0.52 | 2257.56 |
| 632 | ORF8_6 | 17 | H- | CPIHFYSKWYIRVGARK | OH | 0.4 | 2124.55 |
| 633 | ORF8_7 | 17 | H- | KWYIRVGARKSAPLIEL | OH | 0.33 | 2000.42 |
| 634 | ORF8_8 | 18 | H- | ARKSAPLIELCVDEAGSK | OH | 0.14 | 1887.19 |
| 635 | ORF8_9 | 18 | H- | ELCVDEAGSKSPIQYIDI | OH | 0.3 | 1980.23 |
| 636 | ORF8_10 | 18 | H- | SKSPIQYIDIGNYTVSCL | OH | 0.41 | 2001.29 |
| 637 | ORF8_11 | 18 | H- | DIGNYTVSCLPFTINCQE | OH | 0.45 | 2017.27 |
| 638 | ORF8_12 | 18 | H- | CLPFTINCQEPKLGSLVV | OH | 0.58 | 1961.38 |
| 639 | ORF8_13 | 18 | H- | QEPKLGSLVVRCSFYEDF | OH | 0.33 | 2117.42 |
| 640 | ORF8_14 | 18 | H- | VVRCSFYEDFLEYHDVRV | OH | 0.35 | 2276.56 |
| 641 | ORF8_15 | 18 | H- | FYEDFLEYHDVRVVLDFI | OH | 0.5 | 2319.61 |
