# Supplementary material for: Induction of an early IFN-γ cellular response and high plasma levels of SDF-1α are inversely associated with COVID-19 severity and residence in rural areas in Kenyan patients
Source: PLoS One. 2025 Sep 11;20(9):e0316967. doi: 10.1371/journal.pone.0316967 (PMC12425234; doi:10.1371/journal.pone.0316967)
Supplement: S2 Table — The pooling strategy for the 10 peptide pools used. (DOCX) [file pone.0316967.s002.docx]

| **Table S2.** | |  |  |  |
| --- | --- | --- | --- | --- |
| **Pool** | **Region** | **Starting Peptide** | **Last Peptide** | **No of peptides** |
| 1 | S1 | S_1 | 93 | 93 |
| 2 | S2 | S_94 | 178 | 85 |
| 3 | M | M(ORF5) _1 | 31 | 31 |
| 4 | N | N(ORF9) _1 | 55 | 55 |
| 5 | NSP 3 - B | ORF1a/1ab_207 | 306 | 100 |
| 6 | NSP 3 - C | ORF1a/1ab_307 | 379 | 73 |
| 7 | NSP 12 -B | ORF1ab-1FS_665 | 729 | 65 |
| 8 | NSP 15-16 | ORF1ab-1FS_886 | 972 | 87 |
| 9 | ORF3 | ORF3a_1 | 37 | 37 |
| 10 | ORF8 | ORF8_1 | 15 | 15 |
