# Supplementary material for: Induction of an early IFN-γ cellular response and high plasma levels of SDF-1α are inversely associated with COVID-19 severity and residence in rural areas in Kenyan patients
Source: PLoS One. 2025 Sep 11;20(9):e0316967. doi: 10.1371/journal.pone.0316967 (PMC12425234; doi:10.1371/journal.pone.0316967)
Supplement: S3 Table — Number of participants sampled for each peptide across different clinical phenotypes (asymptomatic, mild/moderate, severe) and time points (Day 0, 7, 14, 28). (DOCX) [file pone.0316967.s003.docx]

**Table S3.**

|  | **Spike**  **(n=171)** | **M**  **(n=136)** | **NP**  **(n=162)** | **NSP**  **(n=100)** | **ORF**  **(n=90)** |
| --- | --- | --- | --- | --- | --- |
| **Day 0** |  |  |  |  |  |
| Asymptomatic | 22 | 15 | 21 | 11 | 13 |
| Mild/moderate | 14 | 11 | 14 | 7 | 8 |
| Severe | 10 | 5 | 5 | 1 | 1 |
| **Day 7** |  |  |  |  |  |
| Asymptomatic | 14 | 10 | 14 | 10 | 13 |
| Mild/moderate | 15 | 14 | 15 | 9 | 9 |
| Severe | 15 | 12 | 14 | 4 | 5 |
| **Day 14** |  |  |  |  |  |
| Asymptomatic | 13 | 10 | 12 | 10 | 10 |
| Mild/moderate | 16 | 16 | 16 | 11 | 12 |
| Severe | 12 | 8 | 12 | 3 | 3 |
| **Day 28** |  |  |  |  |  |
| Asymptomatic | 13 | 10 | 13 | 9 | 10 |
| Mild/moderate | 16 | 16 | 16 | 11 | 12 |
| Severe | 11 | 9 | 10 | 4 | 4 |
