# Supplementary material for: Induction of an early IFN-γ cellular response and high plasma levels of SDF-1α are inversely associated with COVID-19 severity and residence in rural areas in Kenyan patients
Source: PLoS One. 2025 Sep 11;20(9):e0316967. doi: 10.1371/journal.pone.0316967 (PMC12425234; doi:10.1371/journal.pone.0316967)
Supplement: S4 Table — (DOCX) [file pone.0316967.s004.docx]

**Table S4.**

|  | **Odds ratio (95% CI)** | **P value** |
| --- | --- | --- |
| Age | 1.01 (0.99-1.02) | 0.515 |
| Sex |  |  |
| Female | Reference |  |
| Male | 1.06 (0.64-1.74) | 0.831 |
| Severity |  |  |
| Asymptomatic | Reference |  |
| Mild/moderate | 0.97 (0.52-1.8) | 0.924 |
| Severe | 1.32 (0.71-2.47) | 0.385 |
