# Supplementary material for: Induction of an early IFN-γ cellular response and high plasma levels of SDF-1α are inversely associated with COVID-19 severity and residence in rural areas in Kenyan patients
Source: PLoS One. 2025 Sep 11;20(9):e0316967. doi: 10.1371/journal.pone.0316967 (PMC12425234; doi:10.1371/journal.pone.0316967)
Supplement: S5 Table — Bold p values are significant, p < 0.05. (DOCX) [file pone.0316967.s005.docx]

**Table S5.**

| **Comparisons** | **Spike peptide** | | | **M peptide** | | | **NP peptide** | | | **NSP peptide** | | | **ORF peptide** | | |
| --- | --- | --- | --- | --- | --- | --- | --- | --- | --- | --- | --- | --- | --- | --- | --- |
|  | **Mean Diff.** | **CI (95%)** | **pvalue** | **Mean Diff.** | **CI (95%)** | **p value** | **Mean Diff.** | **CI (95%)** | **p value** | **Mean Diff.** | **CI (95%)** | **p value** | **Mean Diff.** | **CI (95%)** | **p value** |
| **Day 0** |  |  |  |  |  |  |  |  |  |  |  |  |  |  |  |
| Asymptomatic vs. Mild/moderate | 0.139 | -0.409 to 0.687 | 0.800 | 0.204 | -0.323 to 0.732 | 0.603 | 0.133 | -0.396 to 0.661 | 0.809 | 0.375 | -0.431 to 1.182 | 0.447 | 0.348 | -0.299 to 0.997 | 0.365 |
| Asymptomatic vs. Severe | 0.690 | 0.0419 to 1.338 | **0.037** | 0.062 | -0.928 to 1.053 | 0.980 | 0.417 | -0.794 to 1.627 | 0.544 | 1.144 |  |  | 0.816 |  |  |
| Mild/moderate vs. Severe | 0.551 | -0.191 to 1.293 | 0.170 | -0.142 | -1.135 to 0.851 | 0.903 | 0.284 | -0.915 to 1.483 | 0.758 | 0.768 |  |  | 0.467 |  |  |
| **Day 7** |  |  |  |  |  |  |  |  |  |  |  |  |  |  |  |
| Asymptomatic vs. Mild/moderate | 0.273 | -0.122 to 0.668 | 0.218 | 0.394 | -0.202 to 0.990 | 0.237 | 0.212 | -0.249 to 0.672 | 0.494 | -0.028 | -0.725 to 0.668 | 0.993 | -0.030 | -0.571 to 0.511 | 0.989 |
| Asymptomatic vs. Severe | 0.360 | -0.173 to 0.893 | 0.232 | -0.269 | -0.917 to 0.378 | 0.553 | 0.235 | -0.354 to 0.824 | 0.588 | 0.223 | -0.833 to 1.280 | 0.821 | 0.043 | -0.833 to 0.919 | 0.988 |
| Mild/moderate vs. Severe | 0.087 | -0.410 to 0.584 | 0.900 | -0.663 | -1.221 to -0.105 | **0.018** | 0.024 | -0.503 to 0.550 | 0.993 | 0.252 | -0.832 to 1.336 | 0.690 | 0.073 | -0.808 to 0.955 | 0.967 |
| **Day 14** |  |  |  |  |  |  |  |  |  |  |  |  |  |  |  |
| Asymptomatic vs. Mild/moderate | -0.190 | -0.658 to 0.278 | 0.576 | 0.201 | -0.438 to 0.840 | 0.705 | -0.091 | -0.581 to 0.399 | 0.890 | -0.187 | -0.870 to 0.497 | 0.769 | -0.044 | -0.595 to 0.507 | 0.978 |
| Asymptomatic vs. Severe | -0.105 | -0.640 to 0.430 | 0.876 | 0.061 | -0.775 to 0.896 | 0.981 | -0.041 | -0.625 to 0.544 | 0.983 | -0.013 | -0.680 to 0.654 | 0.999 | -0.064 | -0.813 to 0.684 | 0.955 |
| Mild/moderate vs. Severe | 0.085 | -0.413 to 0.583 | 0.904 | -0.140 | -0.902 to 0.621 | 0.876 | 0.050 | -0.521 to 0.621 | 0.973 | 0.174 | -0.427 to 0.774 | 0.710 | -0.020 | -0.763 to 0.722 | 0.996 |
| **Day 28** |  |  |  |  |  |  |  |  |  |  |  |  |  |  |  |
| Asymptomatic vs. Mild/moderate | 0.048 | -0.522 to 0.618 | 0.976 | 0.054 | -0.589 to 0.697 | 0.975 | -0.003 | -0.460 to 0.454 | 1.000 | 0.143 | -0.687 to 0.973 | 0.899 | -0.085 | -0.833 to 0.663 | 0.956 |
| Asymptomatic vs. Severe | -0.026 | -0.714 to 0.662 | 0.995 | -0.572 | -1.327 to 0.183 | 0.157 | -0.249 | -0.962 to 0.464 | 0.640 | -0.320 | -2.323 to 1.682 | 0.840 | -0.275 | -2.135 to 1.586 | 0.866 |
| Mild/moderate vs. Severe | -0.074 | '-0.804 to 0.656 | 0.965 | -0.626 | -1.301 to 0.048 | 0.071 | -0.246 | -0.964 to 0.472 | 0.653 | -0.463 | -2.417 to 1.491 | 0.721 | -0.190 | -2.038 to 1.658 | 0.933 |
|  | **Spike peptide** | | | **M peptide** | | | **NP peptide** | | | **NSP peptide** | | | **ORF peptide** | | |
| **Comparisons** | **Mean Diff.** | **CI (95%)** | **p value** | **Mean Diff.** | **CI (95%)** | **p value** | **Mean Diff.** | **CI (95%)** | **p value** | **Mean Diff.** | **CI (95%)** | **p value** | **Mean Diff.** | **CI (95%)** | **p value** |
| **Asymptomatic** |  |  |  |  |  |  |  |  |  |  |  |  |  |  |  |
| 0 vs. 7 | -0.138 | -0.392 to 0.117 | 0.417 | -0.228 | -0.628 to 0.172 | 0.343 | -0.179 | -0.426 to 0.067 | 0.193 | 0.071 | -0.320 to 0.462 | 0.939 | -0.038 | -0.133 to 0.058 | 0.657 |
| 0 vs. 14 | 0.106 | -0.192 to 0.403 | 0.722 | -0.491 | -0.950 to -0.031 | **0.036** | -0.059 | -0.387 to 0.268 | 0.947 | 0.123 | -0.206 to 0.451 | 0.661 | -0.135 | -0.401 to 0.132 | 0.436 |
| 0 vs. 28 | 0.060 | -0.198 to 0.319 | 0.898 | -0.114 | -0.547 to 0.319 | 0.843 | 0.005 | -0.261 to 0.271 | >0.999 | 0.242 | -0.064 to 0.548 | 0.128 | 0.104 | -0.267 to 0.475 | 0.818 |
| 7 vs. 14 | 0.243 | 0.0807 to 0.406 | **0.004** | -0.263 | -0.500 to -0.026 | **0.030** | 0.120 | -0.035 to 0.275 | 0.150 | 0.052 | -0.181 to 0.284 | 0.897 | -0.097 | -0.348 to 0.154 | 0.640 |
| 7 vs. 28 | 0.198 | 0.064 to 0.332 | **0.004** | 0.114 | -0.182 to 0.409 | 0.641 | 0.184 | 0.063 to 0.306 | **0.004** | 0.171 | -0.179 to 0.520 | 0.447 | 0.142 | -0.219 to 0.502 | 0.627 |
| 14 vs. 28 | -0.046 | -0.202 to 0.111 | 0.824 | 0.377 | -0.005 to 0.759 | 0.053 | 0.064 | -0.051 to 0.180 | 0.379 | 0.119 | -0.244 to 0.482 | 0.726 | 0.238 | -0.153 to 0.630 | 0.293 |
| **Mild/moderate** |  |  |  |  |  |  |  |  |  |  |  |  |  |  |  |
| 0 vs. 7 | -0.004 | -0.384 to 0.376 | 0.999 | -0.038 | -0.283 to 0.207 | 0.962 | -0.100 | -0.374 to 0.174 | 0.712 | -0.333 | -0.981 to 0.315 | 0.367 | -0.416 | -0.771 to -0.061 | **0.024** |
| 0 vs. 14 | -0.223 | -0.470 to 0.024 | 0.082 | -0.494 | -0.815 to -0.173 | **0.004** | -0.283 | -0.472 to -0.092 | **0.004** | -0.439 | -0.976 to 0.097 | 0.105 | -0.527 | -1.067 to 0.014 | 0.056 |
| 0 vs. 28 | -0.030 | -0.235 to 0.174 | 0.971 | -0.264 | -0.547 to 0.019 | 0.069 | -0.131 | -0.336 to 0.075 | 0.290 | 0.010 | -0.835 to 0.855 | >0.999 | -0.330 | -0.949 to 0.290 | 0.364 |
| 7 vs. 14 | -0.220 | -0.431 to -0.009 | **0.040** | -0.456 | -0.623 to -0.289 | **<0.0001** | -0.182 | -0.336 to -0.029 | **0.018** | -0.107 | -0.460 to 0.247 | 0.772 | -0.111 | -0.434 to 0.213 | 0.703 |
| 7 vs. 28 | -0.027 | -0.378 to 0.324 | 0.996 | -0.226 | -0.383 to -0.069 | **0.005** | -0.030 | -0.165 to 0.105 | 0.913 | 0.342 | -0.173 to 0.858 | 0.224 | 0.087 | -0.362 to 0.535 | 0.923 |
| 14 vs. 28 | 0.193 | -0.041 to 0.427 | 0.126 | 0.230 | 0.083 to 0.376 | **0.002** | 0.152 | 0.0727 to 0.231 | **0.000** | 0.449 | 0.039 to 0.859 | **0.032** | 0.197 | -0.071 to 0.466 | 0.180 |
| **Severe** |  |  |  |  |  |  |  |  |  |  |  |  |  |  |  |
| 0 vs. 7 | -0.468 | -0.910 to '-0.026 | **0.038** | -0.559 | '-1.558 to 0.439 | 0.246 | -0.361 | -1.865 to 1.143 | 0.770 | -0.849 |  |  | -0.810 |  |  |
| 0 vs. 14 | -0.689 | -1.158 to '-0.221 | **0.006** | -0.493 | -1.302 to 0.317 | 0.203 | -0.516 | -1.756 to 0.723 | 0.430 | -1.034 |  |  | -1.014 |  |  |
| 0 vs. 28 | -0.656 | -1.072 to '-0.240 | **0.004** | -0.749 | -1.842 to 0.344 | 0.151 | -0.661 | -1.817 to 0.495 | 0.235 | -1.222 |  |  | -0.987 |  |  |
| 7 vs. 14 | -0.221 | -0.488 to 0.046 | 0.116 | 0.067 | -0.350 to 0.484 | 0.949 | -0.156 | -0.474 to 0.163 | 0.485 | -0.185 | -1.422 to 1.052 | 0.753 | -0.204 | -2.065 to 1.657 | 0.867 |
| 7 vs. 28 | -0.188 | -0.508 to 0.133 | 0.331 | -0.190 | -0.545 to 0.166 | 0.379 | -0.300 | -0.707 to 0.107 | 0.169 | -0.373 | -1.888 to 1.142 | 0.673 | -0.177 | -1.709 to 1.356 | 0.939 |
| 14 vs. 28 | 0.033 | -0.342 to 0.409 | 0.993 | -0.256 | -0.617 to 0.104 | 0.175 | -0.145 | -0.495 to 0.206 | 0.593 | -0.188 | -3.333 to 2.957 | 0.971 | 0.028 | -2.739 to 2.795 | 1.000 |
