# Supplementary figures and images for: Induction of an early IFN-γ cellular response and high plasma levels of SDF-1α are inversely associated with COVID-19 severity and residence in rural areas in Kenyan patients
Source: PLoS One. 2025 Sep 11;20(9):e0316967. doi: 10.1371/journal.pone.0316967 (PMC12425234; doi:10.1371/journal.pone.0316967)

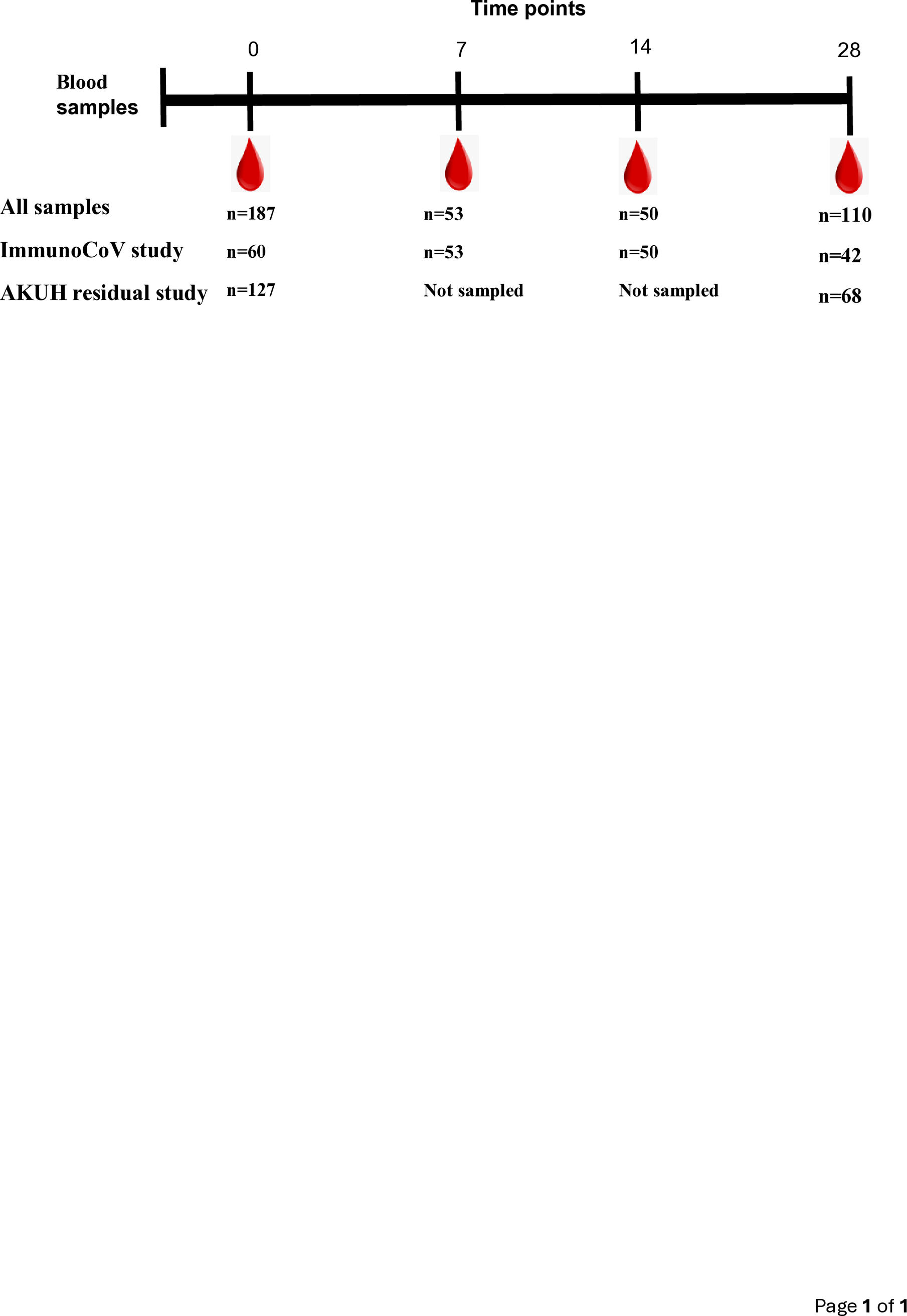

Supplement: S1 Fig — (TIF) [file pone.0316967.s006.tif]

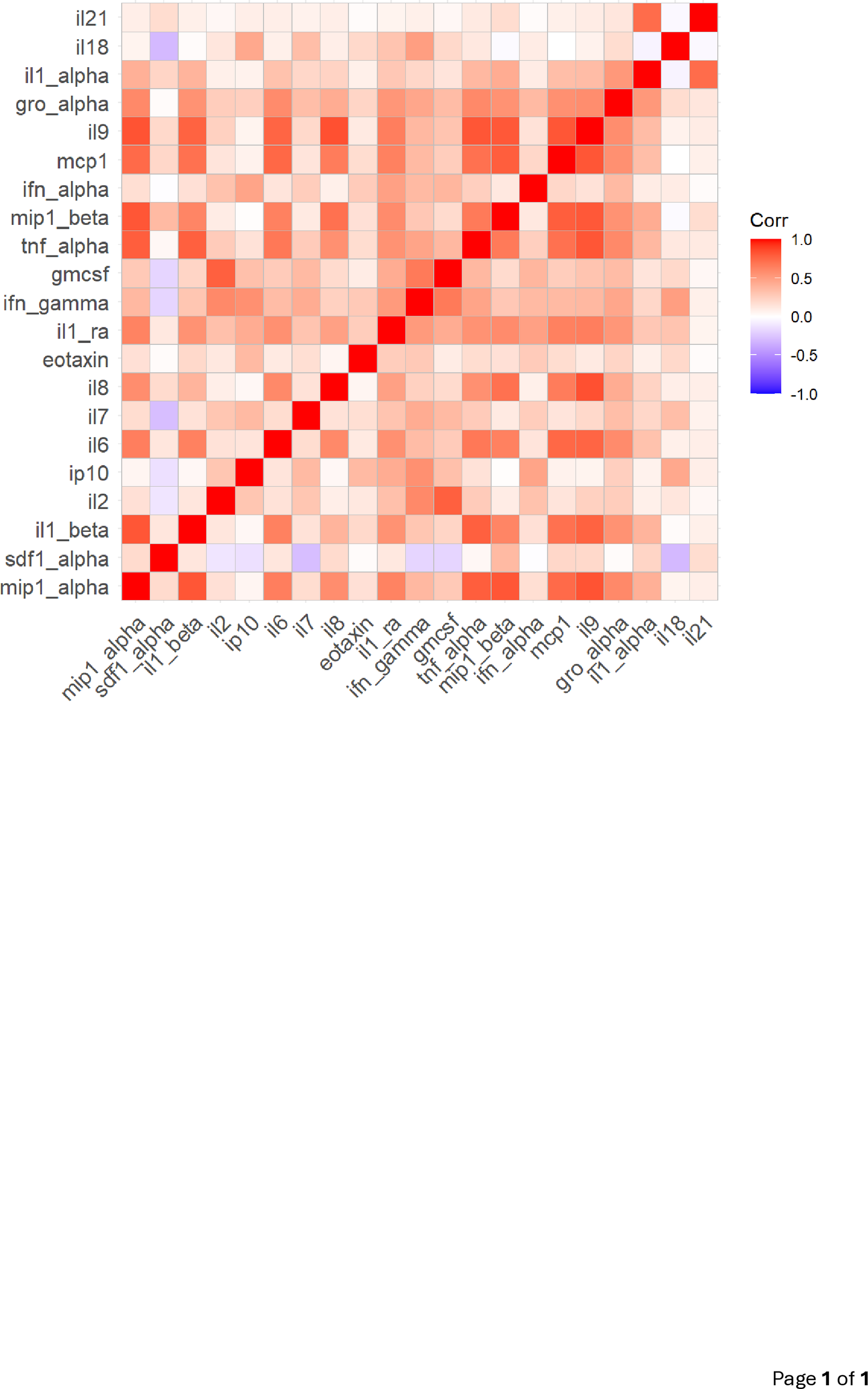

Supplement: S2 Fig — (TIF) [file pone.0316967.s007.tif]

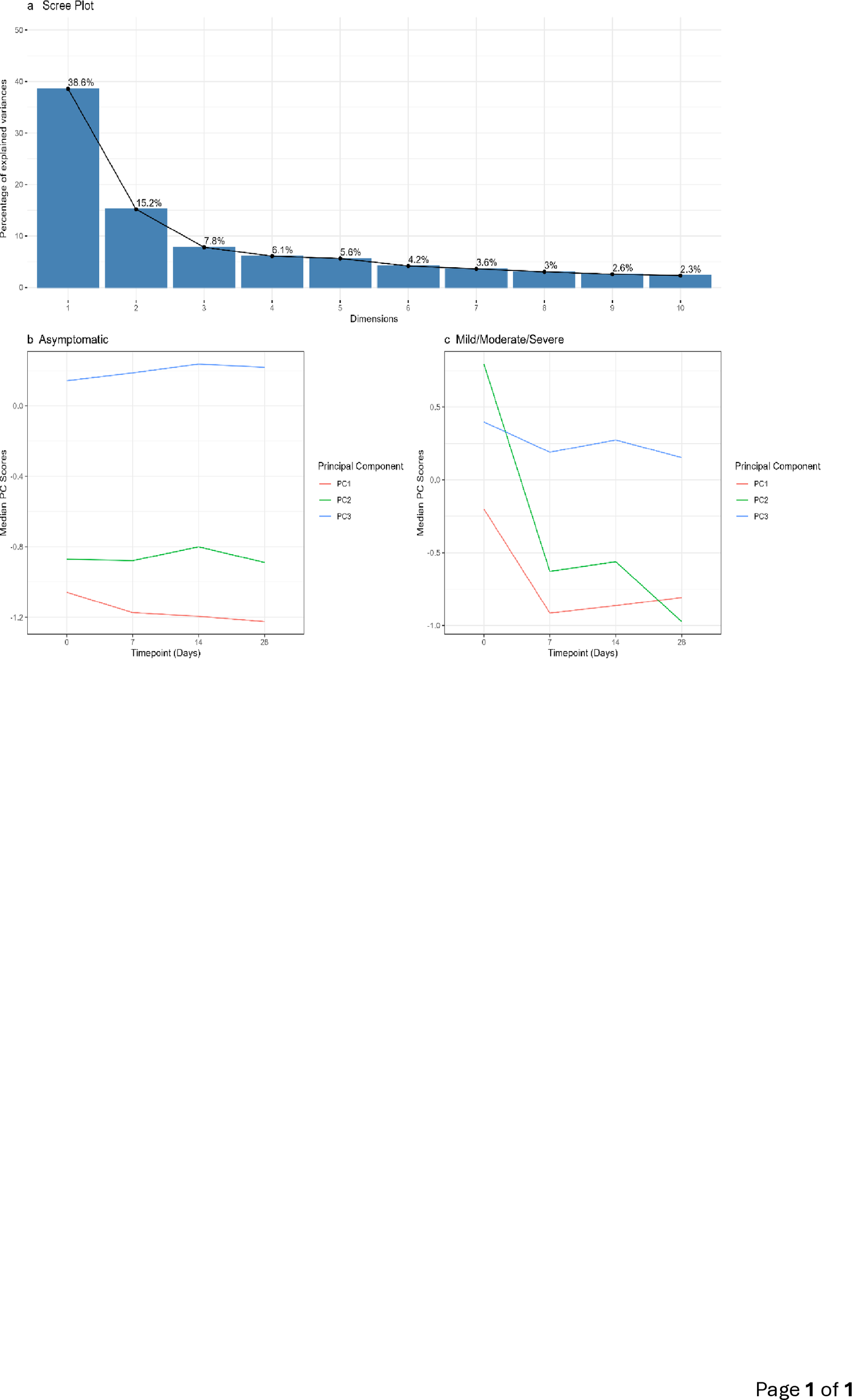

Supplement: S3 Fig — (TIF) [file pone.0316967.s008.tif]

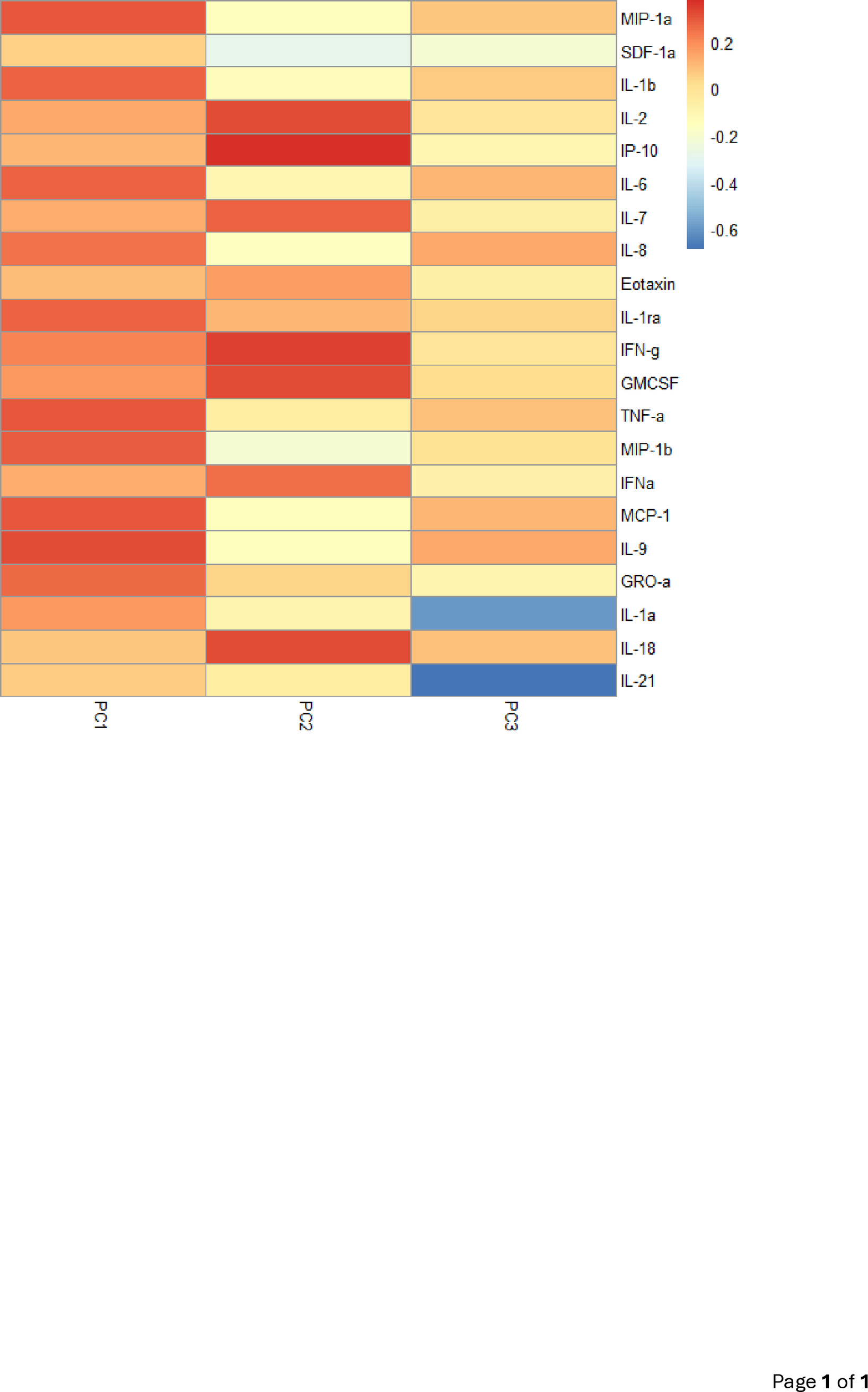

Supplement: S4 Fig — The color scale indicates the loading value, with red indicating a higher positive loading, blue a higher negative loading and light-yellow minimal loading. (TIF) [file pone.0316967.s009.tif]

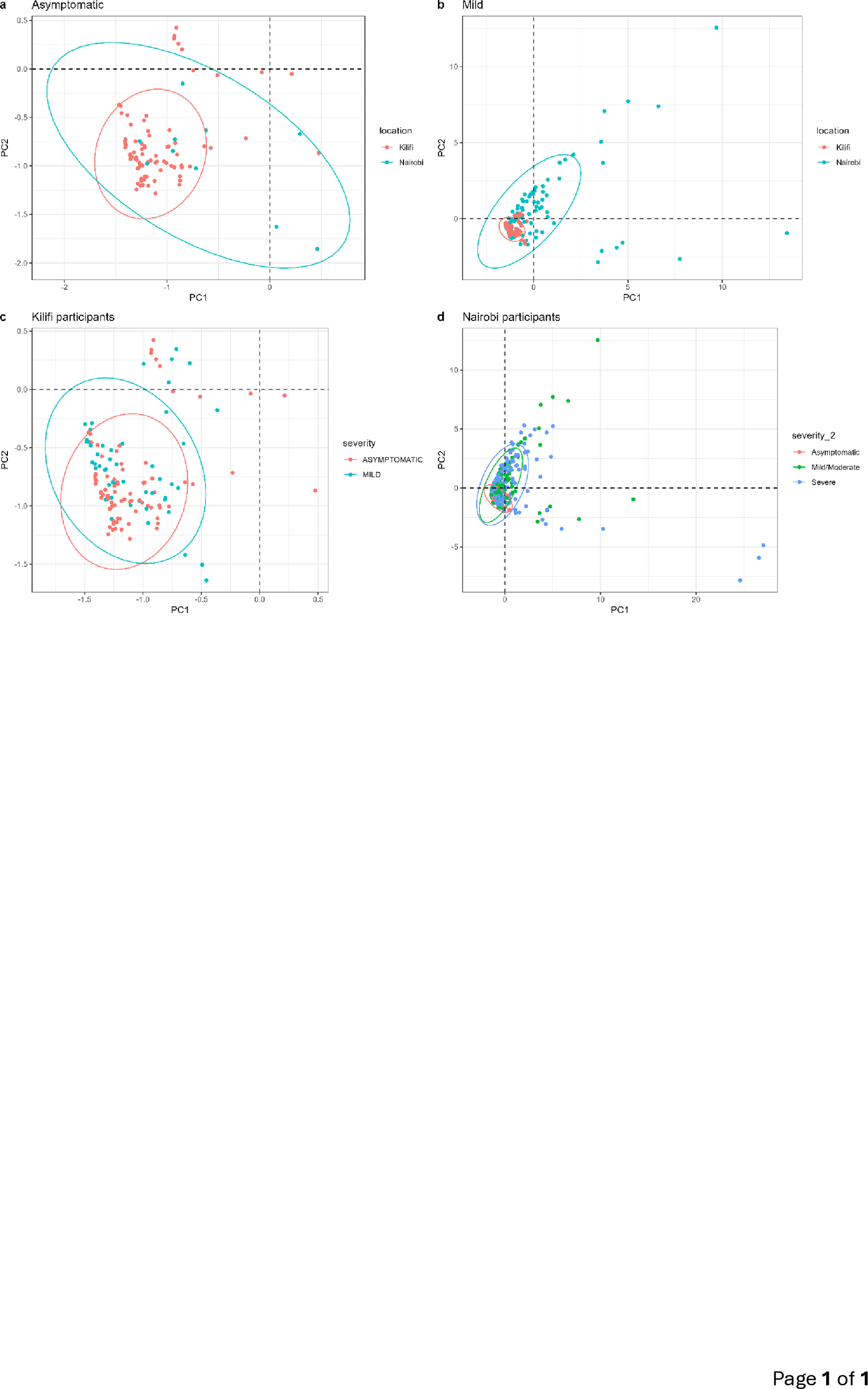

Supplement: S5 Fig — (a) Asymptomatic participants, (b) mild cases, (c) participants from Kilifi, and (d) participants from Nairobi. Each point represents an individual’s cytokine measurement, allowing for a visual assessment of cytokine variability across different groups based on location and clinical presentation. (TIF) [file pone.0316967.s010.tif]
