# Supplementary material for: Induction of an early IFN-γ cellular response and high plasma levels of SDF-1α are inversely associated with COVID-19 severity and residence in rural areas in Kenyan patients
Source: PLoS One. 2025 Sep 11;20(9):e0316967. doi: 10.1371/journal.pone.0316967 (PMC12425234; doi:10.1371/journal.pone.0316967)
Supplement: Attachment — Submitted filename: PONE-D-24-57722.pdf [file pone.0316967.s013.pdf]

PLOS ONE

Induction of an early IFN-γ cellular response and high plasma levels of SDF-1α are inversely associated with COVID-19 severity and residence in rural areas in Kenyan patients

--Manuscript Draft--

**Manuscript Number:**	PONE-D-24-57722
**Article Type:**	Research Article
**Full Title:**	Induction of an early IFN-γ cellular response and high plasma levels of SDF-1α are inversely associated with COVID-19 severity and residence in rural areas in Kenyan patients
**Short Title:**	Early IFN-γ response and high SDF-1α levels linked to Lower COVID-19 Severity in rural Kenyan patients
**Corresponding Author:**	perpetual Njeri Wanjiku<br>KEMRI-Wellcome Trust Research Programme: Centre for Geographic Medicine Research Coast<br>Kilifi, KENYA
**Keywords:**	COVID-19, SARS-CoV-2, IFN-γ-secreting cells, cytokines, chemokines, kinetics, Sub-Saharan Africa.
**Abstract:**	Introduction<br>COVID-19 was less severe in Sub-Saharan Africa (SSA) compared with Europe and North America. It is unclear whether these differences could be explained immunologically. Here we determined the levels of ex vivo SARS-CoV-2 peptide-specific IFN-γ producing cells, and levels of plasma cytokines and chemokines over the first month of COVID-19 diagnosis among Kenyan COVID-19 patients from urban and rural areas.<br>Methods<br>Between June 2020 and August 2022, we recruited and longitudinally monitored 188 COVID-19 patients from two regions in Kenya, Nairobi (urban, n = 152) and Kilifi (rural, n = 36), with varying levels of disease severity – severe, mild/moderate, and asymptomatic. IFN-γ secreting cells were enumerated at 0-, 7-, 14- and 28-days post diagnosis by an ex vivo enzyme-linked immunospot (ELISpot) assay following in vitro stimulation of peripheral blood mononuclear cells (PBMCs) with overlapping peptides from several SARS-CoV-2 proteins. A multiplexed binding assay was used to measure the levels of 22 plasma cytokines and chemokines.<br>Results<br>Higher frequencies of IFN-γ-secreting cells against the SARS-CoV-2 spike peptides were observed on the day of diagnosis among the asymptomatic compared to the patients with severe COVID-19. Higher concentrations of 17 of the 22 cytokines and chemokines measured were positively associated with severe disease, and in particular interleukin (IL)-8, IL-18 and IL-1ra ($p<0.0001$), while a lower concentration of SDF-1α was associated with severe disease ($p<0.0001$). Concentrations of 8 and 16 cytokines and chemokines including IL-18 were higher among Nairobi asymptomatic and mild patients compared to their respective Kilifi counterparts. Conversely, the concentrations for SDF-1α were higher in rural Kilifi compared to Nairobi ($p=0.012$).<br>Conclusion<br>In Kenya, as seen elsewhere, pro-inflammatory cytokines and chemokines were associated with severe COVID-19, while an early IFN-γ cellular response to overlapping SARS-CoV-2 spike peptides was associated with reduced risk of disease. Living in urban Nairobi (compared with rural Kilifi) was associated with increased levels of pro-inflammatory cytokines/chemokines.
**Order of Authors:**	perpetual Njeri Wanjiku
	Benedict Orindi
	John Kimotho
	Shahin Sayed

Powered by Editorial Manager® and ProduXion Manager® from Aries Systems Corporation

	Reena Shah
	Mansoor Saleh
	Jedidah Mwacharo
	Christopher Maronga
	Vivianne Olouch
	Ann Karanu
	Jasmit Shah
	Zaitun Nneka
	Lynette Isabella Ochola-Oyier
	Abdirahman I. Abdi
	Susanna Dunachie
	Philip Bejon
	Eunice Nduati
	Francis M. Ndungu
**Additional Information:**	
**Question**	**Response**
**Financial Disclosure**<br><br>Enter a financial disclosure statement that describes the sources of funding for the work included in this submission. Review the submission guidelines for detailed requirements. View published research articles from *PLOS ONE* for specific examples.<br><br>This statement is required for submission and **will appear in the published article** if the submission is accepted. Please make sure it is accurate.<br><br>**Funded studies**<br>Enter a statement with the following details:<br>• Initials of the authors who received each award<br>• Grant numbers awarded to each author<br>• The full name of each funder<br>• URL of each funder website<br>• Did the sponsors or funders play any role in the study design, data collection and analysis, decision to publish, or preparation of the manuscript?<br><br>Did you receive funding for this work?	Yes

Powered by Editorial Manager® and ProduXion Manager® from Aries Systems Corporation

Please add funding details.<br>as follow-up to "**Financial Disclosure**<br><br>Enter a financial disclosure statement that describes the sources of funding for the work included in this submission. Review the submission guidelines for detailed requirements. View published research articles from *PLOS ONE* for specific examples.<br><br>This statement is required for submission and **will appear in the published article** if the submission is accepted. Please make sure it is accurate.<br><br>**Funded studies**<br>Enter a statement with the following details:<br>• Initials of the authors who received each award<br>• Grant numbers awarded to each author<br>• The full name of each funder<br>• URL of each funder website<br>• Did the sponsors or funders play any role in the study design, data collection and analysis, decision to publish, or preparation of the manuscript?<br><br>Did you receive funding for this work?"	This study/project is funded by the EDCTP2 Programme (grant number RIA2020EF-3042) which is supported by the European Union, and the Swedish International Development Cooperation Agency.<br><br>https://www.edctp.org/projects-2/edctp2-projects/mobilisation-funding-covid-19-research-sub-saharan-africa/<br><br>FMN received this funding<br><br>SJD is supported by an NIHR Global Health Research Professorship (NIHR300791).<br><br>The funders had no role in study design, data collection and analysis, decision to publish, or preparation of the manuscript.
Please select the country of your main research funder (please select carefully as in some cases this is used in fee calculation).<br>as follow-up to "**Financial Disclosure**<br><br>Enter a financial disclosure statement that describes the sources of funding for the work included in this submission. Review the submission guidelines for detailed requirements. View published research articles from *PLOS ONE* for specific examples.<br><br>This statement is required for submission and **will appear in the published article** if the submission is accepted. Please make sure it is accurate.	NETHERLANDS - NL

**Funded studies**<br>Enter a statement with the following details:<br>• Initials of the authors who received each award<br>• Grant numbers awarded to each author<br>• The full name of each funder<br>• URL of each funder website<br>• Did the sponsors or funders play any role in the study design, data collection and analysis, decision to publish, or preparation of the manuscript?<br><br>Did you receive funding for this work?"	
**Competing Interests**<br><br>Use the instructions below to enter a competing interest statement for this submission. On behalf of all authors, disclose any competing interests that could be perceived to bias this work—acknowledging all financial support and any other relevant financial or non-financial competing interests.<br><br>This statement is required for submission and **will appear in the published article** if the submission is accepted. Please make sure it is accurate and that any funding sources listed in your Funding Information later in the submission form are also declared in your Financial Disclosure statement.<br><br>View published research articles from *PLOS ONE* for specific examples.	The authors have declared that no competing interests exist.

**NO authors have competing interests**<br><br>Enter: *The authors have declared that no competing interests exist.*<br><br>**Authors with competing interests**<br><br>Enter competing interest details beginning with this statement:<br><br>*I have read the journal's policy and the authors of this manuscript have the following competing interests: [insert competing interests here]*<br><br>* typeset	
**Ethics Statement**<br><br>Enter an ethics statement for this submission. This statement is required if the study involved:<br><br>• Human participants<br>• Human specimens or tissue<br>• Vertebrate animals or cephalopods<br>• Vertebrate embryos or tissues<br>• Field research<br><br>Write "N/A" if the submission does not require an ethics statement.<br><br>General guidance is provided below. Consult the submission guidelines for detailed instructions. **Make sure that all information entered here is included in the Methods section of the manuscript.**	The study obtained ethical approval from the Kenya Medical Research Institute's Scientific and Ethics Review Unit (KEMRI SERU; protocol no. 4081) and the Aga Khan University, Nairobi, Institutional Ethics Review Committee (protocol no. 2020 IERC-55 V5 and protocol no. 2020 IERC-135 V2). Written informed consent was obtained from all willing patients before their enrolment into the study.

Powered by Editorial Manager® and ProduXion Manager® from Aries Systems Corporation

**Format for specific study types**<br><br>**Human Subject Research (involving human participants and/or tissue)**<br>• Give the name of the institutional review board or ethics committee that approved the study<br>• Include the approval number and/or a statement indicating approval of this research<br>• Indicate the form of consent obtained (written/oral) or the reason that consent was not obtained (e.g. the data were analyzed anonymously)<br><br>**Animal Research (involving vertebrate animals, embryos or tissues)**<br>• Provide the name of the Institutional Animal Care and Use Committee (IACUC) or other relevant ethics board that reviewed the study protocol, and indicate whether they approved this research or granted a formal waiver of ethical approval<br>• Include an approval number if one was obtained<br>• If the study involved *non-human primates*, add *additional details* about animal welfare and steps taken to ameliorate suffering<br>• If anesthesia, euthanasia, or any kind of animal sacrifice is part of the study, include briefly which substances and/or methods were applied<br><br>**Field Research**<br><br>Include the following details if this study involves the collection of plant, animal, or other materials from a natural setting:<br>• Field permit number<br>• Name of the institution or relevant body that granted permission	
**Data Availability**<br><br>Authors are required to make all data underlying the findings described fully available, without restriction, and from the time of publication. PLOS allows rare exceptions to address legal and ethical concerns. See the PLOS Data Policy and FAQ for detailed information.	Yes - all data are fully available without restriction

A Data Availability Statement describing where the data can be found is required at submission. Your answers to this question constitute the Data Availability Statement and **will be published in the article**, if accepted.<br><br>**Important:** Stating 'data available on request from the author' is not sufficient. If your data are only available upon request, select 'No' for the first question and explain your exceptional situation in the text box.<br><br>Do the authors confirm that all data underlying the findings described in their manuscript are fully available without restriction?	
**Describe where the data may be found in full sentences. If you are copying our sample text, replace any instances of XXX with the appropriate details.**<br><br>• If the data are **held or will be held in a public repository**, include URLs, accession numbers or DOIs. If this information will only be available after acceptance, indicate this by ticking the box below. For example: *All XXX files are available from the XXX database (accession number(s) XXX, XXX.)*.<br>• If the data are all contained **within the manuscript and/or Supporting Information files**, enter the following: *All relevant data are within the manuscript and its Supporting Information files.*<br>• If neither of these applies but you are able to provide **details of access elsewhere**, with or without limitations, please do so. For example:<br><br>*Data cannot be shared publicly because of [XXX]. Data are available from the XXX Institutional Data Access / Ethics Committee (contact via XXX) for researchers who meet the criteria for access to confidential data.*<br><br>*The data underlying the results presented in the study are available from (include the name of the third party*	All data files are available from the<br>Harvard Dataverse at the KWTRP Research Data Repository<br>https://doi.org/10.7910/DVN/XMTCTW..

and contact information or URL).<br>• This text is appropriate if the data are owned by a third party and authors do not have permission to share the data.<br><br>* typeset	
Additional data availability information:	

Powered by Editorial Manager® and ProduXion Manager® from Aries Systems Corporation

Manuscript Click here to access/download;Manuscript;Wanjiku_P21012025_Manuscript

Induction of an early IFN-γ cellular response and high plasma levels of SDF-1α are inversely associated with COVID-19 severity and residence in rural areas in Kenyan patients

Short Title: Early IFN-γ response and high SDF-1α levels linked to Lower COVID-19 Severity in rural Kenyan patients

Authors

Perpetual Wanjiku [1]*, Benedict Orindi [1], John Kimotho [1], Shahin Sayed [2], Reena Shah [2], Mansoor Saleh [2], Jedidah Mwacharo [1], Christopher Maronga [3], Vivianne Olouch [2], Ann Karanu [2], Jasmit Shah [4], Zaitun Nneka [2], Lynette Isabella Ochola-Oyier [1 5], Abdirahman I. Abdi [1 5], Susanna Dunachie [6], Philip Bejon [1 5], Eunice Nduati [1 5], Francis M. Ndungu [1 5]*.

Affiliations

[1] Centre for Geographic Medicine Research (Coast), Kenya Medical Research Institute (KEMRI)-Wellcome Trust Research Programme, Kilifi, Kenya.

[2] Aga Khan University Hospital, 3rd Parklands Avenue, Nairobi, Kenya.

[3] Nuffield Department of Population Health, Cancer Epidemiology Unit, University of Oxford, Oxford, United Kingdom.

[4] Brain and Mind Institute and Department of Medicine, Aga Khan University, Nairobi, Kenya.

[5] Nuffield Department of Medicine, University of Oxford, Oxford, United Kingdom.

[6] Nuffield Department of Medicine, Centre for Global Health Research, University of Oxford, Oxford, United Kingdom.

* Corresponding authors

pwanjiku@kemri-wellcome.org (PW)

fndungu@kemri-wellcome.org (FMN)

Abstract

Introduction

COVID-19 was less severe in Sub-Saharan Africa (SSA) compared with Europe and North America. It is unclear whether these differences could be explained immunologically. Here we determined the levels of *ex vivo* SARS-CoV-2 peptide-specific IFN-γ producing cells, and levels of plasma cytokines and chemokines over the first month of COVID-19 diagnosis among Kenyan COVID-19 patients from urban and rural areas.

Methods

Between June 2020 and August 2022, we recruited and longitudinally monitored 188 COVID-19 patients from two regions in Kenya, Nairobi (urban, n = 152) and Kilifi (rural, n = 36), with varying levels of disease severity – severe, mild/moderate, and asymptomatic. IFN-γ secreting cells were enumerated at 0-, 7-, 14- and 28-days post diagnosis by an *ex vivo* enzyme-linked immunospot (ELISpot) assay following *in vitro* stimulation of peripheral blood mononuclear cells (PBMCs) with overlapping peptides from several SARS-CoV-2 proteins. A multiplexed binding assay was used to measure the levels of 22 plasma cytokines and chemokines.

Results

Higher frequencies of IFN-γ-secreting cells against the SARS-CoV-2 spike peptides were observed on the day of diagnosis among the asymptomatic compared to the patients with severe COVID-19. Higher concentrations of 17 of the 22 cytokines and chemokines measured were positively associated with severe disease, and in particular interleukin (IL)-8, IL-18 and IL-1ra ($p<0.0001$), while a lower concentration of SDF-1α was associated with severe disease ($p<0.0001$). Concentrations of 8 and 16 cytokines and chemokines including IL-18 were higher among Nairobi asymptomatic and mild patients compared to their

respective Kilifi counterparts. Conversely, the concentrations for SDF-1α were higher in rural Kilifi compared to Nairobi (p=0.012).

Conclusion

In Kenya, as seen elsewhere, pro-inflammatory cytokines and chemokines were associated with severe COVID-19, while an early IFN-γ cellular response to overlapping SARS-CoV-2 spike peptides was associated with reduced risk of disease. Living in urban Nairobi (compared with rural Kilifi) was associated with increased levels of pro-inflammatory cytokines/chemokines.

Keywords: COVID-19, SARS-CoV-2, IFN-γ-secreting cells, cytokines, chemokines, kinetics, Sub-Saharan Africa.

Introduction

Despite widespread transmission of SARS-CoV 2, SSA experienced a reduced burden of severe coronavirus disease 2019 (COVID-19) and associated mortality than North America and Europe (1). This observation is both puzzling (2) and paradoxical (3), because of the relatively weaker and underfunded health systems in SSA. Some of the proposed, but still unproven, explanations for the reduced burden of severe COVID-19 in SSA include under-diagnosis of cases and mortality, a younger population (7), warmer climatic conditions with outdoor living, high levels of pre-existing cross-protective antibodies and T-cells induced from a high prevalence of infectious agents with SARS-CoV-2 like immune determinants, and immune regulation associated with either prior BCG vaccination (5–11) or chronic/repeated infections including helminths (12) and malaria (13). Like other viruses, SARS-CoV-2 induces a plethora of inflammatory host responses, including cytokines and chemokines (14), that play key roles in either protective immunity or immunopathology (15). Notably, the pathogenesis of COVID-19 has been linked to dysregulated and excessive cytokine and chemokine responses, upon SARS-CoV-2 infection (16). Numerous studies have linked increased levels of cytokines and chemokines to severe COVID-19 and associated mortality, including IL-1β, IL-1ra, IL-2, IL-6, IL7, IL-8, IL-18, IFN-γ, TNF-α, IFN-γ-inducible protein 10 (IP-10), granulocyte macrophage-colony stimulating factor (GM-CSF), monocyte chemoattractant protein-1 (MCP-1), and Macrophage inflammatory protein-1 alpha (MIP-1-α) (17–24). Collectively called a “cytokine storm”, a dysregulated cytokine response is implicated as the cause of the multiple organ failures and the acute respiratory distress syndrome (ARDS), which characterise severed COVID-19 and associated fatalities (25).

Published data show that the characteristic antibody response to SARS-CoV-2 infection, where levels increase with time, viral loads and COVID-19 severity, were experienced in Kenyan patients (26). Thus, there is no evidence of the primary acute antibody response controlling the infection outcome. Aside from antibodies, T-cell responses can control viremia, either directly by killing virus infected cells or indirectly by providing the relevant co-stimulatory molecules for supporting antibody production by B cells (27). However, there is a paucity of data on the cellular response to SARS-CoV-2 in Kenyan patients, and its possible role in modulating disease severity. Furthermore, it is unclear whether the differences in the rates of severe disease between urban and rural dwellers within SSA, as well as between developed countries and SSA, could be explained immunologically. In this study, we collected longitudinal blood samples from individuals from Nairobi (urban) and Kilifi (rural) with varying degrees of COVID-19 severity (asymptomatic, mild/moderate and severe) and compared levels of their *ex vivo* SARS-CoV-2 Spike peptide-specific IFN-γ producing cells, and levels of plasma cytokines and chemokines over their first month of COVID-19 diagnosis.

Methods

Study design, setting and participants

Participant sampling has been described previously (26). Briefly, we included 400 blood samples from a longitudinal cohort study of 188 patients that aimed at understanding the kinetics of naturally acquired immune responses to SARS-CoV-2 among COVID-19 patients from two sites in Kenya: 1) The Aga Khan University Hospital (AKUH) in Nairobi, an urban metropolitan academic medical Centre; and 2) Kilifi County Hospital, a community-based government hospital serving a rural coastal region. The samples were collected during the COVID-19 pandemic between June 2020 and August 2022. At the time of the study, SARS-CoV-2 transmission in Nairobi was higher than in Kilifi (28). Participants were adults, aged ≥18 years old, recruited within seven days of positive diagnosis of COVID-19 by RT-PCR testing. Initial sampling was at day 0 (i.e., day of diagnosis). Follow-up and subsequent samplings were done on days 7, 14 and 28 from a positive SARS-CoV-2 infection diagnosis. We collected 20 mL of venous blood in sodium heparin vacutainer. Additionally, we included residual longitudinal plasma samples from the AKUH biobank for cytokine and chemokine measurements. These samples were collected from COVID-19 patients who consented to this follow-up study on the day of diagnosis (i.e., day 0) and on day 28.

COVID-19 severity classification

We included patients from five COVID-19 severity groups of asymptomatic, mild, moderate, severe, and critical as determined by clinicians at the time of diagnosis following the National Institutes of Health (NIH, USA) guidelines(29). Asymptomatic patients were those who tested positive for SARS-CoV-2 via RT-PCR but did not display any COVID-19 symptoms. Mild cases were SARS-CoV-2 positive and exhibited symptoms such as fever, sore throat, cough, malaise, headache, muscle pain, vomiting, nausea, diarrhoea, anosmia, or

ageusia, without any signs of shortness of breath, dyspnea, or abnormal chest imaging. Moderate cases tested positive for SARS-CoV-2 and showed evidence of lower respiratory tract infection based on clinical examination or imaging, with an oxygen saturation (SpO2) of ≥94% on room air. Severe cases were positive for SARS-CoV-2 with an SpO2 of <94% on room air, a ratio of arterial partial pressure of oxygen to fraction of inspired oxygen (PaO2/FiO2) <300 mm Hg, a respiratory rate >30 breaths/minute, and/or lung infiltrates >50%. Critically ill patients were positive for SARS-CoV-2 and experienced respiratory failure, septic shock, and/or multiple organ dysfunction syndrome. Due to the small numbers in the moderate and critical groups, mild cases were lumped together with moderate (mild/moderate), whilst critical were grouped with severe ones (severe). Thus, we studied immune responses among three COVID-19 patient groups: asymptomatic, mild/moderate and severe.

Procedures

Plasma separation and PBMC isolation

Plasma was separated by centrifuging the tubes at 440 g for 10 minutes using an Eppendorf 5810R centrifuge, aliquoted in 2 mL microcentrifuge tubes, and immediately stored in -80°C freezers until the time for laboratory analysis. PBMCs were isolated from the remaining blood component using density gradient centrifugation media (Lymphoprep™ (1.077 g/ml, Stem Cell Technologies), aliquoted to 1.8 mL cryovials, and stored in -196°C liquid nitrogen tanks until usage. Plasma and PBMC samples from AKUH were transported in dry ice and liquid nitrogen respectively, to the KEMRI–Wellcome Trust Research Programme laboratories in Kilifi and stored appropriately for laboratory analyses. Prior to use, PBMCs were thawed and rested at 37°C, 5% CO_2 for 15–16 hours. PBMC counting was done using Vi-CELL XR Cell Viability Analyser or Countess™ Cell Counting Chamber Slides (Thermo Scientific) before assay setup.

SARS-CoV-2 synthetic peptides pools for ELISpot measurements

A total of 641 peptides (15–18-mers with a ten amino acid overlap) were pooled into ten peptide pools, spanning different regions of the virus. The pools covered: the spike protein region (S1: positions 1–93 and S2: positions 94–178), membrane protein (M: positions 1–31), nucleocapsid protein (NP: positions 1–55), non-structural proteins (NSP 3B: positions 207–306, NSP 3C: position307–379, NSP 12B: positions 665–729 and NSP 15–16: positions 886–972) and Open Reading Frame (ORF3: positions 1–37 and ORF8: positions 1–15). These peptides (which were synthesised by Mimotopes Pty Ltd and a kind donation from Professor Susanna Dunachie's laboratory, Oxford University) were used to stimulate PBMCs for *ex vivo* IFN-γ ELISpot assay. The peptide sequences and pooling details are provided in Table S1.

Interferon gamma ELISpot assay

To quantify *the ex vivo* interferon gamma (IFN-γ) cellular response to overlapping SARS-CoV-2 peptides from different proteins, we stimulated PBMC with synthetic peptides pools through an *in vitro* IFN-γ ELISpot assay, as previously described (30). Briefly, 5 µg/ml of anti- human IFN-γ antibody clone 1-D1K (Mabtech, AB, Sweden) was used to coat Multiscreen-I 96 ELISpot plates overnight. Individual's PBMC were plated in duplicates at 200,000 cells per well for each specific protein, based on pre-prepared plate templates. Peptide pools were then added at a final concentration of 2 µg/mL per wells and incubated for 16–18 hours at 37^{0}C, 5% CO_2, 95% humidity. Concanavalin A (ConA; Sigma) was used as the positive control at a final concentration of 5 µg/mL per well, while dimethyl sulfoxide (DMSO; Sigma), which was a constituent of the diluent for the peptides and Con A was used at a similar concentration to peptides to serve as the negative control. IFN-γ secreting cells were then detected using an anti-human IFN-γ biotinylated antibody clone 7-B6-1 (Mabtech) at 1 µg/mL and an incubation for 2–4 hours. Thereafter, streptavidin alkaline phosphatase

antibody (Mabtech) was added at 1 µg/mL and incubated for 1–2 hours, and the IFN-γ spots then developed using 1-Step™ NBT/BCI (nitro blue tetrazolium/5-bromo-4-chloro-3-phosphatase) substrate (Thermo Scientific) during a 7-minute incubation in the dark. The enzyme-substrate reaction was stopped by rinsing the plate 3 times under running tap water. Plates were then airdried for at least 2 days on an open lab bench, and spots enumerated on an AID ELISpot Reader version 4.0. Results are hereby reported as spot-forming units (SFU)/10^6 PBMC after subtracting the background (mean SFU from negative control wells). Data from failed individual PBMC tests, defined here as either, an excessive background where the negative control wells had >80 SFU/10^6 PBMCs, or a positive control well with an average of <100 SFU/10^6 PBMCs (too few), were excluded. We also applied the ELISpot assay limit of detection of 10 SFU/10^6 PBMCs, with all wells having values <10 SFU/10^6 PBMCs replaced with 5 SFU/10^6 PBMCs. For the participants who did not have enough PBMC to be tested against all the peptide pools, we prioritised measurements against pools from S1, S2, NP and M proteins. Data are reported only for the individuals whose PBMC were tested against all the available peptide pools for each specific protein segment. We summed the responses from the different pools of the same protein segment, which resulted in different sample sizes for different proteins as follows: spike (171 samples for S1, S2), NP (162 samples), M (136 samples), NSPs (100 samples for NSP 3B, NSP 3C, NSP 12B and NSP 15 -16) and ORF (90 samples for ORF 3 and ORF 8) Table S2.

Luminex assay

Plasma concentrations of 22 cytokines and chemokines were measured using a Human ProcartaPlex™ Human Panel 1A (Thermo Fisher Scientific, Cat. No. EPX010-12010-901, Lot number 316776-000), which consisted of: a) Th1/Th2 specific cytokines: GM-CSF, IFN-γ, IL-1β, IL-2, IL-6, IL-8, IL-18, TNF-α, IL-9, IL-21; b) Pro-Inflammatory cytokines: IFN-α, IL-1α, IL-1ra, IL-7, TNF-β; and c) Chemokines: Eotaxin, GRO-α, IP-10, MCP-1, MIP-1α,

MIP-1β, SDF-1α, according to the manufacturer's instructions. Briefly,1x capture magnetic beads were added to the plates, and unbound beads were then washed away with 1X wash buffer. Plasma samples were thawed and diluted at 1:2 with 1X universal assay buffer (UAB) before addition to the plates. Standards from the kit at 4-fold serial dilutions of 1:5, 1:20, 1:80, 1:320, 1:1280, 1:5120, 1:20480 as well as a blank (UAB), were also added. The plates were then incubated on a shaker at 600 rpm for 2 hours at room temperature. After incubation, contents were discarded, plates washed, and 1X biotinylated detection antibody added. The plates were then incubated for 30 minutes on an Eppendorf Thermomixer Comfort shaker at room temperature. The plates were then washed, and 1X Streptavidin-PE added and incubated for 30 minutes on an Eppendorf Thermomixer Comfort shaker at room temperature, the plates were then washed before adding 1X reading buffer for 5 minutes on an Eppendorf Thermomixer Comfort shaker at room temperature. All wash steps were performed on an Invitrogen hand-held magnetic plate washer. Data were acquired on the Magpix systems multiplex Luminex machine and concentrations (pg/mL) of the samples calculated in Belysa® Immunoassay Curve Fitting software version 1.1.0 (31) using a 5- or 4-parameter logistic standard curve generated from standards of known concentration.

Statistical analysis

For the ELISpot data, time point specific geometric means (GMs) of IFN-γ secreting cells for each of the different SARS-COV-2 peptides pools were calculated for each severity group and geographical location. For each peptide, variations in ELISpot responses were compared using a linear mixed effects model on log-transformed PBMCs values with patient as a random effect, and time (i.e., day of sampling), severity group and time-by-severity group interaction term as fixed effects (32), followed by Tukey's multiple comparisons. Within each severity group, differences between geographic locations were compared using Kruskal

Wallis paired with a Dunn's multiple comparisons. This analysis set was restricted to 59 patients who had PBMC samples.

Cytokine and chemokine data were first normalised to have a zero mean and a standard deviation of one, and cross-correlations among the cytokines determined using Pearson correlations and principal component analysis (PCA). Principal components (PCs) were extracted based on scree-plot, variance explained and the interpretability of the components. Next, the non-normalised cytokine and chemokine data were log-transformed and fitted into linear mixed effects regression models with age, sex, day of sampling, location or disease severity as fixed effects and patient as a random effect. Interactions were explored and separate models fitted where necessary. For location comparisons, severe and moderate COVID-19 cases were excluded as these categories were only present in the Nairobi data. Results from the regression models were presented using heat maps, in which the effect size (i.e., coefficient) determined the density of colour-shading for each square. TNF-β data were excluded from the analyses as only one patient had a measurable concentration. Thus, we analysed 21 cytokines from all 188 patients. Analyses were performed using R version 4.3.0 (33). The factoextra package (34) was used for PCA. For visualisations, the pheatmap (35) and ggplot2 (36) packages and GraphPad Prism Software version 10.1.2 (37) were used.

Ethical approval

The study obtained ethical approval from the Kenya Medical Research Institute's Scientific and Ethics Review Unit (KEMRI SERU; protocol no. 4081) and the Aga Khan University, Nairobi, Institutional Ethics Review Committee (protocol no. 2020 IERC-55 V5 and 2020 IERC-135 V2). Written informed consent was obtained from all willing patients before their enrolment into the study.

Results

Participant baseline characteristics

Using the NIH clinical guidelines for grading COVID-19 severity of 188 patients, 27 (14%) were asymptomatic, 75 (40%) were mild/moderate and 86 (46%) were severe cases. Collectively, the 188 patients contributed 400 blood samples collected over the first month of diagnosis: day 0 (187 samples), day 7 (50 samples), day 14 (53 samples), and day 28 (110 samples). Fewer samples were collected on days 7 and 14 mainly due to design; that is, residual longitudinal plasma samples from AKUH biobank were only collected in day 0 and day 28. Of the 188 patients, 129 (69%) were male, 36 (19%) were from rural Kilifi and 152 (81%) from urban Nairobi (Table 1). Their median age at recruitment was 48 years (IQR 37–58), with disease severity increasing with age. All 86 patients with severe COVID-19 were from Nairobi as we were unable to recruit severe patients in Kilifi. Underlying comorbidities such as diabetes, HIV/AIDS, and hypertension were prevalent among the mild/moderate and severe COVID-19 groups. Two participants with severe disease died on day 7 and 28 respectively (Table 1).

Table 1. Participant demographic and clinical characteristics[†]

Characteristic	Asymptomatic (n = 27)	Mild/Moderate (n = 75)	Severe (n = 86)
Median age at recruitment (IQR), years	40 (33, 55)	46 (35, 54)	53 (43, 60)
Male sex	15 (56%)	46 (61%)	68 (79%)
Location			
Kilifi	21 (78%)	15 (20%)	0

Characteristic	Asymptomatic (n = 27)	Mild/Moderate (n = 75)	Severe (n = 86)
Nairobi	6 (22%)	60 (80%)	86 (100%)
Comorbidities			
Diabetic	1 (7%)	28 (42%)	47 (63%)
Hypertensive	0	15 (22%)	33 (44%)
HIV positive	0	5 (8%)	4 (5%)
Had COPD	0	0	1 (1%)
Had Renal disease	0	0	2 (3%)
Had heart disease	0	0	6 (8%)
Patient management			
Were hospital admissions	0	56 (84%)	75 (100%)
In intensive care unit	0	4 (6.0%)	15 (20%)
Clinical Outcome, died	0	0	2 (3%)

† Data are median (IQR) or number (%); COPD if Chronic obstructive pulmonary disease.

Kinetics and frequencies of *ex vivo* IFN-γ secreting cells by COVID-19 severity and geographical location

Kinetics and levels of IFN-γ secreting cell responses were assessed in a subset of 59 participants with PBMC samples, contributing 172 samples. At day 0, the frequency of IFN-γ secreting cells to overlapping SARS-CoV-2 spike peptide was significantly higher in asymptomatic patients compared to severe patients (GMs: 117 [95% CI 71–194] vs 32 [95% CI 8–76]; p=0.0366) (Figure 1a, Table S3). For the IFN-γ secreting cells specific to

overlapping M peptides, severe patients exhibited significantly higher levels at day 7 than mild/moderate patients (GMs: 90 [95% CI 37–217] vs 19 [95% CI 10–39]; p= 0.0180; Figure 1b, Table S3). We did not observe any significant differences in the frequencies of IFN-γ secreting cell responses to NP, NSPs, and ORFs overlapping peptides (Figure 1c–e, Table S3). For all five SARS-CoV-2 peptides we observed temporal differences in IFN-γ secreting cells within each COVID-19 severity group (Figure 1, Table S3). Spike peptide: In the asymptomatic group, IFN-γ secreting cells were significantly elevated on day 7 than on day 14 (GMs: 161 [95% CI 86–301] vs 92 [95% CI 44–189]; p=0.0039) and day 28 (102 [95% CI 49–209]; p=0.0042). For the mild/moderate group, levels significantly increased from 86 (95% CI 53–138) on day 7 to 142 (95% CI 78–257) on day 14 (p=0.0403). In the severe group, significantly higher levels were observed on day 7 (70 [95% CI 29–165]; p=0.0381), day 14 (117 [95% CI 52–260]; p=0.0058), and day 28 (108 [95% CI 33–348]; p=0.0037) relative to day 0 (23 95% CI 7–76).

M Peptide: In the asymptomatic patients, IFN-γ secreting cells were significantly higher on day 14 (89 [95% CI 30–258]) compared to day 0 (GMs: 28 [95% CI 13–59]; p=0.0364) and day 7 (GMs: 48 [95% CI 18–129]; p=0.0297). In the mild/moderate group, day 14 levels were significantly higher compared to day 0 (p=0.0039), day 7 (p<0.0001), and day 28 (p=0.002). Additionally, day 28 levels were significantly higher than day 7 (p=0.0049). No significant differences were observed between timepoints in the severe group.

NP Peptide: Among the asymptomatic group, a significant decline was observed from day 7 (71 [95% CI 33–153]) to day 28 (47 [95% CI 24–88]; p=0.0035). In the mild/moderate group, IFN-γ secreting cell levels were significantly higher on day 14 (GMs: 67 [95% CI 34–130]) compared to day 0 (GMs: 35 [95% CI 14–83]; p=0.0037), day 7 (GMs: 44 [95% CI 26–73]; p=0.0178), and day 28 (GMs: 47 [95% CI 24–91]; p=0.0003). No significant differences were observed between timepoints in the severe group. For NSP peptide: a significant decline

in IFN-γ secreting cells was observed within the mild/moderate group from 80 (95% CI 33–194) at day 14 to 28 (95% CI 7–106) at day 28 (p=0.0316). For ORF peptide: a significant increase in IFN-γ secreting cells was observed in the mild/moderate group between day 0 (14 [95% CI 4–43]) and day 7 (GMs: 38 [95% CI 16–85]; p=0.0242).

Figure 1. Induction of a higher IFN-gamma cellular response to Spike protein on day of diagnosis is associated with asymptomatic infection.

Frequencies of ex-vivo IFN-γ secreting cells against SARS-CoV-2 peptide pools spanning (**a)** spike protein, (**b)** M protein, **c** NP protein, **(d)** NSP proteins and (**e)** ORF proteins. Bars represent geometric mean and 95% CI.

Linear mixed effects model with Tukey's multiple comparisons, was used, * $P < 0.05$.

Number of samples analysed for: spike responses = 171, M responses = 136, NP responses = 162, NSP responses = 100 and ORF responses = 90.

We also assessed whether there were differences in IFN-γ secreting cell responses by location (urban Nairobi and rural Kilifi) among asymptomatic and mild patients. Relative to Nairobi, significantly higher levels of IFN-γ secreting cell responses to the SARS-CoV-2 spike peptide (218 [95% CI 125–381] vs 56 [95% CI 28–110]; p=0.0057; Figure 2a) and NP peptide (94 [95% CI 51–169] vs 19 [95% CI 8–48; p=0.0171; Figure 2c) were observed among the asymptomatic patients in Kilifi on day 0. There were no significant differences between Kilifi and Nairobi in the IFN-γ secreting cell responses to overlapping peptides for the M protein for the asymptomatic patients (Figure 2b), nor for the spike, or NP, and or M peptides among mild patients (Figure 2d–f). Data for severe patients are shown for Nairobi participants only (Figure 2g–i), as we were unable to recruit severe patients in Kilifi.

Figure 2. Induction of a higher IFN-γ cellular response on the day of diagnosis is associated with Kilifi participants.

Comparison of IFN-γ cellular responses between Kilifi and Nairobi COVID-19 patients with: asymptomatic disease for (**a**) Spike protein, (**b**) M protein, (**c**) NP protein; Mild disease for (**d**) Spike protein, € M protein, (**f**) NP protein; and Severe disease for (**g**) Spike protein, (**h**) M protein, (**i**) NP protein. Kilifi didn't have severe patients.

Bars represent geometric mean and 95% CI. Kruskal–Wallis one-way ANOVA, with Dunn's multiple comparisons test, was performed. * $P < 0.05$, ** $P < 0.01$.

Kinetics of cytokine and chemokine responses across COVID-19 severity groups and geographical location

Asymptomatic patients consistently showed elevated levels of SDF-1α at all time-points, but lower levels of all the other cytokines and chemokines measured and no detectable levels of IL-9 (Figure 3a). Similarly, for mild/moderate patients, high levels of SDF-1α (Figure 3b), were observed at all timepoints whereas other cytokines and chemokines were secreted at low to intermediate levels. High cytokine and chemokines levels were seen among the severe patients with IL-Iβ, IL-6, IL-2, IFN-γ, GM-CSF, IFN-α, IL-7 and GRO-α decreasing over time, while MIP-1α, MIP-Iβ, MCP-1, and SDF-1α increased with time. Levels for IL1-ra, and IL-9 were similar at all the time points (Figure 3c).

Figure 3. Kinetics of Cytokine and Chemokine Concentrations in COVID-19 Patients. Mean of log10-transformed cytokine/chemokine concentrations plotted over time for **(a)** Asymptomatic participants, (**b**) Mild/Moderate participants, and (**c**) Severe participants**.** * - Levels for all participants were below detectable levels.

We compared asymptomatic against mild/moderate and severe participants to evaluate differences among the clinical phenotypes. Mild/moderate participants had significantly higher levels of IL-18, IL-8, IL-1ra, IL-6, GM-CSF, IP-10, MCP-1, TNF-α, MIP-1α, IFN-γ,

IL-2, IL-7, IL-1β, IL-9, Eotaxin and IFN-α than asymptomatic patients. The largest effect sizes were observed with IL-18 (1.107, p<0.0001), IL-8 (1.105, p<0.0001) and IL-1ra, (0.672, p=0.013). On the contrary, levels for SDF-1α were significantly reduced among the mild/moderate patients relative to the asymptomatic (effect size -0.182, p<0.0001) (Figure 4). Severe participants had significantly higher levels for cytokines (IL-8, IL-18, IL-1ra, IL-6, IP-10, MIP-1α, TNF-α, IL-9, IFN-γ, GM-CSF, IL-7, IL-1β, MCP-1, IL-2, GRO-α, Eotaxin and IFN-α) compared to asymptomatic cases, except for IL-1a, IL-21 and MIP-Iβ which had similar levels, and SDF-1α (effect size -0.195, p<0.0001), which was significantly reduced. The largest effect size was observed in IL-8 (1.754, p<0.0001), IL-18 (1.666, p<0.0001) and IL-1ra (1.197, p<0.0001) (Figure 4).

For asymptomatic individuals, the cytokines and chemokine levels for IL-6, MIP-1α, IL-18, GRO-α, IL-2, IL-8, TNF-α and GM-CSF were significantly higher among Nairobi than Kilifi patients. Comparisons for IL-6 (1.139, p<0.0001), MIP-1a (1.093, p=0.004) and IL-18 (1.025, p=0.002) had the largest effect sizes (Figure 4). For mild patients, all cytokines (IL-8, IL-18, IL-1ra, MIP-1α, IL-6, IP-10, IFN-γ, TNF-α, MCP-1, GM-CSF, IL-9, IL-1β, Eotaxin, IL-7, MIP-Iβ, and IFN-α) were significantly higher among Nairobi patients in comparison to Kilifi patients except for GRO-α, IL-1α, IL-2 and IL-21 which were similar. Notably, the largest effect size for these comparisons was observed for IL-8 (1.69, p<0.0001), IL-18 (1.634, p<0.0001) and IL-1ra (1.355, p<0.001). On the other hand, SDF-1α (effect size -0.161, p = 0.017) was significantly lower in Nairobi than in Kilifi patients.

Figure 4. Higher Cytokine and Chemokine concentrations in Asymptomatic and Mild Patients from Nairobi Compared to Kilifi, and in Mild/Moderate and Severe Patients Compared to Asymptomatic Individuals.

ns – not-significant, *p* value >0.05, empty means it was significant at p<0.05 - <0.0001. * - Levels for all participants were below detectable levels.

Principal component analysis

The concentrations of the majority of the 21 cytokines and chemokines were positively correlated with each other. However, the levels of SDF-1α were negatively correlated with those of IL-2, IP-10, IL-7, IFN-γ, GM-CSF, and IL-18 (Figure S1). We retained the first three principal components from a principal component analysis, accounting for 62% of the total variability in the 21 cytokines and chemokines (Figure S2a). IL-9, MIP-1α, TNF-α, MCP-1, MIP-Iβ, IL1-ra, IL-6, IL-Iβ, GRO-α and IL-8 were the most strongly associated with the first PC. The second PC was most strongly associated with IP-10, IFN-γ, GM-CSF, IL-2, IL-18, IL-7, IFN-α, Eotaxin and SDF-1α. The third PC was associated with IL-1 α and IL-21 (Figure S3). There was no clear separation among the mild/moderate and severe groups. However, data points for the asymptomatic participants clustered together demonstrating a much-reduced diversity of cytokine and chemokine levels than the other groups (Figure 5), and median PC scores did not change over the first month (Figure S2b).

Figure 5. Scatter plot by Severity at baseline.

(a) PC1 vs PC2; **(b)** PC2 vs PC3

Among the symptomatic groups (mild/moderate/severe), a steady decline of all the PCs over one month was observed, with a steep decline occurring between day 0 and day 7 (Figure S2c). There was no apparent difference of the cytokine and chemokine responses between asymptomatic participants from Kilifi and Nairobi (Figure S4a). For mild cases, Kilifi patients clustered together, although there was a slight overlap with Nairobi participants (Figure S4b). Using biplots, there was no apparent difference between asymptomatic and mild Kilifi patients (Figure S4c). For Nairobi, clinical phenotypes were asymptomatic, mild, moderate, and severe. We combined the mild and moderate, and observed no clear difference

between the mild/moderate and severe groups, while the few asymptomatic participants seemed to cluster together (Figure S4d).

Discussion and conclusion

In this study, we measured associations between the acute IFN-γ cellular, and 22 cytokine and chemokine, immune response to SARS-CoV-2 (within a month of diagnosis) with COVID-19 severity and possible modulation by differential environmental exposures in urban and rural areas in Kenya.

As had been seen in earlier reports from Singapore, Netherlands, and Italy, which associated increased IFN-γ secreting cells measured by ELISpot assay in the early phase of infection with milder disease (38–40), we found that higher frequencies of an early IFN-γ cellular response to SARS-CoV-2 spike overlapping peptides was associated with asymptomatic, compared to severe SARS-CoV-2 infection outcomes. This observation would suggest either: 1) that the IFN-γ cellular response contribute to protection against disease progression, or 2) that developing severe COVID-19 depresses this response. The latter interpretation may be supported by previous reports from China and USA reporting correlations of an early induction of increased basal T-cell specific responses ($CD4^+$ and $CD8^+$ T) in COVID-19 patients with mild disease, which was suppressed among their severely sick counterparts (41,42).

Whilst the concentrations of the pro-inflammatory cytokines and chemokines IL-6, IL-Iβ, GRO-α, IFN-γ, GM-CSF, IFN-α, IL-7 and IL-2 in severe patients declined to basal levels at day 28 from day of diagnosis, we found that the concentrations for the chemokines MIP-1α, MIP-Iβ, MCP-1, and SDF-1α were increasing with time. This is to be expected as cytokines and chemokines play different roles and at different times of the immune response to infection. MIP-1α, MCP-1 and MIP-Iβ are chemoattractant and play key roles in the recruitments of leukocytes such as monocytes, T-cells, and neutrophil to the sites of infection (43,44). Similarly increasing kinetics for MIP-1α and MCP-1 in severe and fatal patients were reported in Norway and China, respectively (45,46). Notably, we found that higher

concentrations of SDF-1α were associated with asymptomatic individuals, hinting at a potential protective role from severe disease progression. SDF-1α (CXCL12), is a chemokine involved in the recruitment of T-cells (47), CD34+ hematopoietic stem/progenitor cells (48), lymphocytes and monocytes (49) to the site of infection further enhancing inflammation. In contrast, studies from China and Bulgaria did not observe significant differences in SDF-1α levels among asymptomatic, mild, moderate, severe or fatal cases (18,46,50). However, others have also implicated SDF-1α in disease severity based on genetic association studies in a single-centre study (51), although this finding was not corroborated in multi-centre genome-wide association studies (52). SDF-1α may aid in the timely recruitment of T and other cells (47), to the sites of infection, enhancing viral elimination, reduction in inflammation, and promotion of recovery.

We demonstrate that elevated levels of eighteen cytokines and chemokines were associated with severe COVID-19, in agreement with previous reports linking them to severe lung injury and ARDS (17–22,53). However, the associations with IL-8, IL-18 and IL-1ra, had the strongest effect sizes in the current study. IL-8, has been implicated in the activation and recruitment of neutrophils to the site of infection, thereby promoting inflammation (54). IL-18 amplifies the immune response by inducing the production of IFN-γ by T-cells and natural killer cells (55). Thus, IL-8 and IL-18 could amplify the excessive inflammation that characterises severe COVID-19. On the contrary, IL1-ra is known to suppress the production of proinflammatory cytokines such as IL-1 and TNF-α (56), and probably helps mitigate the effects of excessive inflammation, thus reducing tissue damage and associated mortality.

In parallel to the reduced rates of severe COVID-19 and associated deaths in SSA relative to North America and Europe, we and others have suggested higher rates of severe COVID-19 in cities compared to rural areas in SSA (28). Whilst this difference could be explained by higher levels of SARS-CoV-2 transmission in busy metropolitan cities, or by more complete

reporting of cases (28,57,58), we wondered whether there were also plausible biological explanations. We compared inflammatory cytokine and chemokine levels, and found that the asymptomatic patients from Nairobi had higher levels of 8 cytokines and chemokines than their asymptomatic counterparts from Kilifi, with IL-6, IL-18 and MIP-1α, having the strongest associations. Similarly, levels of 16 cytokines and chemokines were higher among mild- Nairobi, than Kilifi, COVID-19 patients with IL-8, IL-18 and IL-1ra being the most differentially secreted. Moreover, the basal frequencies of IFN-γ secreting cells in asymptomatic patients from Nairobi, relative to those of their Kilifi counterparts, were reduced. Collectively, these findings would suggest that the immune response to SARS-CoV-2 is less inflammatory among residents of rural areas (59).

Our study was faced with a few limitations. Firstly, our data are incomplete for some of the patients due to missed follow-ups, or due to unavailability of adequate numbers of PBMCs to quantify IFN-γ cellular responses to the full spectrum of the peptide pools corresponding to all the different SARS-CoV-2 proteins. However, our analyses are assumed valid under the missing at random mechanism given the likelihood approach (60). Secondly, we had difficulties recruiting asymptomatic patients in Nairobi and were unable to recruit severe cases in Kilifi and thus the sample size is relatively small.

In conclusion, although severe disease was rare in Kenya, the inflammatory cytokine profile in patients with severe COVID is similar to that of North American and European severe COVID-19 patients. However, just like the early IFN-γ secreting cellular response, increased levels of the chemokine, SDF-1α, were associated with asymptomatic SARS-CoV-2 infection, suggesting a potential role of these responses in protection against disease progression. Finally, the differential cytokine and chemokine, and IFN-γ cellular responses between urban Nairobi and rural Kilifi patients suggest a plausible biological explanation for the increased frequency of severe COVID-19 in African SSA cities relative to rural areas.

Together, these findings provide insights into potentially COVID-19 protective immune responses, and their modulation by differential environmental exposures. Nonetheless, additional studies are required to extend and replicate these important findings as they could inform future control of COVID-19 and empower the control of similar pandemics.

Acknowledgments

We thank the field workers, laboratory staff and healthcare workers involved in the longitudinal blood sampling. We appreciate all the study participants.

References

1. Cabore JW, Karamagi HC, Kipruto HK, Mungatu JK, Asamani JA, Droti B, et al. COVID-19 in the 47 countries of the WHO African region: a modelling analysis of past trends and future patterns. Lancet Glob Health. 2022 Aug;10(8):e1099–114.

2. Maeda JM, Nkengasong JN. The puzzle of the COVID-19 pandemic in Africa. Science. 2021 Jan;371(6524):27–8.

3. Osayomi T, Adeleke R, Akpoterai LE, Fatayo OC, Ayanda JT, Moyin-Jesu J, et al. A Geographical Analysis of the African COVID-19 Paradox: Putting the Poverty-as-a-Vaccine Hypothesis to the Test. Earth Syst Environ. 2021 Sep 1;5(3):799–810.

4. Diop BZ, Ngom M, Pougué Biyong C, Pougué Biyong JN. The relatively young and rural population may limit the spread and severity of COVID-19 in Africa: a modelling study. BMJ Glob Health. 2020 May;5(5):e002699.

5. Wamai RG, Hirsch JL, Van Damme W, Alnwick D, Bailey RC, Hodgins S, et al. What Could Explain the Lower COVID-19 Burden in Africa despite Considerable Circulation of the SARS-CoV-2 Virus? Int J Environ Res Public Health. 2021 Aug 16;18(16):8638.

6. Ashworth J, Mathie D, Scott F, Mahendran Y, Woolhouse M, Stoevesandt O, et al. Peptide microarray IgM and IgG screening of pre-SARS-CoV-2 human serum samples from Zimbabwe for reactivity with peptides from all seven human coronaviruses: a cross-sectional study. Lancet Microbe. 2023 Apr 1;4(4):e215–27.

7. Braun J, Loyal L, Frentsch M, Wendisch D, Georg P, Kurth F, et al. SARS-CoV-2-reactive T cells in healthy donors and patients with COVID-19. Nature. 2020 Nov 12;587(7833):270–4.

8. Le Bert N, Tan AT, Kunasegaran K, Tham CYL, Hafezi M, Chia A, et al. SARS-CoV-2-specific T cell immunity in cases of COVID-19 and SARS, and uninfected controls. Nature. 2020 Aug;584(7821):457–62.

9. Grifoni A, Weiskopf D, Ramirez SI, Mateus J, Dan JM, Moderbacher CR, et al. Targets of T Cell Responses to SARS-CoV-2 Coronavirus in Humans with COVID-19 Disease and Unexposed Individuals. Cell. 2020 Jun 25;181(7):1489-1501.e15.

10. Weiskopf D, Schmitz KS, Raadsen MP, Grifoni A, Okba NMA, Endeman H, et al. Phenotype and kinetics of SARS-CoV-2–specific T cells in COVID-19 patients with acute respiratory distress syndrome. Sci Immunol. 2020 Jun 26;5(48):eabd2071.

11. Mateus J, Grifoni A, Tarke A, Sidney J, Ramirez SI, Dan JM, et al. Selective and cross-reactive SARS-CoV-2 T cell epitopes in unexposed humans. Science. 2020 Oct 2;370(6512):89–94.

12. Naidoo P, Ghazi T, Chuturgoon AA, Naidoo RN, Ramsuran V, Mpaka-Mbatha MN, et al. SARS-CoV-2 and helminth co-infections, and environmental pollution exposure: An epidemiological and immunological perspective. Environ Int. 2021 Nov 1;156:106695.

13. Anyanwu MU. The association between malaria prevalence and COVID-19 mortality. BMC Infect Dis. 2021 Sep 19;21(1):975.

14. Mogensen TH, Paludan SR. Virus-cell interactions: impact on cytokine production, immune evasion and tumor growth. Eur Cytokine Netw. 2001;12(3):382–90.

15. Mogensen TH, Paludan SR. Molecular Pathways in Virus-Induced Cytokine Production. Microbiol Mol Biol Rev. 2001 Mar;65(1):131.

16. Ye Q, Wang B, Mao J. The pathogenesis and treatment of the `Cytokine Storm’ in COVID-19. J Infect. 2020 Jun;80(6):607–13.

17. Huang C, Wang Y, Li X, Ren L, Zhao J, Hu Y, et al. Clinical features of patients infected with 2019 novel coronavirus in Wuhan, China. The Lancet. 2020 Feb 15;395(10223):497–506.

18. Chi Y, Ge Y, Wu B, Zhang W, Wu T, Wen T, et al. Serum Cytokine and Chemokine Profile in Relation to the Severity of Coronavirus Disease 2019 in China. J Infect Dis. 2020 Aug 4;222(5):746–54.

19. Qin C, Zhou L, Hu Z, Zhang S, Yang S, Tao Y, et al. Dysregulation of Immune Response in Patients With Coronavirus 2019 (COVID-19) in Wuhan, China. Clin Infect Dis Off Publ Infect Dis Soc Am. 2020 Jul 28;71(15):762–8.

20. Trifonova I, Ngoc K, Nikolova M, Emilova R, Todorova Y, Gladnishka T, et al. Patterns of cytokine and chemokine expression in peripheral blood of patients with COVID-19 associated with disease severity. Int J Immunopathol Pharmacol. 2023 Dec;37:039463202311636.

21. Ndoricyimpaye EL, Van Snick J, Robert R, Bikorimana E, Majyambere O, Mukantwari E, et al. Cytokine Kinetics during Progression of COVID-19 in Rwanda Patients: Could IL-9/IFNγ Ratio Predict Disease Severity? Int J Mol Sci. 2023 Jul 31;24(15):12272.

22. Ling L, Chen Z, Lui G, Wong CK, Wong WT, Ng RWY, et al. Longitudinal Cytokine Profile in Patients With Mild to Critical COVID-19. Front Immunol [Internet]. 2021 Dec 6 [cited 2024 Apr 19];12. Available from:

https://www.frontiersin.org/journals/immunology/articles/10.3389/fimmu.2021.763292/full

23. Liu J, Li S, Liu J, Liang B, Wang X, Wang H, et al. Longitudinal characteristics of lymphocyte responses and cytokine profiles in the peripheral blood of SARS-CoV-2 infected patients. eBioMedicine [Internet]. 2020 May 1 [cited 2024 Jul 18];55. Available from: https://www.thelancet.com/journals/ebiom/article/PIIS2352-3964(20)30138-9/fulltext

24. Thwaites RS, Sanchez Sevilla Uruchurtu A, Siggins MK, Liew F, Russell CD, Moore SC, et al. Inflammatory profiles across the spectrum of disease reveal a distinct role for GM-CSF in severe COVID-19. Sci Immunol. 2021 Mar 10;6(57):eabg9873.

25. Darif D, Hammi I, Kihel A, Saik IEI, Guessous F, Akarid K. The pro-inflammatory cytokines in COVID-19 pathogenesis: What goes wrong? Microb Pathog. 2021 Apr;153:104799.

26. Kimotho J, Sein Y, Sayed S, Shah R, Mwai K, Saleh M, et al. Kinetics of naturally induced binding and neutralising anti-SARS-CoV-2 antibody levels and potencies among SARS-CoV-2 infected Kenyans with diverse grades of COVID-19 severity: an observational study. Wellcome Open Res. 2023 Aug 17;8:350.

27. Sette A, Crotty S. Adaptive immunity to SARS-CoV-2 and COVID-19. Cell. 2021 Feb;184(4):861–80.

28. Brand SPC, Ojal J, Aziza R, Were V, Okiro EA, Kombe IK, et al. COVID-19 transmission dynamics underlying epidemic waves in Kenya. Science. 2021 Nov 19;374(6570):989–94.

29. NIH. COVID-19 Treatment Guidelines. 2021 [cited 2024 Jun 13]. Clinical Spectrum of SARS-CoV-2 Infection. Available from: https://www.covid19treatmentguidelines.nih.gov/overview/clinical-spectrum/

30. Angyal A, Longet S, Moore SC, Payne RP, Harding A, Tipton T, et al. T-cell and antibody responses to first BNT162b2 vaccine dose in previously infected and SARS-CoV-2-naive UK health-care workers: a multicentre prospective cohort study. Lancet Microbe. 2022 Jan;3(1):e21–31.

31. Merck KGaA. Sigmaaldrich Belysa® Immunoassay Curve Fitting Software, Darmstadt, Germany. 2022 [cited 2024 Apr 11]. Belysa® Immunoassay Curve Fitting Software. Available from: https://www.sigmaaldrich.com/KE/en/services/software-and-digital-platforms/belysa-immunoassay-curve-fitting-software

32. Gelman A, Hill J. Higher Education from Cambridge University Press. Cambridge University Press; 2006 [cited 2024 Oct 28]. Data Analysis Using Regression and Multilevel/Hierarchical Models. Available from: https://www.cambridge.org/highereducation/books/data-analysis-using-regression-and-multilevel-hierarchical-models/32A29531C7FD730C3A68951A17C9D983

33. R Core Team. R Foundation for Statistical Computing, Vienna, Austria. 2023 [cited 2024 Mar 8]. R: A language and environment for statistical computing. Available from: https://www.R-project.org/

34. Kassambara A, Mundt F. factoextra: Extract and Visualize the Results of Multivariate Data Analyses [Internet]. 2020 [cited 2024 Mar 8]. Available from: https://CRAN.R-project.org/package=factoextra

35. Kolde R. pheatmap: Pretty Heatmaps [Internet]. 2019 [cited 2024 Mar 8]. (R package version 1.0.12). Available from: https://cran.r-project.org/web/packages/pheatmap/index.html

36. Wickham H. ggplot2: Elegant Graphics for Data Analysis [Internet]. Cham: Springer International Publishing, New York; 2016 [cited 2024 Mar 8]. (Use R!). Available from: http://link.springer.com/10.1007/978-3-319-24277-4

37. Graph pad prism programming team. Graph pad Prism, Boston, Massachusetts, USA. 2023 [cited 2024 Apr 11]. Prism - GraphPad. Available from: https://www.graphpad.com/features

38. Tan AT, Linster M, Tan CW, Bert NL, Chia WN, Kunasegaran K, et al. Early induction of functional SARS-CoV-2-specific T cells associates with rapid viral clearance and mild disease in COVID-19 patients. Cell Rep [Internet]. 2021 Feb 9 [cited 2024 Aug 3];34(6). Available from: https://www.cell.com/cell-reports/abstract/S2211-1247(21)00041-3

39. Rümke LW, Smit WL, Bossink A, Limonard GJM, Muilwijk D, Haas LEM, et al. Impaired SARS-CoV-2 specific T-cell response in patients with severe COVID-19. Front Immunol [Internet]. 2023 Apr 17 [cited 2024 Sep 13];14. Available from: https://www.frontiersin.org/journals/immunology/articles/10.3389/fimmu.2023.1046639/full

40. Garofalo E, Biamonte F, Palmieri C, Battaglia AM, Sacco A, Biamonte E, et al. Severe and mild-moderate SARS-CoV-2 vaccinated patients show different frequencies of IFNγ-releasing cells: An exploratory study. PLOS ONE. 2023 Feb 9;18(2):e0281444.

41. Zeng Q, Li YZ, Dong SY, Chen ZT, Gao XY, Zhang H, et al. Dynamic SARS-CoV-2-Specific Immunity in Critically Ill Patients With Hypertension. Front Immunol [Internet]. 2020 Dec 10 [cited 2024 Sep 13];11. Available from: https://www.frontiersin.org/journals/immunology/articles/10.3389/fimmu.2020.596684/full

42. Neidleman J, Luo X, George AF, McGregor M, Yang J, Yun C, et al. Distinctive features of SARS-CoV-2-specific T cells predict recovery from severe COVID-19. Cell Rep [Internet]. 2021 Jul 20 [cited 2024 Sep 13];36(3). Available from: https://www.cell.com/cell-reports/abstract/S2211-1247(21)00827-5

43. Takahashi M, Galligan C, Tessarollo L, Yoshimura T. Monocyte Chemoattractant Protein-1 (MCP-1), Not MCP-3, Is the Primary Chemokine Required for Monocyte Recruitment in Mouse Peritonitis Induced with Thioglycollate or Zymosan A. J Immunol Baltim Md 1950. 2009 Sep 1;183(5):3463–71.

44. Taub DD, Conlon K, Lloyd AR, Oppenheim JJ, Kelvin DJ. Preferential Migration of Activated CD4+ and CD8+ T Cells in Response to MIP-1α and MIP-1β. Science. 1993 Apr 16;260(5106):355–8.

45. Jøntvedt Jørgensen M, Holter JC, Christensen EE, Schjalm C, Tonby K, Pischke SE, et al. Increased interleukin-6 and macrophage chemoattractant protein-1 are associated with respiratory failure in COVID-19. Sci Rep. 2020 Dec 10;10:21697.

46. Xu ZS, Shu T, Kang L, Wu D, Zhou X, Liao BW, et al. Temporal profiling of plasma cytokines, chemokines and growth factors from mild, severe and fatal COVID-19 patients. Signal Transduct Target Ther. 2020 Jun 19;5(1):1–3.

47. Nanki T, Lipsky PE. Cutting Edge: Stromal Cell-Derived Factor-1 Is a Costimulator for CD4+ T Cell Activation. J Immunol. 2000 May 15;164(10):5010–4.

48. Aiuti A, Webb IJ, Bleul C, Springer T, Gutierrez-Ramos JC. The Chemokine SDF-1 Is a Chemoattractant for Human CD34Ƨ Hematopoietic Progenitor Cells and Provides a New Mechanism to Explain the Mobilization of CD34Ƨ Progenitors to Peripheral Blood.

49. Bleul CC, Fuhlbrigge RC, Casasnovas JM, Aiuti A, Springer TA. A highly efficacious lymphocyte chemoattractant, stromal cell-derived factor 1 (SDF-1). J Exp Med. 1996 Sep 1;184(3):1101–9.

50. Trifonova I, Ngoc K, Nikolova M, Emilova R, Todorova Y, Gladnishka T, et al. Patterns of cytokine and chemokine expression in peripheral blood of patients with COVID-19 associated with disease severity. Int J Immunopathol Pharmacol. 2023 Dec;37:039463202311636.

51. Korayem OH, Ahmed AE, Meabed MH, Magdy DM, Abdelghany WM. Genetic clues to COVID-19 severity: exploring the stromal cell-derived factor-1/CXCL12 rs2839693 polymorphism in adult Egyptians. BMC Infect Dis. 2023 Oct 19;23:702.

52. Pairo-Castineira E, Rawlik K, Bretherick AD, Qi T, Wu Y, Nassiri I, et al. GWAS and meta-analysis identifies 49 genetic variants underlying critical COVID-19. Nature. 2023 May;617(7962):764–8.

53. Zhang Z, Ai G, Chen L, Liu S, Gong C, Zhu X, et al. Associations of immunological features with COVID-19 severity: a systematic review and meta-analysis. BMC Infect Dis. 2021 Aug 3;21(1):738.

54. Harada A, Sekido N, Akahoshi T, Wada T, Mukaida N, Matsushima K. Essential involvement of interleukin-8 (IL-8) in acute inflammation. J Leukoc Biol. 1994;56(5):559–64.

55. Dinarello C, Novick D, Kim S, Kaplanski G. Interleukin-18 and IL-18 Binding Protein. Front Immunol [Internet]. 2013 Oct 8 [cited 2024 Aug 21];4. Available from: https://www.frontiersin.org/journals/immunology/articles/10.3389/fimmu.2013.00289/full

56. Dayer JM. Evidence for the biological modulation of IL-1 activity: The role of IL-1Ra. Clin Exp Rheumatol. 2001 Nov 30;20:S14-20.

57. Kleynhans J, Tempia S, Wolter N, von Gottberg A, Bhiman JN, Buys A, et al. SARS-CoV-2 Seroprevalence in a Rural and Urban Household Cohort during First and Second Waves of Infections, South Africa, July 2020–March 2021. Emerg Infect Dis. 2021 Dec;27(12):3020–9.

58. Abdella S, Riou S, Tessema M, Assefa A, Seifu A, Blachman A, et al. Prevalence of SARS-CoV-2 in urban and rural Ethiopia: Randomized household serosurveys reveal level of spread during the first wave of the pandemic. EClinicalMedicine. 2021 May 1;35:100880.

59. Samandari T, Ongalo JB, McCarthy KD, Biegon RK, Madiega PA, Mithika A, et al. Prevalence and functional profile of SARS-CoV-2 T cells in asymptomatic Kenyan adults. J Clin Invest. 133(13):e170011.

60. Little RJA, Rubin DB. Statistical Analysis with Missing Data. John Wiley & Sons; 2019. 462 p.

Supporting information

Figure S1. Correlation matrix of 21 cytokines across all time points from 400 patients. Pearson's correlation coefficients are visualised, with red indicating positive correlations, blue negative correlations, and white representing no correlation.

Figure S2. Principal component analysis of the cytokines. a) Scree-plot showing that the first 3 principal components explained 62% of variability in the cytokines data for all participants; **(b)** Line plots for the first 3 principal component illustrating trends over time for asymptomatic participants; and **(c)** Line plots for the first 3 principal component illustrating trends over time for symptomatic (mild, moderate and severe) participants.

Figure S3. Cytokines loading on the first three principal components (PC1–PC3). The color scale indicates the loading value, with red indicating a higher positive loading, blue a higher negative loading and light-yellow minimal loading.

Figure S4. Scatter plots showing cytokine data for participants grouped by location and clinical phenotype. (a) Asymptomatic participants, **(b)** mild cases, **(c)** participants from Kilifi, and **(d)** participants from Nairobi. Each point represents an individual's cytokine measurement, allowing for a visual assessment of cytokine variability across different groups based on location and clinical presentation.

Table S1. Peptides Sequences. Each entry represents a distinct peptide along with its associated properties. The pooling strategy for the 10 peptide pools used is shown below.

Table S2. Sample size for each peptide. Number of participants sampled for each peptide across different clinical phenotypes (asymptomatic, mild/moderate, severe) and time points (Day 0, 7, 14, 28).

Table S3. Associations of SARS-CoV-2 Peptides (Spike, M, NP, NSP, and ORF) with asymptomatic, mild/moderate, and severe clinical phenotypes over time. Tukey's

multiple comparisons test was used to assess differences within and between clinical phenotypes across different time points. Bold p values are significant, $p<0.05$.

Figure 1

Click here to access/download;Figure;Figure 1.tif

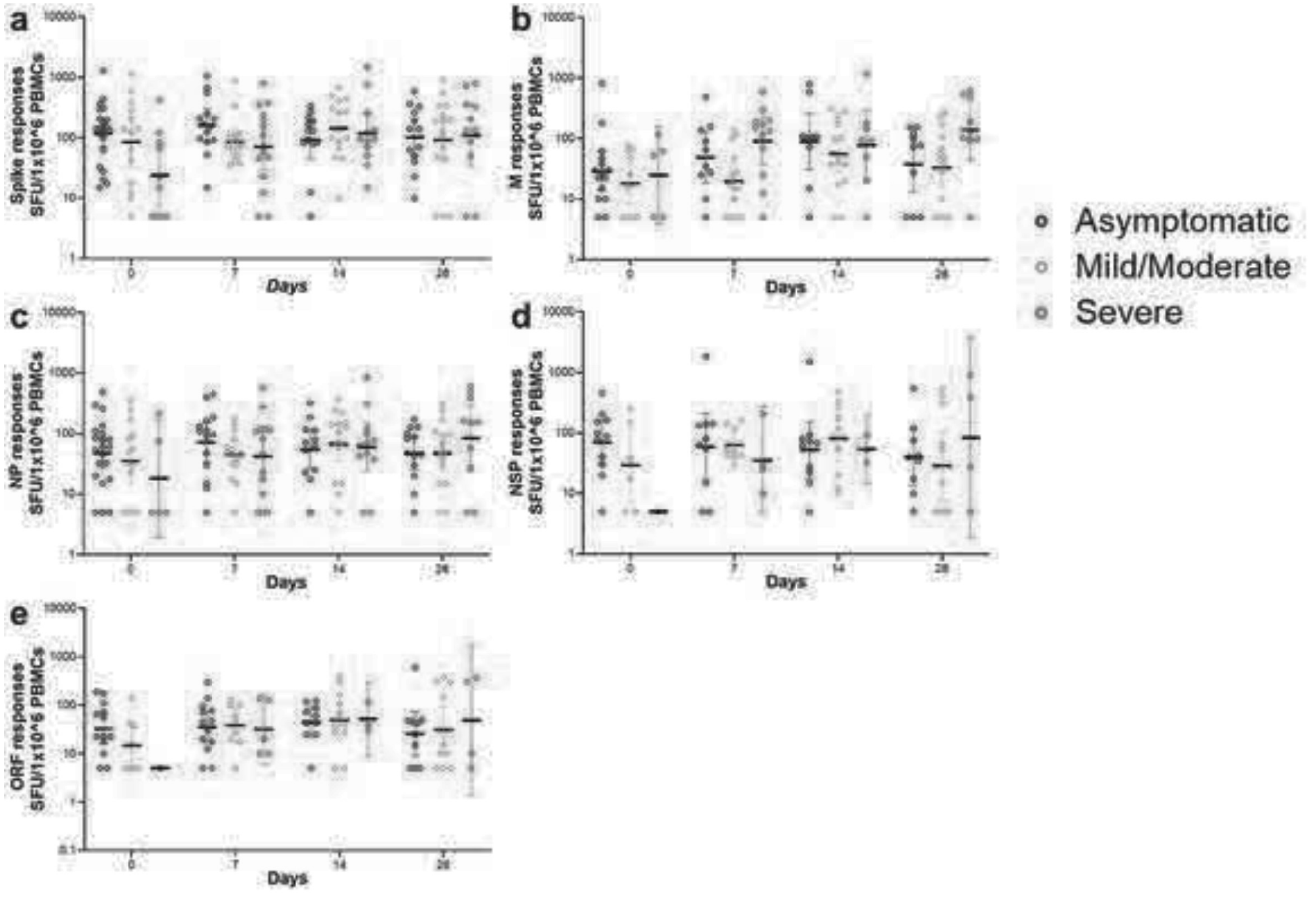

Click here to access/download;Figure;Figure 2.tif

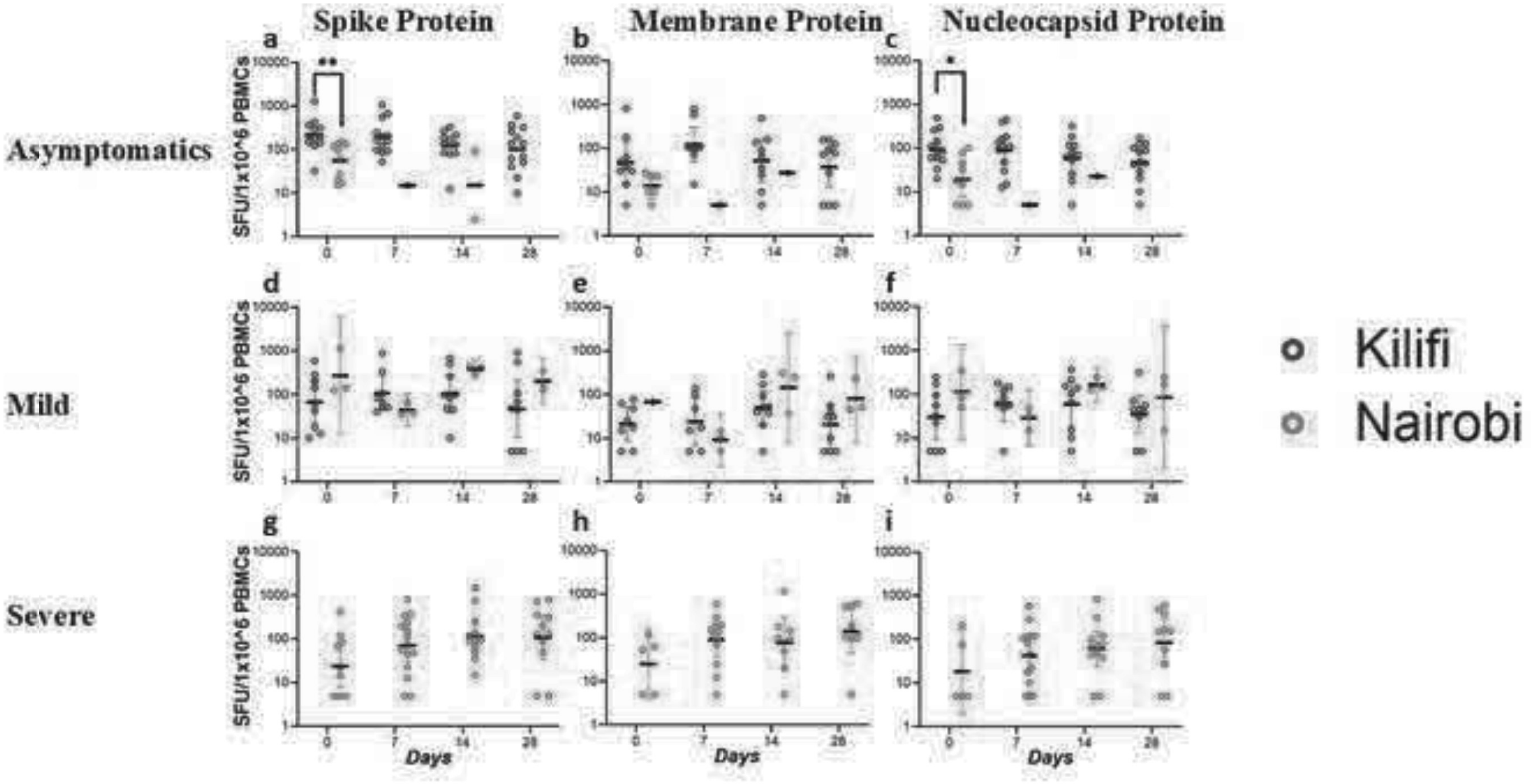

Figure 3

Click here to access/download;Figure;Figure 3.tif

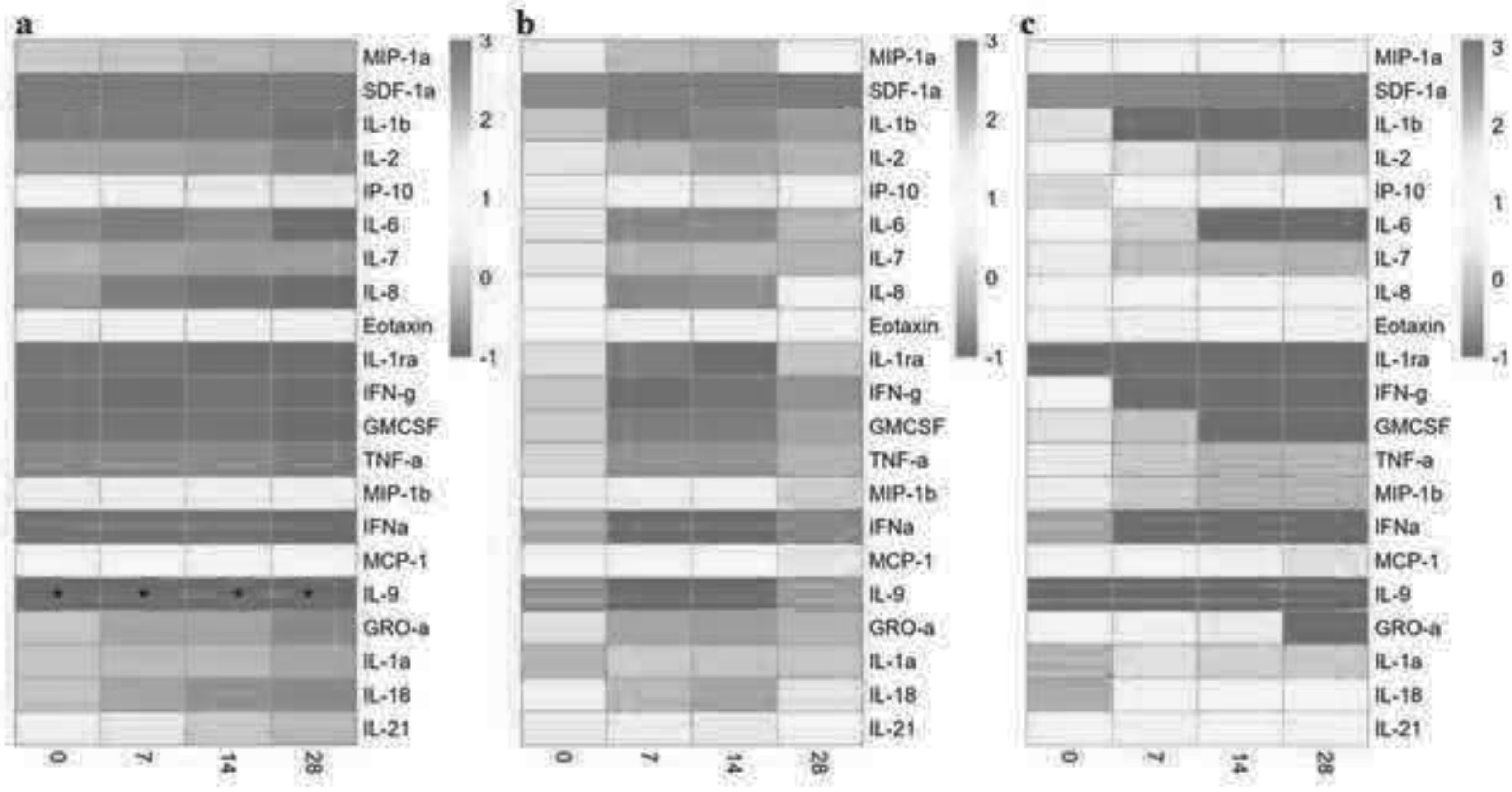

Figure 4

Click here to access/download;Figure;Figure 4.tif

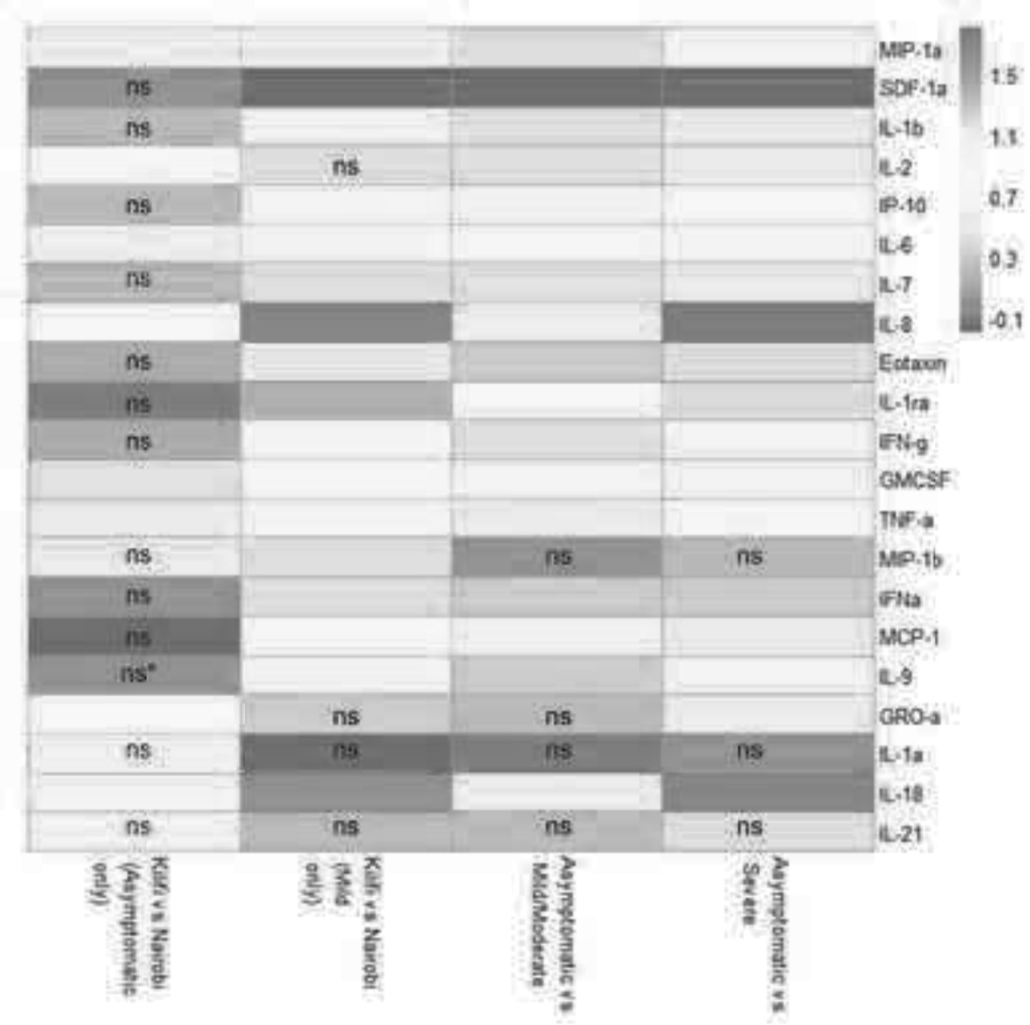

Figure 5

Click here to access/download;Figure;Figure 5.tif

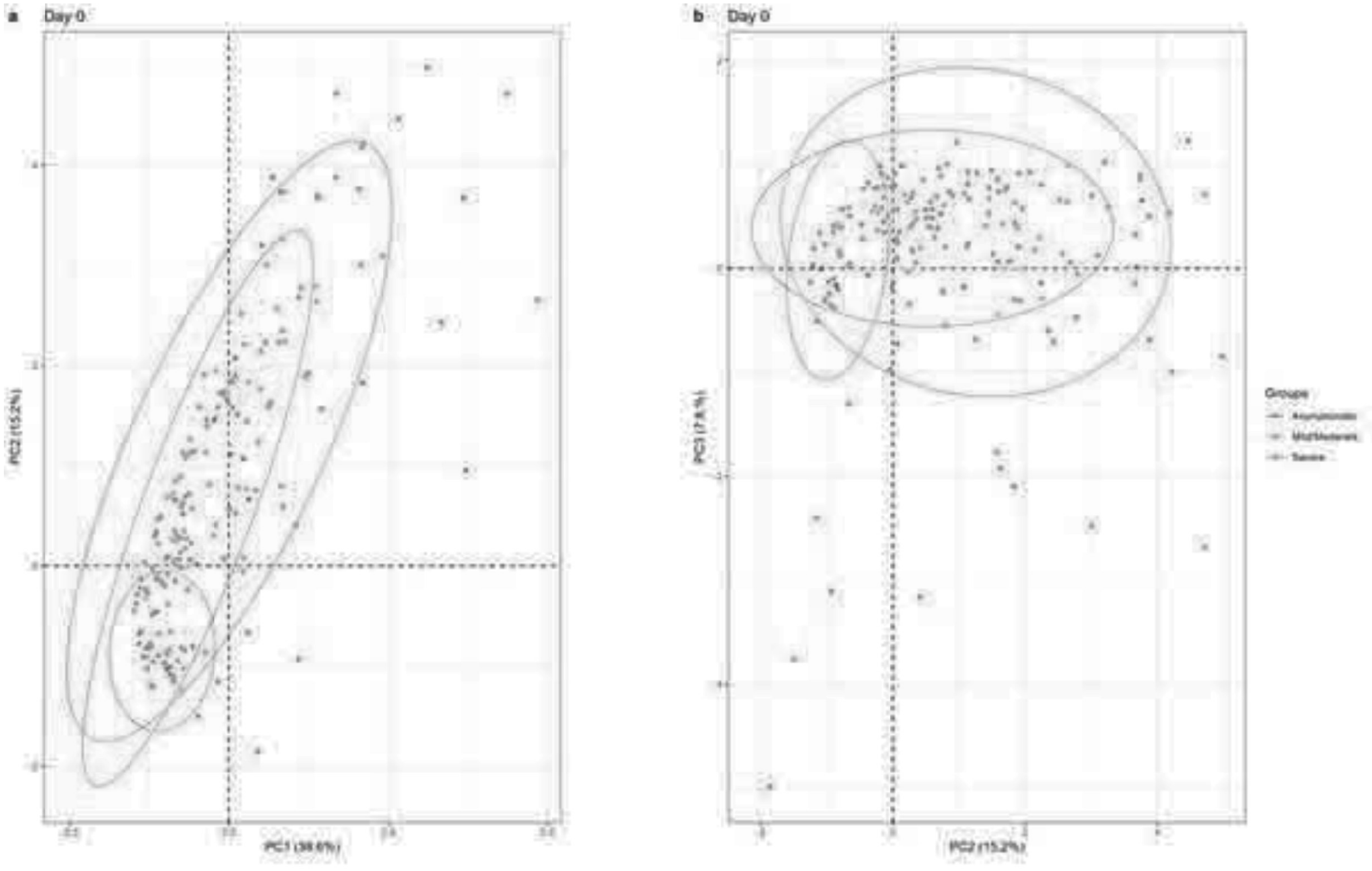

Click here to access/download
Supporting Information
Wanjiku_P6122024_Supporting information.docx

THE AGA KHAN UNIVERSITY

Faculty of Health Sciences
Medical College

Ref: 2020/IERC-55 (v5)
May 19, 2020

Dr. Reena Shah
Faculty and Principal Investigator,
Aga Khan University, Nairobi, Kenya

Dear Dr. Reena and team,

RE: COVID-19 BIOREPOSITORY AND DATA REGISTRY

The Aga Khan University, Nairobi Institutional Ethics Review Committee (IERC), is in receipt of your protocol resubmitted to the Research Office on May 14, 2020 (Protocol Version 5.0). With reference to the IERC letter Ref: 2020/IERC-55 (v4)) dated May 13, 2020, the committee has reviewed the resubmitted protocol and confirms that concerns earlier raised have been addressed.

The IERC endorses the creation of the proposed institutional biorepository of samples and information from patients who have tested positive for COVID-19. This approval is valid from **May 19, 2020** to **May 20, 2021,** and is subject to compliance with the following requirements;

1. The operations of the repository shall be governed at all times by all applicable national and international laws, rules and regulations. IERC guidelines and Aga Khan University Hospital policies shall also apply, including those specific to patient samples and records management.
2. Any **subsequent research study** to be undertaken based on data/samples obtained from the repository must be submitted to the IERC for independent review.
3. As applicable, prior to export of biological specimens/data, the PI will ensure that a Material Transfer Agreement (MTA)/Data Transfer Agreement (DTA) is in place, as well as seek shipment authority/permit from the relevant government ministry. Copies of these must be submitted to the Research Office for record purposes.
4. All consent forms must be filed in the project's study binder and patient's hospital records.
5. Further, you must provide an interim report 60 days before expiration of the validity of this approval and request extension if additional time is required.
6. You should notify the committee of any changes that may affect your project (amendments, deviations, violations, discontinuation) and a final report submitted to the Research Office for record purposes.
7. The hospital management shall be notified of any manuscripts emanating from this work.

If you have any questions, please contact Research Office at research.supportea@aku.edu or 020-366 2148/1136.

With best wishes,

Wangari

Dr. Wangari Waweru-Siika,
Chair - Institutional Ethics Review Committee (IERC)
Aga Khan University, (Kenya)

Copy – Co Investigators – Drs. Shahin Sayed, Mansoor Saleh and Rodney Adam

AK 963

3rd Parklands Avenue, off Limuru Road, P. O. Box 30270, GPO 00100, Nairobi, Kenya
Telephone: +254 20 366 2107/2109; Fax: +254 20 374 4035

KENYA MEDICAL RESEARCH INSTITUTE

P.O. Box 54840-00200, NAIROBI, Kenya
Tel: (254) 2722541, 2713349, 0722-205901, 0733-400003, Fax: (254) (020) 2720030
Email: director@kemri.org, info@kemri.org, Website. www.kemri.org

KEMRI/RES/7/3/1 **September 21, 2021**

TO: **ISABELLA OYIER**
PRINCIPAL INVESTIGATOR

THROUGH **THE DEPUTY DIRECTOR, CGMR-C**
KILIFI

Dear Madam,

RE: **KEMRI/SERU/CGMRC/200/4081 (*REQUEST FOR ANNUAL RENEWAL AND PROTOCOL DEVIATION*): INTEGRATED STUDIES OF THE NATURAL HISTORY OF SARS-COV-2 INFECTIONS IN KENYA.**

Thank you for the continuing review report for the period **September 10, 2020, to August 30, 2021.**

The Committee noted that a protocol deviation report has been submitted as the request for annual renewal was done later than the KEMRI SERU stipulated submission date.

This is to inform you that the expedited review team of the KEMRI Scientific and Ethics Review Unit (SERU) was of the informed opinion that the progress made during the reported period is satisfactory. The study has therefore been granted **approval**.

This approval is valid from **September 21, 2021** through to **September 20, 2022**. Please note that authorization to conduct this study will automatically expire on **September 20, 2022**. If you plan to continue with data collection or analysis beyond this date, please submit an application for continuing approval to the SERU by **August 09, 2022.**

You are required to submit any amendments to this protocol and any other information pertinent to human participation in this study to the SERU for review prior to initiation. You may continue with the study.

Yours faithfully,

PROF. CHARLES OBONYO,
THE ACTING HEAD,
KEMRI SCIENTIFIC AND ETHICS REVIEW UNIT.

THE AGA KHAN UNIVERSITY

Faculty of Health Sciences
Medical College

Ref: 2020/IERC-135 (v3)
December 10, 2021

Dr. Shahin Sayed - Principal Investigator
Chair and Director Laboratory Medicine,
Department of Pathology, Medical College, Aga Khan University, Nairobi

Dear Dr Sayed and team,

Re: Host immune responses to SARS-CoV-2: correlating kinetics with the natural history of infection

The Aga Khan University, Nairobi Institutional Ethics Review Committee (IERC), in receipt of your progress report submitted to the Research Office (RO) on November 12, 2021. With reference to the IERC letter Ref: 2020/IERC-135 (v2) dated December 8, 2020, the committee records that during the reporting period, the PI self-reports that:

1. No changes have been made to the study objectives.
2. No adverse effects/events have been encountered;
3. The study approval duration will expire on December 07, 2021
4. The team is requesting extension of the approval duration to continue with the study.

Based on the above considerations, the IERC has granted an extension approval for a further one year. This approval is valid until **December 06, 2022.** As PI, you are further expected to observe all the guidelines as per the study approval letter Ref: 2020/IERC-135 (v2) dated December 8, 2020

You are also reminded that researchers desiring to reinstate/continue research activities during COVID-19 pandemic must comply with the COVID-19 SOPs for Research. You are also reminded to file a copy of the research licence from the National Commission for Science, Technology and Innovation (NACOSTI) with a RO. Also submit the completed Self-Assessment Tool -Monitoring Ethical Compliance in Research.

If you have any questions, please contact AKU Research Office at AKUKenya.ResearchOffice@aku.edu or 020 366 2148/1136.

With best wishes,

Dr. Christopher Opio,
Chair - Institutional Ethics Review Committee (IERC)
Aga Khan University, (Kenya)

Copy: Co-Investigators

AK 963

3rd Parklands Avenue, off Limuru Road, P. O. Box 30270, GPO 00100, Nairobi, Kenya
Telephone: +254 20 366 2107/2109; Fax: +254 20 374 4035

Sample_ID	Time_point	Location	DateofCollectioNofSwabs
BR012	Day 0	AKUH	5/31/2020
BR012	Day 28	AKUH	5/31/2020
BR014	Day 0	AKUH	06/12/2020
BR015	Day 0	AKUH	06/10/2020
BR016	Day 0	AKUH	06/12/2020
BR016	Day 28	AKUH	06/12/2020
BR018	Day 0	AKUH	06/04/2020
BR018	Day 28	AKUH	06/04/2020
BR019	Day 0	AKUH	6/16/2020
BR020	Day 0	AKUH	6/14/2020
BR022	Day 0	AKUH	6/14/2020
BR023	Day 0	AKUH	6/21/2020
BR023	Day 28	AKUH	6/21/2020
BR025	Day 0	AKUH	6/19/2020
BR025	Day 28	AKUH	6/19/2020
BR026	Day 0	AKUH	6/17/2020
BR031	Day 0	AKUH	07/02/2020
BR033	Day 0	AKUH	07/01/2020
BR034	Day 0	AKUH	07/02/2020
BR035	Day 0	AKUH	07/04/2020
BR037	Day 0	AKUH	07/05/2020
BR037	Day 28	AKUH	07/05/2020
BR039	Day 0	AKUH	07/08/2020
BR039	Day 28	AKUH	07/08/2020
BR040	Day 0	AKUH	07/09/2020
BR040	Day 28	AKUH	07/09/2020
BR041	Day 0	AKUH	07/10/2020
BR042	Day 0	AKUH	07/11/2020
BR042	Day 28	AKUH	07/11/2020
BR043	Day 0	AKUH	07/11/2020
BR043	Day 28	AKUH	07/11/2020
BR045	Day 0	AKUH	07/11/2020
BR045	Day 28	AKUH	07/11/2020
BR046	Day 0	AKUH	07/12/2020
BR046	Day 28	AKUH	07/12/2020
BR048	Day 0	AKUH	07/11/2020
BR049	Day 0	AKUH	07/10/2020
BR049	Day 28	AKUH	07/10/2020
BR050	Day 0	AKUH	07/12/2020
BR050	Day 28	AKUH	07/12/2020
BR051	Day 0	AKUH	07/12/2020
BR051	Day 28	AKUH	07/12/2020
BR052	Day 0	AKUH	07/07/2020
BR052	Day 28	AKUH	07/07/2020
BR053	Day 0	AKUH	7/14/2020
BR053	Day 28	AKUH	7/14/2020
BR054	Day 0	AKUH	7/16/2020

BR054	Day 28	AKUH	7/16/2020
BR055	Day 0	AKUH	7/15/2020
BR055	Day 28	AKUH	7/15/2020
BR056	Day 0	AKUH	7/15/2020
BR057	Day 0	AKUH	7/17/2020
BR057	Day 28	AKUH	7/17/2020
BR058	Day 0	AKUH	7/17/2020
BR059	Day 0	AKUH	7/17/2020
BR059	Day 28	AKUH	7/17/2020
BR060	Day 0	AKUH	7/16/2020
BR060	Day 28	AKUH	7/16/2020
BR061	Day 0	AKUH	7/19/2020
BR062	Day 0	AKUH	7/17/2020
BR062	Day 28	AKUH	7/17/2020
BR064	Day 0	AKUH	7/21/2020
BR064	Day 28	AKUH	7/21/2020
BR065	Day 0	AKUH	7/19/2020
BR066	Day 0	AKUH	7/20/2020
BR068	Day 0	AKUH	7/17/2020
BR068	Day 28	AKUH	7/17/2020
BR069	Day 0	AKUH	7/17/2020
BR070	Day 0	AKUH	7/26/2020
BR072	Day 0	AKUH	7/22/2020
BR072	Day 28	AKUH	7/22/2020
BR075	Day 0	AKUH	7/14/2020
BR075	Day 28	AKUH	7/14/2020
BR080	Day 0	AKUH	7/26/2020
BR080	Day 28	AKUH	7/26/2020
BR081	Day 0	AKUH	7/27/2020
BR081	Day 28	AKUH	7/27/2020
BR082	Day 0	AKUH	7/31/2020
BR082	Day 28	AKUH	7/31/2020
BR084	Day 0	AKUH	7/31/2020
BR084	Day 28	AKUH	7/31/2020
BR087	Day 0	AKUH	08/03/2020
BR087	Day 28	AKUH	08/03/2020
BR096	Day 0	AKUH	08/08/2020
BR096	Day 28	AKUH	08/08/2020
BR097	Day 0	AKUH	7/14/2020
BR097	Day 28	AKUH	7/14/2020
BR100	Day 0	AKUH	08/07/2020
BR100	Day 28	AKUH	08/07/2020
BR103	Day 0	AKUH	8/16/2020
BR103	Day 28	AKUH	8/16/2020
BR106	Day 0	AKUH	8/23/2020
BR106	Day 28	AKUH	8/23/2020
BR107	Day 0	AKUH	8/19/2020
BR114	Day 0	AKUH	09/08/2020

BR114	Day 28	AKUH	09/08/2020
BR122	Day 0	AKUH	9/20/2020
BR122	Day 28	AKUH	9/20/2020
BR123	Day 0	AKUH	9/19/2020
BR123	Day 28	AKUH	9/19/2020
BR125	Day 0	AKUH	9/22/2020
BR125	Day 28	AKUH	9/22/2020
BR137	Day 0	AKUH	10/03/2020
BR137	Day 28	AKUH	10/03/2020
BR139	Day 0	AKUH	10/05/2020
BR139	Day 28	AKUH	10/05/2020
BR141	Day 0	AKUH	10/07/2020
BR141	Day 28	AKUH	10/07/2020
BR142	Day 28	AKUH	10/07/2020
BR142	Day 0	AKUH	10/07/2020
BR144	Day 0	AKUH	10/12/2020
BR144	Day 28	AKUH	10/12/2020
BR146	Day 0	AKUH	10/14/2020
BR146	Day 28	AKUH	10/14/2020
BR152	Day 0	AKUH	10/21/2020
BR152	Day 28	AKUH	10/21/2020
BR156	Day 0	AKUH	10/24/2020
BR157	Day 0	AKUH	10/18/2020
BR159	Day 0	AKUH	10/28/2020
BR160	Day 0	AKUH	10/25/2020
BR162	Day 0	AKUH	10/21/2020
BR162	Day 28	AKUH	10/21/2020
BR163	Day 0	AKUH	10/17/2020
BR163	Day 28	AKUH	10/17/2020
BR164	Day 0	AKUH	10/14/2020
BR164	Day 28	AKUH	10/14/2020
BR166	Day 0	AKUH	10/29/2020
BR168	Day 0	AKUH	10/28/2020
BR169	Day 0	AKUH	10/26/2020
BR169	Day 28	AKUH	10/26/2020
BR170	Day 0	AKUH	10/23/2020
BR170	Day 28	AKUH	10/23/2020
BR171	Day 0	AKUH	10/28/2020
BR171	Day 28	AKUH	10/28/2020
BR174	Day 0	AKUH	10/24/2020
BR178	Day 0	AKUH	10/25/2020
BR179	Day 0	AKUH	10/20/2020
BR180	Day 0	AKUH	10/29/2020
BR180	Day 28	AKUH	10/29/2020
BR181	Day 0	AKUH	10/30/2020
BR182	Day 0	AKUH	10/24/2020
BR182	Day 28	AKUH	10/24/2020
BR183	Day 0	AKUH	10/29/2020

BR183	Day 28	AKUH	10/29/2020
BR187	Day 0	AKUH	11/07/2020
BR187	Day 28	AKUH	11/07/2020
BR190	Day 0	AKUH	11/08/2020
BR191	Day 0	AKUH	11/03/2020
BR193	Day 0	AKUH	11/01/2020
BR193	Day 28	AKUH	11/01/2020
BR194	Day 0	AKUH	11/06/2020
BR195	Day 0	AKUH	11/08/2020
BR196	Day 0	AKUH	11/04/2020
BR196	Day 28	AKUH	11/04/2020
BR197	Day 0	AKUH	11/06/2020
BR199	Day 0	AKUH	11/04/2020
BR200	Day 0	AKUH	11/10/2020
BR201	Day 0	AKUH	11/09/2020
BR204	Day 0	AKUH	10/11/2020
BR206	Day 0	AKUH	11/06/2020
BR206	Day 28	AKUH	11/06/2020
BR207	Day 0	AKUH	11/13/2020
BR208	Day 0	AKUH	11/04/2020
BR208	Day 28	AKUH	11/04/2020
BR211	Day 0	AKUH	11/04/2020
BR212	Day 0	AKUH	11/02/2020
BR213	Day 0	AKUH	11/09/2020
BR216	Day 0	AKUH	11/14/2020
BR217	Day 0	AKUH	11/12/2020
BR220	Day 0	AKUH	11/10/2020
BR221	Day 0	AKUH	11/22/2020
BR224	Day 0	AKUH	11/21/2020
BR225	Day 0	AKUH	11/10/2020
BR226	Day 0	AKUH	11/18/2020
BR226	Day 28	AKUH	11/18/2020
BR227	Day 0	AKUH	11/16/2020
BR228	Day 0	AKUH	11/21/2020
BR228	Day 28	AKUH	11/21/2020
BR229	Day 0	AKUH	11/17/2020
BR229	Day 28	AKUH	11/17/2020
BR231	Day 0	AKUH	11/26/2020
BR234	Day 0	AKUH	11/21/2020
BR236	Day 0	AKUH	11/23/2020
BR236	Day 28	AKUH	11/23/2020
BR237	Day 0	AKUH	11/24/2020
BR238	Day 0	AKUH	11/23/2020
BR238	Day 28	AKUH	11/23/2020
BR240	Day 0	AKUH	11/15/2020
BR242	Day 0	AKUH	11/30/2020
BR243	Day 0	AKUH	11/30/2020
BR244	Day 0	AKUH	11/26/2020

BR245	Day 0	AKUH	12/01/2020
BR246	Day 0	AKUH	11/30/2020
BR246	Day 28	AKUH	11/30/2020
BR248	Day 0	AKUH	12/02/2020
IMCA-002	Day 0	AKUH	16/01/2021
IMCA-002	Day 14	AKUH	28/01/2021
IMCA-002	Day 28	AKUH	10/02/2021
IMCA-002	Day 7	AKUH	22/01/2021
IMCA-002A	Day 0	AKUH	27/08/2021
IMCA-002A	Day 14	AKUH	10/09/2021
IMCA-004A	Day 0	AKUH	28/08/2021
IMCA-004A	Day 14	AKUH	12/09/2021
IMCA-005	Day 0	AKUH	11/02/2021
IMCA-005	Day 7	AKUH	18/02/2021
IMCA-006	Day 0	AKUH	11/02/2021
IMCA-007	Day 14	AKUH	11-02-2021
IMCA-007A	Day 0	AKUH	02/09/2021
IMCA-007A	Day 7	AKUH	12/09/2021
IMCA-008	Day 0	AKUH	18/02/2021
IMCA-008	Day 14	AKUH	04/03/2021
IMCA-008	Day 7	AKUH	25/02/2021
IMCA-009	Day 0	AKUH	18/02/2021
IMCA-009	Day 14	AKUH	04/03/2021
IMCA-009	Day 28	AKUH	17/03/2021
IMCA-009	Day 7	AKUH	25/02/2021
IMCA-010	Day 0	AKUH	18/02/2021
IMCA-010	Day 14	AKUH	04/03/2021
IMCA-010	Day 28	AKUH	22/03/2021
IMCA-010	Day 7	AKUH	25/02/2021
IMCA-010A	Day 0	AKUH	04/09/2021
IMCA-010A	Day 7	AKUH	11/09/2021
IMCA-012	Day 0	AKUH	03/03/2021
IMCA-012	Day 14	AKUH	19/03/2021
IMCA-012	Day 28	AKUH	01/04/2021
IMCA-013	Day 0	AKUH	03/03/2021
IMCA-013	Day 14	AKUH	18/03/2021
IMCA-013	Day 28	AKUH	01/04/2021
IMCA-032	Day 0	AKUH	25/03/2021
IMCA-032	Day 28	AKUH	22/04/2021
IMCA-032	Day 7	AKUH	01/04/2021
IMCA-033	Day 0	AKUH	25/03/2021
IMCA-033	Day 14	AKUH	13/04/2021
IMCA-033	Day 28	AKUH	22/04/2021
IMCA-033	Day 7	AKUH	01/04/2021
IMCA-038	Day 0	AKUH	26/03/2021
IMCA-038	Day 14	AKUH	13/04/2021
IMCA-038	Day 28	AKUH	22/04/2021
IMCA-046	Day 0	AKUH	14/04/2021

IMCA-046	Day 14	AKUH	05/05/2021
IMCA-046	Day 28	AKUH	18/05/2021
IMCA-046	Day 7	AKUH	23/04/2021
IMCA-050	Day 0	AKUH	01/07/2021
IMCA-050	Day 14	AKUH	16/07/2021
IMCA-050	Day 28	AKUH	27/07/2021
IMCA-050	Day 7	AKUH	07/07/2021
IMCA-052	Day 0	AKUH	08/07/2021
IMCA-052	Day 14	AKUH	03/08/2021
IMCA-052	Day 7	AKUH	16/07/2021
IMCA-053	Day 0	AKUH	08/07/2021
IMCA-053	Day 14	AKUH	03/08/2021
IMCA-053	Day 7	AKUH	16/07/2021
IMCA-055	Day 0	AKUH	03/08/2021
IMCA-055	Day 14	AKUH	19/08/2021
IMCA-055	Day 28	AKUH	07/09/2021
IMCA-055	Day 7	AKUH	10/08/2021
IMCA-056	Day 0	AKUH	03/08/2021
IMCA-056	Day 14	AKUH	16/08/2021
IMCA-056	Day 28	AKUH	31/08/2021
IMCA-056	Day 7	AKUH	11-08-2021
IMCA-057	Day 0	AKUH	16/08/2021
IMCA-057	Day 14	AKUH	26/08/2021
IMCA-057	Day 28	AKUH	14/09/2021
IMCA-057	Day 7	AKUH	23/08/2021
IMCA-058	Day 0	AKUH	19/08/2021
IMCA-058	Day 7	AKUH	23/08/2021
IMCA-059	Day 0	AKUH	23/08/2021
IMCA-059	Day 14	AKUH	31/08/2021
IMCA-059	Day 28	AKUH	16/09/2021
IMCA-059	Day 7	AKUH	26/08/2021
IMC-K002	Day 0	Kilifi	3/24/2021
IMC-K002	Day 14	Kilifi	3/24/2021
IMC-K002	Day 28	Kilifi	3/24/2021
IMC-K002	Day 7	Kilifi	3/24/2021
IMC-K004	Day 0	Kilifi	04/01/2021
IMC-K004	Day 14	Kilifi	04/01/2021
IMC-K004	Day 28	Kilifi	04/01/2021
IMC-K004	Day 7	Kilifi	04/01/2021
IMC-K005	Day 0	Kilifi	04/12/2021
IMC-K005	Day 7	Kilifi	04/12/2021
IMC-K006	Day 0	Kilifi	4/20/2021
IMC-K006	Day 14	Kilifi	4/20/2021
IMC-K006	Day 28	Kilifi	4/20/2021
IMC-K006	Day 7	Kilifi	4/20/2021
IMC-K007	Day 0	Kilifi	4/21/2021
IMC-K007	Day 14	Kilifi	4/21/2021
IMC-K007	Day 28	Kilifi	4/21/2021

IMC-K007	Day 7	Kilifi	4/21/2021
IMC-K008	Day 0	Kilifi	4/26/2021
IMC-K008	Day 14	Kilifi	4/26/2021
IMC-K008	Day 28	Kilifi	4/26/2021
IMC-K008	Day 7	Kilifi	4/26/2021
IMC-K009	Day 0	Kilifi	4/28/2021
IMC-K009	Day 28	Kilifi	4/28/2021
IMC-K009	Day 7	Kilifi	4/28/2021
IMC-K010	Day 0	Kilifi	05/07/2021
IMC-K010	Day 14	Kilifi	05/07/2021
IMC-K010	Day 28	Kilifi	05/07/2021
IMC-K010	Day 7	Kilifi	05/07/2021
IMC-K011	Day 0	Kilifi	05/07/2021
IMC-K011	Day 14	Kilifi	05/07/2021
IMC-K011	Day 7	Kilifi	05/07/2021
IMC-K012	Day 0	Kilifi	5/13/2021
IMC-K012	Day 14	Kilifi	5/13/2021
IMC-K012	Day 28	Kilifi	5/13/2021
IMC-K012	Day 7	Kilifi	5/13/2021
IMC-K014	Day 0	Kilifi	5/21/2021
IMC-K014	Day 14	Kilifi	5/21/2021
IMC-K014	Day 28	Kilifi	5/21/2021
IMC-K014	Day 7	Kilifi	5/21/2021
IMC-K015	Day 0	Kilifi	5/21/2021
IMC-K015	Day 14	Kilifi	5/21/2021
IMC-K015	Day 28	Kilifi	5/21/2021
IMC-K015	Day 7	Kilifi	5/21/2021
IMC-K016	Day 0	Kilifi	06/04/2021
IMC-K016	Day 14	Kilifi	06/04/2021
IMC-K016	Day 28	Kilifi	06/04/2021
IMC-K016	Day 7	Kilifi	06/04/2021
IMC-K017	Day 0	Kilifi	06/04/2021
IMC-K017	Day 14	Kilifi	06/04/2021
IMC-K017	Day 7	Kilifi	06/04/2021
IMC-K019	Day 0	Kilifi	6/17/2021
IMC-K019	Day 7	Kilifi	6/17/2021
IMC-K023	Day 0	Kilifi	6/28/2021
IMC-K023	Day 14	Kilifi	6/28/2021
IMC-K023	Day 28	Kilifi	6/28/2021
IMC-K023	Day 7	Kilifi	6/28/2021
IMC-K024	Day 0	Kilifi	6/30/2021
IMC-K024	Day 14	Kilifi	6/30/2021
IMC-K024	Day 28	Kilifi	6/30/2021
IMC-K024	Day 7	Kilifi	6/30/2021
IMC-K025	Day 0	Kilifi	07/02/2021
IMC-K025	Day 14	Kilifi	07/02/2021
IMC-K025	Day 28	Kilifi	07/02/2021
IMC-K025	Day 7	Kilifi	07/02/2021

IMC-K026	Day 0	Kilifi	07/07/2021
IMC-K026	Day 14	Kilifi	07/07/2021
IMC-K026	Day 7	Kilifi	07/07/2021
IMC-K027	Day 0	Kilifi	7/16/2021
IMC-K027	Day 14	Kilifi	7/16/2021
IMC-K027	Day 28	Kilifi	7/16/2021
IMC-K027	Day 7	Kilifi	7/16/2021
IMC-K029	Day 0	Kilifi	7/23/2021
IMC-K029	Day 14	Kilifi	7/23/2021
IMC-K029	Day 28	Kilifi	7/23/2021
IMC-K029	Day 7	Kilifi	7/23/2021
IMC-K030	Day 0	Kilifi	7/28/2021
IMC-K030	Day 14	Kilifi	7/28/2021
IMC-K031	Day 0	Kilifi	10/14/2021
IMC-K031	Day 14	Kilifi	10/14/2021
IMC-K031	Day 28	Kilifi	10/14/2021
IMC-K031	Day 7	Kilifi	10/14/2021
IMC-K032	Day 0	Kilifi	10/21/2021
IMC-K032	Day 7	Kilifi	10/21/2021
IMC-K033	Day 0	Kilifi	11/04/2021
IMC-K033	Day 14	Kilifi	11/04/2021
IMC-K033	Day 28	Kilifi	11/04/2021
IMC-K033	Day 7	Kilifi	11/04/2021
IMC-K034	Day 0	Kilifi	12/20/2021
IMC-K034	Day 7	Kilifi	12/20/2021
IMC-K035	Day 0	Kilifi	01/10/2022
IMC-K035	Day 14	Kilifi	01/10/2022
IMC-K035	Day 28	Kilifi	01/10/2022
IMC-K035	Day 7	Kilifi	01/10/2022
IMC-K037	Day 0	Kilifi	1/13/2022
IMC-K037	Day 14	Kilifi	1/13/2022
IMC-K037	Day 28	Kilifi	1/13/2022
IMC-K037	Day 7	Kilifi	1/13/2022
IMC-K038	Day 0	Kilifi	1/13/2022
IMC-K038	Day 14	Kilifi	1/13/2022
IMC-K038	Day 28	Kilifi	1/13/2022
IMC-K038	Day 7	Kilifi	1/13/2022
IMC-K039	Day 0	Kilifi	1/19/2022
IMC-K039	Day 14	Kilifi	1/19/2022
IMC-K039	Day 28	Kilifi	1/19/2022
IMC-K039	Day 7	Kilifi	1/19/2022
IMC-K040	Day 0	Kilifi	23-06-2022
IMC-K040	Day 14	Kilifi	07-07-2022
IMC-K040	Day 28	Kilifi	21-07-2022
IMC-K040	Day 7	Kilifi	29-06-2022
IMC-K043	Day 0	Kilifi	04-07-2022
IMC-K043	Day 14	Kilifi	18-07-2022
IMC-K043	Day 28	Kilifi	01-08-2022

IMC-K043	Day 7	Kilifi	12-07-2022
IMC-K044	Day 0	Kilifi	04-07-2022
IMC-K044	Day 14	Kilifi	18-07-2022
IMC-K044	Day 28	Kilifi	01-08-2022
IMC-K044	Day 7	Kilifi	12-07-2022
IMC-K045	Day 0	Kilifi	06-07-2022
IMC-K045	Day 14	Kilifi	21-07-2022
IMC-K045	Day 28	Kilifi	02-08-2022
IMC-K045	Day 7	Kilifi	13-07-2022
IMC-K046	Day 0	Kilifi	08-07-2022
IMC-K046	Day 14	Kilifi	22-07-2022
IMC-K046	Day 28	Kilifi	04-08-2022
IMC-K046	Day 7	Kilifi	15-07-2022
IMC-K047	Day 0	Kilifi	18-07-2022
IMC-K047	Day 14	Kilifi	01-08-2022
IMC-K047	Day 28	Kilifi	12-08-2022
IMC-K047	Day 7	Kilifi	26-07-2022

1. AKU_IERC Approval Letter Biorepository protocol.
    a. Covers samples with sample IDs starting with **BR**.
    b. Samples included in this study were collected within the approved ethics period: **May 19, 2020, to May 19, 2021**.

The remaining samples, with sample IDs starting with **IMCA-** or **IMC-K**, are covered by the following ethics approvals:

EMRI_SERU4081_ImmunoCOV_ApprovalLetter_Del1_1

a. Ethics approval period: **September 10, 2020, to September 9, 2021**.

3. KEMRI_SERU4081_ImmunoCOV_ethics approval renewal 2021-2022
    a. Ethics approval period: **September 21, 2021, to September 20, 2022**.

KU_IERC_ImmunoCoV_Approval_Letter_Del1_1

a. Approval period: **December 8, 2020, to December 7, 2021**.

5. AKU_ImmunoCoV_IERC Approval_Renewal_2021_2022
    a. Approval period: **December 8, 2021, to December 6, 2022**.
