# Supplementary material for: Induction of an early IFN-γ cellular response and high plasma levels of SDF-1α are inversely associated with COVID-19 severity and residence in rural areas in Kenyan patients
Source: PLoS One. 2025 Sep 11;20(9):e0316967. doi: 10.1371/journal.pone.0316967 (PMC12425234; doi:10.1371/journal.pone.0316967)
Supplement: Attachment — Submitted filename: Ethics Approval Documentation and Sample Collection Details.docx [file pone.0316967.s012.docx]

I have uploaded the ethics approval documents along with the necessary renewals.

Additionally, I have uploaded the sample IDs with their corresponding dates of collection.

Please see the explanation below, demonstrating that ethical approval was obtained prior to sample collection.

1. AKU_IERC Approval Letter Biorepository protocol.
   a. Covers samples with sample IDs starting with **BR**.
   b. Samples included in this study were collected within the approved ethics period: **May 19, 2020, to May 19, 2021**.

The remaining samples, with sample IDs starting with **IMCA-** or **IMC-K**, are covered by the following ethics approvals:

2. KEMRI_SERU4081_ImmunoCOV_ApprovalLetter_Del1_1
   a. Ethics approval period: **September 10, 2020, to September 9, 2021**.
3. KEMRI_SERU4081_ImmunoCOV_ethics approval renewal 2021-2022
a. Ethics approval period: **September 21, 2021, to September 20, 2022**.
4. AKU_IERC_ImmunoCoV_Approval_Letter_Del1_1
   a. Approval period: **December 8, 2020, to December 7, 2021**.
5. AKU_ImmunoCoV_IERC Approval_Renewal_2021_2022
a. Approval period: **December 8, 2021, to December 6, 2022**.
