# Supplementary material for: Induction of an early IFN-γ cellular response and high plasma levels of SDF-1α are inversely associated with COVID-19 severity and residence in rural areas in Kenyan patients
Source: PLoS One. 2025 Sep 11;20(9):e0316967. doi: 10.1371/journal.pone.0316967 (PMC12425234; doi:10.1371/journal.pone.0316967)
Supplement: Attachment — Submitted filename: PWanjiku_P07052025_ Response to Reviewers.docx [file pone.0316967.s014.docx]

25^th^ April 2025

The Editors

PLOS ONE-
1875 Mission Street
Suite 103 #188 San Francisco, CA 94103
United States.

Dear Editors,

Revisions to manuscript entitled “**Induction of an early IFN-γ cellular response and high plasma levels of SDF-1α are inversely associated with COVID-19 severity and residence in rural areas in Kenyan patients.**”

We thank you and the reviewers for the thoughtful and constructive comments provided which have helped to greatly improve our manuscript. We have carefully considered all the suggestions and revised the manuscript accordingly to address the reviewers' concern.

Below, we provide detailed responses outlining how each comment was addressed.

All the reviewers’ comments and requests are in *italics*, and our responses are in normal font text. The actual changes that were made in the manuscript are in **bold** text, and we have indicated the first page and line of the manuscript they have been inserted. Within the manuscript, these changes are tracked.

*Reviewer #1:*

*I thoroughly enjoyed reading the manuscript. It is well-written, with findings clearly articulated and thoughtfully discussed. Only minor corrections are needed, along with a few adjustments to meet the publication criteria.*

1. *The abstract exceeds the 300-word limit required by the publication criteria. Please revise it to ensure compliance.*

Response: We have now revised the abstract to 299 words. (Page 2 of the manuscript)

2. *A minor grammatical error in the introduction (line 82).*

Response: Thank you for highlighting this grammatical error in the introduction. We have now corrected it accordingly.

Page 4, line 82.

3. *According to the publication criteria, the introduction must include a brief statement of the overall aim of the study and a comment on whether the aim was achieved. While the aim is stated, there is no mention of its achievement. Please revise.*

Response: Thank you for your helpful feedback. We have revised the introduction to include a clear comment on whether the aim of the study was achieved, in accordance with the publication criteria.

Page 5, line 106-108. **<** **Our findings demonstrate that both IFN-γ cellular, and plasma cytokine and chemokine response profiles, among COVID-19 Kenyan patients, varied by different disease phenotypes and geographic locations.>**

4. *The study design is robust, with well-defined patient groups and clear cytokine/chemokine measurement methods. However, can you clarify how the missing data was handled in statistical analysis.*

Response: We thank the reviewer for this helpful critique. We have now addressed the missing data problem in the statistical analysis section and have included a detailed paragraph in the supporting information document. We have also included the implications of the missing data points in the discussion.

*Page 12, line 251-258:* **< A total of 98 (20%) data values of the expected 498 were missing due to a variety of reasons including design, participants moving out of the study area after hospital discharge, insufficient PBMC numbers or plasma volumes, and unavailability of some participants during scheduled study visits. However, after exploration of the missing data (see supporting information, Figure S1 and Table S3) coupled with the likelihood approaches, the analyses are valid under the missing at random mechanism (36).>**

Page 25, line 509-511: Discussion. **<These gaps resulted in missing data for certain timepoints or peptide stimulations, which may have reduced the statistical power of some comparisons.>**

5. *The experiment was conducted rigorously, with appropriate controls and sample sizes. The conclusion appropriately summarizes the results, emphasizing the association between IFN-gamma response, cytokine levels and COVID-19 severity. However, I suggest you include a discussion on potential confounding variables such as comorbidities. This could strengthen the argument.*

Response: Thank you for this insightful comment. We agree that comorbidities are important potential confounders that may influence cytokine levels and IFN-γ responses in COVID-19 patients. We have added a statement in the discussion to reflect the possible impact of comorbidities on our findings.

Page 23, line 473-481: **<As was observed in previous studies, individuals with severe COVID-19 had experienced more frequencies of comorbidities such as diabetes, hypertension, cardiovascular diseases, and respiratory diseases than those with asymptomatic infection and moderate symptoms. These comorbidities may also influence immune responses by modulating angiotensin-converting enzyme 2 (ACE2) expression, altering cytokine and chemokine profiles (63) , and hence are potential confounders in the correlational analysis.>**

6. *The discussion is well-structured, but I suggest integrating more comparisons with similar studies* *outside Sub-Saharan Africa.*

Response: We thank the reviewer for this insightful comment. In response, we have expanded the discussion to highlight comparisons with studies conducted outside Sub-Saharan Africa for example in China, Bulgaria, Hong Kong, Ireland, Egypt, and Iran.

Page 22, line 431-433: **<** **As had been seen in earlier reports from Singapore, Netherlands, Italy, and Brazil which associated increased IFN-γ secreting cells measured by ELISpot assay in the early phase of infection with milder disease (42–45), >**

Page 23, line 462-465: **<We demonstrate that elevated levels of eighteen cytokines and chemokines were associated with severe COVID-19, in agreement with previous reports from China, Bulgaria, Rwanda, Hong Kong, Kenya, Ireland, Egypt and Iran, which associated these markers to disease severity, adverse outcomes, severe lung injury and ARDS (18–23,29,57–59).>**

7. *Figures and tables effectively present the data but should be closely examined for consistency in labeling (example: units, statistical significance markers).*

Response: We apologize for the errors. We have now corrected the labelling of the axes and headings for figures x and y for consistency.

8. *The References are comprehensive, but a few citations should be updated to include recent studies.*

Response: We have reviewed more recent literature and updated the manuscript as suggested.

**2.	Bejon P, Agweyu A, Ochola-Oyier LI, Hamaluba M, Kamuya D, Kinyanjui S, et al. Rethinking the evidence on COVID-19 in Africa. Lancet Infect Dis [Internet]. 2025 Apr 4 [cited 2025 May 7];0(0). Available from: https://www.thelancet.com/journals/laninf/article/PIIS1473-3099(25)00071-4/abstract**

**29.	Sang JC, Musyoki SK, Injera WE, Karani LW, Maiyoh GK. Cytokine immune profiles among COVID 19 patients with different disease severities seeking treatment at Moi teaching and referral hospital, Kenya. Cytokine. 2025 Jun 1;190:156917.** **45.	Pena NM, Santana LC, Hunter JR, Blum VF, Vergara T, Gouvea C, et al. T cell-mediated Immune response and correlates of inflammation and their relationship with COVID-19 clinical severity: not an intuitive guess. BMC Infect Dis. 2024 Jun 20;24(1):612.**

**57.	Hawerkamp HC, Dyer AH, Patil ND, McElheron M, O’Dowd N, O’Doherty L, et al. Characterisation of the pro-inflammatory cytokine signature in severe COVID-19. Front Immunol [Internet]. 2023 Mar 30 [cited 2025 Apr 26];14. Available from:** [**https://www.frontiersin.orghttps://www.frontiersin.org/journals/immunology/articles/10.3389/fimmu.2023.1170012/full**](https://www.frontiersin.orghttps://www.frontiersin.org/journals/immunology/articles/10.3389/fimmu.2023.1170012/full)

**58.	Salem ML, Eltoukhy MM, Shalaby RE, Okasha KM, El-Shanshoury MR, Attia MA, et al. COVID-19 Severity Shifts the Cytokine Milieu Toward a Proinflammatory State in Egyptian Patients: A Cross-Sectional Study. J Interferon Cytokine Res. 2023 Jun;43(6):257–68.**

**59.	Ghaffarpour S, Ghazanfari T, Ardestani SK, Naghizadeh MM, Vaez Mahdavi MR, Salehi M, et al. Cytokine profiles dynamics in COVID-19 patients: a longitudinal analysis of disease severity and outcomes. Sci Rep. 2025 Apr 23;15(1):14209.**

Reviewer #2:
*The study investigates the immunological factors that might explain the lower severity of COVID-19 in Sub-Saharan Africa (SSA) compared to Europe and North America. It focuses on the levels of ex vivo SARS-CoV-2 peptide-specific IFN-γ producing cells and plasma cytokines and chemokines over the first month of COVID-19 diagnosis among Kenyan patients from urban (Nairobi) and rural (Kilifi) areas.
Methods:
• Participants: 188 COVID-19 patients from Nairobi (urban, n = 152) and Kilifi (rural, n = 36) were recruited and monitored longitudinally.
• Assays: IFN-γ secreting cells were enumerated using an ex vivo enzyme-linked immunospot (ELISpot) assay, and levels of 22 plasma cytokines and chemokines were measured using a multiplexed binding assay.
Results:
• IFN-γ Response: Higher frequencies of IFN-γ-secreting cells against SARS-CoV-2 spike peptides were observed on the day of diagnosis among asymptomatic patients compared to those with severe COVID-19.
• Cytokines and Chemokines: Higher concentrations of 17 out of 22 cytokines and chemokines were positively associated with severe disease, particularly IL-8, IL-18, and IL-1ra, while lower concentrations of SDF-1α were associated with severe disease.
• Geographical Differences: Concentrations of 8 and 16 cytokines and chemokines, including IL-18, were higher among Nairobi asymptomatic and mild patients compared to their Kilifi counterparts. Conversely, SDF-1α concentrations were higher in rural Kilifi compared to Nairobi.
Conclusion:
Pro-inflammatory cytokines and chemokines were associated with severe COVID-19, while an early IFN-γ cellular response to overlapping SARS-CoV-2 spike peptides was associated with reduced risk of disease. Living in urban Nairobi was associated with increased levels of pro-inflammatory cytokines/chemokines compared to rural Kilifi.*

Loopholes in the Results and Discussion:

1. *Sample Size and Recruitment Issues: The study faced difficulties recruiting asymptomatic patients in Nairobi and severe cases in Kilifi, leading to a relatively small sample size. This limitation could affect the generalizability of the findings.*

Response: We acknowledge and agree with the reviewer’s comment regarding the recruitment challenges, which resulted in fewer asymptomatic participants in Nairobi, which may affect the generalizability of our findings. This limitation has now been explicitly highlighted/discussed in the revised manuscript. We acknowledge the fact that we had fewer severe COVID-19 patients, but this is also a reflection of the paucity of such patients in rural areas.

We have updated the limitations section to reflect this point and to acknowledge how these recruitment patterns could influence the interpretation and broader applicability of our results.

*Page 25, line 512-517:* **<.** **Secondly, we had difficulties recruiting asymptomatic patients in Nairobi and were unable to recruit severe cases in Kilifi, resulting in an imbalanced distribution across clinical phenotypes and thus the sample size is relatively small. These limitations may affect the generalizability of our findings, particularly in comparisons of disease severity between the two geographic locations. Future studies should aim to include a more balanced and diverse sample set to improve representativeness.>**

2. *Missing Data: Some data are incomplete for certain patients due to missed follow-ups or unavailability of adequate numbers of PBMCs to quantify IFN-γ cellular responses to the full spectrum of peptide pools.*

Response: We thank the reviewer for this helpful critique. We have now addressed the missing data problem in the statistical analysis section and have included a detailed paragraph in the supporting information document. We have also included the implications of the missing data points in the discussion.

See the same response to Reviewer 1, question 4 above.

3. Geographical Differences: The study suggests that the immune response to SARS-CoV-2 is less inflammatory among residents of rural areas. However, the differences in cytokine and chemokine levels between urban and rural patients might also be influenced by other factors such as environmental exposures and lifestyle differences.

Response: We agree that the observed differences in cytokine and chemokine levels between urban and rural patients may be influenced by different environmental exposures and lifestyles. Unfortunately, our study did not collect data on these variables, and we were therefore unable to directly assess their impact. However, we have acknowledged this in the discussion.

*Page 24, line 495-503:* **< The factors underlying this observation may include differences in previous environmental exposures such as air pollution and microbes, as well as differences in lifestyles including nutritional** **and behavioral factors (66–68). Thus, despite differences in levels of SARS-CoV-2 exposure, the immune systems of urban and rural residents may have had been pre-conditioned to respond differently to SARS-CoV-2, with those from urban areas being more likely to produce higher levels of inflammatory cytokines, which were then associated with severe COVID-19.>**

4. The study does not provide a background on cellular response data against COVID-19 data available in the Kenyan population.

Response: We thank the reviewer for highlighting this critical gap in our study. We have now updated the introduction to include available although very few studies on T cell and other cellular immune responses to SARS-CoV-2 in Kenya.

*Page 5, line 91-100:* **< An association between severe COVID-19 and elevated levels of pro-inflammatory cytokines such as IFN-γ, TNF-α, and IL-17, as well as anti-inflammatory cytokines including IL-6 and IL-10, have been reported (29). Furthermore, Samandari et al. (30) demonstrated the presence of SARS-CoV-2-specific T cell responses among asymptomatic Kenyans, pointing to a possible role of cellular immunity in protection against severe disease. Together, these studies highlight the importance of characterizing cellular immune responses in the local context to better understand disease outcomes.>**

We are grateful for the opportunity to address these concerns and trust that our revisions and responses will be met with favourable review. Please let me know if I can be of any further assistance.

Thank you for your continued consideration of the manuscript.

Yours sincerely,

Perpetual Wanjiku

KEMRI-Wellcome Trust Research Programme
