# Supplementary material for: Induction of an early IFN-γ cellular response and high plasma levels of SDF-1α are inversely associated with COVID-19 severity and residence in rural areas in Kenyan patients
Source: PLoS One. 2025 Sep 11;20(9):e0316967. doi: 10.1371/journal.pone.0316967 (PMC12425234; doi:10.1371/journal.pone.0316967)
Supplement: Attachment — Submitted filename: Reviewer 2 comments.docx [file pone.0316967.s015.docx]

Reviewer 2:

I thank the author’s for taking time to revise the manuscript. However, I do have some comments for the authors below.

**Strengths**

1. **Relevance and Contextualization**
   The introduction effectively frames the paradox of lower COVID-19 severity in Sub-Saharan Africa (SSA) despite widespread transmission and weaker health systems. This sets up a compelling rationale for the study.
2. **Comprehensive Literature Integration**
   The manuscript references a wide range of studies (1–30), covering epidemiological, immunological, and environmental hypotheses. This breadth demonstrates a strong grasp of the current discourse.
3. **Clear Research Gap and Objective**
   The transition from global observations to the Kenyan context is well-articulated. The lack of data on cellular immunity in Kenyan patients is clearly identified as a gap, and the study’s aim is explicitly stated.

**Comments**

**1. Structure and Flow**

- **Redundancy**: The final sentence of the introduction (lines 107–111) is repeated almost verbatim. This should be consolidated into a single, clear statement of the study’s objective.
- **Paragraph Transitions**: The flow between paragraphs could be improved. For example, the shift from global hypotheses (lines 65–71) to cytokine storm mechanisms (lines 72–83) is abrupt. Consider adding a bridging sentence.

**2. Clarity and Precision**

- **Terminology**: The phrase “plethora of inflammatory host responses” (line 72) is vague. Consider specifying “a broad range of pro-inflammatory cytokines and chemokines.”
- **Ambiguity in Causality**: The sentence “Thus, there is no evidence of the primary acute antibody response controlling the infection outcome” (lines 85–86) is too strong. Consider softening to “suggesting that antibody responses alone may not determine infection outcome.”
- Line 94: The sentence structure is awkward: “…as well as anti-inflammatory cytokines including IL-6 and IL-10, have been reported.” Consider rephrasing for clarity.

**3. Scientific Rigor**

- **Balance in Hypotheses**: While the introduction lists many plausible explanations for the SSA paradox, it does not critically evaluate them. A brief comment on the limitations or lack of consensus around these hypotheses would strengthen the argument.
- **Overgeneralization**: The statement “this observation is both puzzling and paradoxical” (line 64) could be more nuanced. Consider acknowledging that while the trend is observed, it may be influenced by underreporting or demographic factors.

**Methods**

**Comments**

**1. Structure and Flow**

- **Subsection Headings**: Consider using more descriptive subheading for the Luminex assay (e.g Cytokine Quantification via Luminex).
- **Redundancy**: Some procedural steps are repeated or overly detailed (e.g., incubation times, centrifuge models). These could be moved to supplementary materials to streamline the main text.

**2. Clarity and Precision**

- **Statistical Rationale**: While the statistical models are appropriate, the rationale for choosing specific models (e.g., linear mixed effects) could be briefly explained. Also, clarify why certain variables (e.g., TNF-β) were excluded.
- **Missing Data**: The explanation of missing data is good, but it would be helpful to include a brief statement on how missingness might bias results or how sensitivity analyses were handled (if any).

**3. Scientific Rigor**

- **Sample Size Justification**: While the total number of samples is provided, a brief justification of sample size adequacy (e.g., power calculation or expected effect size) would strengthen the methodological rigor.
- **Prioritization of Peptide Pools**: The decision to prioritize S1, S2, NP, and M pools when PBMCs were limited is reasonable, but it would be helpful to explain why these were chosen over others.

**Results**

**Comments**

**1. Clarity and Conciseness**

- **Overly Dense Paragraphs**: Several paragraphs (e.g., lines 286–305, 366–379) are data-heavy and difficult to parse. Consider breaking them into smaller, thematically grouped paragraphs to highlight key comparisons.
- **Redundant Phrasing**: Phrases like “significantly higher levels were observed…” are repeated frequently. Varying the language (e.g., “elevated,” “increased,” “amplified”) will help.

**2. Interpretation and Emphasis**

- **Highlight Key Findings Early**: The most striking results such as the association of higher IFN-γ responses with asymptomatic cases and elevated cytokines with severe disease should be emphasized earlier in each subsection.
- **Contextualize Findings**: For example, the observation that asymptomatic Kilifi patients had higher IFN-γ responses than those in Nairobi (lines 332–336) is important. Consider briefly suggesting why this might be the case (e.g., environmental exposures, prior infections) even in the Results section.
- **Clarify Sample Sizes**: When reporting subgroup analyses (e.g., IFN-γ responses by location), clearly state the number of participants in each group to help assess robustness.

**3. Figures and Tables**

- **Figure Captions**: Ensure that figure captions are descriptive and summarize the key takeaway. For example, Figure 1 could include a note like: “Higher IFN-γ responses to spike protein were observed in asymptomatic patients at diagnosis.”
- **Table 1**: This is informative, but is it possible to add a column for p-values to highlight significant demographic or clinical differences?

**Discussion and Conclusion**

**1. Clarity and Conciseness**

- **Overly Long Sentences**: Several sentences are dense and difficult to follow. For example:

“Thus, despite differences in levels of SARS-CoV-2 exposure, the immune systems of urban and rural residents may have had been pre-conditioned to respond differently…”

This could be simplified to:

“These findings suggest that prior environmental exposures may have conditioned immune responses differently in urban and rural populations.”

- **Repetitive Phrasing**: The phrase “higher levels of cytokines and chemokines” is repeated frequently. Consider varying your language (e.g., “elevated inflammatory markers,” “enhanced cytokine expression”).
- **Typographical Errors**:
  - Line 437: “the IFN-γ cellular response contribute” → should be “contributes.”

**2. Interpretation and Caution**

- **Causal Language**: Be cautious not to overstate causality. For example:

“SDF-1α may aid in the timely recruitment of T and other cells…”

This is speculative and should be framed as a hypothesis, e.g.:

“SDF-1α may play a role in recruiting immune cells to infection sites, potentially contributing to viral clearance.”

- **Balance in Urban-Rural Interpretation**: While the urban-rural immune response differences are compelling, the discussion could benefit from a more balanced tone. For instance, acknowledge that differences in healthcare access, reporting, or viral strain circulation could also contribute.

**3. Structure and Emphasis**

- **Highlight Key Findings Early**: The most important findings such as the association of early IFN-γ responses with asymptomatic infection and the distinct cytokine profiles by geography should be emphasized more clearly at the beginning of the discussion.
- **Conclusion Paragraph**: The final paragraph is strong but could be more concise.
