# Supplementary material for: Induction of an early IFN-γ cellular response and high plasma levels of SDF-1α are inversely associated with COVID-19 severity and residence in rural areas in Kenyan patients
Source: PLoS One. 2025 Sep 11;20(9):e0316967. doi: 10.1371/journal.pone.0316967 (PMC12425234; doi:10.1371/journal.pone.0316967)
Supplement: Attachment — Submitted filename: PWanjiku_P12082025_ Response to Reviewer2.docx [file pone.0316967.s016.docx]

12^th^ August 2025

The Editors

PLOS ONE-
1875 Mission Street
Suite 103 #188 San Francisco, CA 94103
United States.

Dear Editors,

Ref: Revisions in response to reviewer #2 for manuscript entitled “**Induction of an early IFN-γ cellular response and high plasma levels of SDF-1α are inversely associated with COVID-19 severity and residence in rural areas in Kenyan patients.**”

We thank Reviewer #2 for their thoughtful and constructive comments, which have now helped to greatly improve the manuscript. We have carefully considered all the suggestions and revised the manuscript accordingly.

Below, we provide detailed responses outlining how each comment was addressed.

All the reviewers’ comments and requests are in *italics*, and our responses are in regular text. The actual changes in the manuscript are in **bold** text, and we have indicated the page and line numbers.

*Reviewer #2:*

***Introduction***

1. *Structure and Flow*

*Redundancy: The final sentence of the introduction (lines 107–111) is repeated almost verbatim. This should be consolidated into a single, clear statement of the study’s objective.*

Response: Thank you for highlighting this redundancy. We have now revised the final sentence to consolidate the study objective into a single, clear statement.

Page 6, line 128 - 130. **<** **We then investigated relationships between the magnitudes of cellular IFN-γ, and several plasma cytokine and chemokine response profiles, with well characterized clinical disease phenotypes and geographic locations of the respective patients**.**>**

*Paragraph Transitions: The flow between paragraphs could be improved. For example, the shift from global hypotheses (lines 65–71) to cytokine storm mechanisms (lines 72–83) is abrupt. Consider adding a bridging sentence.*

Response: We have added a bridging sentence to ease the transition from global hypothesis to cytokine storm mechanisms to improve paragraph flow and strengthen the connection between these two concepts.

Page 4, line 79-81. **<** **However, the underlying immunological mechanisms remain understudied.>**

Additionally, we have added additional paragraph linkers:

Page 5, line 94 – 99. < **Thus, potential population differences in the context of cytokine and chemokine regulation could explain the differential burden in severe disease. Alternatively, there could have been a higher prevalence of pre-existing cross-protective antibody and T cell responses in SSA during the COVID-19 pandemic, owing to prior priming of the immune systems by increased exposure to pathogens with shared antigenic determinants with SARS-CoV-2.** >

2. *Clarity and Precision*

*Terminology: The phrase “plethora of inflammatory host responses” (line 72) is vague. Consider specifying “a broad range of pro-inflammatory cytokines and chemokines.”*

Response: We thank the reviewer for the suggestion. We have now replaced the phrase “plethora of inflammatory host responses” with “a broad range of pro-inflammatory cytokines and chemokines” to provide greater clarity and scientific precision.

Page 4, line 83 - 85. **<** **Like other viruses, SARS-CoV-2 induces a broad range of pro-inflammatory cytokines and chemokines (16), that play key roles in either conferring protective immunity, or exacerbating immunopathology (17).>**

*Ambiguity in Causality: The sentence “Thus, there is no evidence of the primary acute antibody response controlling the infection outcome” (lines 85–86) is too strong. Consider softening to “suggesting that antibody responses alone may not determine infection outcome.”*

Response: Thank you for this valuable suggestion. We have revised the sentence to reflect a more cautious interpretation.

Page 5, line 103-105. **<** **Thus, there is no evidence that the primary acute antibody response to SARS-CoV-2 in unvaccinated patients is sufficient for immune protection, without the contribution of other molecular and cellular players.>**

*Line 94: The sentence structure is awkward: “…as well as anti-inflammatory cytokines including IL-6 and IL-10, have been reported.” Consider rephrasing for clarity.*

Response: Thank you for this helpful observation. We have revised the sentence for clarity and grammatical accuracy.

Page 5, line 111-113. **<** **Associations between severe COVID-19 and elevated levels of pro-inflammatory cytokines like IFN-γ, TNF-α, and IL-17 and anti-inflammatory cytokines including IL-6 and IL-10, have been reported** (31)**.**

*3. Scientific Rigor*

*Balance in Hypotheses: While the introduction lists many plausible explanations for the SSA paradox, it does not critically evaluate them. A brief comment on the limitations or lack of consensus around these hypotheses would strengthen the argument.*

Response: We thank the reviewer for the suggestion. We have now added a new reference suggesting that the non-bological explanations are unlikely to fully explain the discordance in the rates of severe disease and deaths between sub-saharan Africa and Europe/North America – based on the widespread SARS-CoV-2 exposures and rates of asymptomatic infections reported in countries like Kenya and others in sub-Saharan Africa. We include two references in support.

Page 4, line 75-78. **<** **However, the widespread exposure to SARS-CoV-2 and high rates of asymptomatic infection observed in SSA countries suggest that non-biological explanations are unlikely to fully account for the reduced rates of severe disease and mortality in the continent, relative to wealthier regions like Europe and North America** (2,15)**.>**

*Overgeneralization: The statement “this observation is both puzzling and paradoxical” (line 64) could be more nuanced. Consider acknowledging that while the trend is observed, it may be influenced by underreporting or demographic factors.*

Response: We thank the reviewer for sharing this concern. However, we observed high levels and widespread exposure to SARS-CoV-2 in sub Saharan Africa, which was mostly associated with asymptomatic infections. Thus whilst factors like undereporting could contribute to the observation, they are less likely to explain fully, why there the rate of severe disease was reduced (relative to Europe and North America). We have expanded the sentence to provide more context, and have referenced the articles that describe the discordance in COVID-19 morbidity and mortality rates between Africa and wealthier territories. Importantly, public health experts had predicted far more deaths in Africa (based on the earlier reports from Europe) because of the weaker and massively underfunded health systems. Because this prediction did not materialise, we were both surprised and puzzled.

Page 4, line 64-67. **<This observation is both puzzling (3) and paradoxical (4), because the opposite was predicted by public health experts, fearing that the weaker and underfunded health systems in SSA countries would collapse when presented with similar rates of severe disease as initially reported in Europe.>**

***Methods***

*1. Structure and Flow*

*Subsection Headings: Consider using more descriptive subheading for the Luminex assay (e.g Cytokine Quantification via Luminex).*

Response: We have revised the subheading as advised.

Page 11, line 236. **<** **Quantification of cytokines by luminex assay.>**

*Redundancy: Some procedural steps are repeated or overly detailed (e.g., incubation times, centrifuge models). These could be moved to supplementary materials to streamline the main text.*

Response: We have now reduced the redundancies by replacing the name “Eppendorf Thermomixer Comfort Shaker” with <**shaker**>, and collapsing the phrase “at room temperature” to <**all incubations were done at room tempterature**>.

See page 11, line 249 - 255.

*2. Clarity and Precision*

*Statistical Rationale: While the statistical models are appropriate, the rationale for choosing specific models (e.g., linear mixed effects) could be briefly explained. Also, clarify why certain variables (e.g., TNF-β) were excluded.*

Response: Linear mixed-effect models were used as they are more appropriate for data with hierarchical (also called multilevel or clustered) structure, such as our data, which implies correlations among observations. Ignoring the hierarchical data structure, and therefore correlations among the observations can lead to misleading statistical inferences. We have now provided the explanations below and in the main text, and we have directed readers interested in the topic to a relevant text:

Page 12, line 276-278. **<** **LMERs were used exploit the hierarchical or clustered nature of our data. >**

Page 13, line 284 – 285. <**TNF-β data were excluded from the analyses as only one patient had a measurable concentration**.>

*Missing Data: The explanation of missing data is good, but it would be helpful to include a brief statement on how missingness might bias results or how sensitivity analyses were handled (if any).*

Response:We agree with the reviewer on the possibility of bias in some of the comparisons due to missing data. However, after an extensive exploration of the missing data, we found reasonably low levels of missing data in the outcomes which were also indifferentially distributed across the participant’s baseline characteristics, none of which had missing data values. Coupled with likelihood approaches, the bias that would be introduced would be little and the analyses still remain valid under the missing at random mechanism. We have presented details on missing data handling in the supplementary material. We have also acknowledged the plausibility of bias introduced by adding the sentence below in the discussion of study limitations..

Page 27, line 599-601. **<** **These gaps resulted in missing data for certain timepoints or peptide stimulations, which may have reduced the statistical power or even biased some comparisons. >**

*3. Scientific Rigor*

*Sample Size Justification: While the total number of samples is provided, a brief justification of sample size adequacy (e.g., power calculation or expected effect size) would strengthen the methodological rigor.*

Response: We thank the reviewer for this helpful comment. This study was initiated and conducted in response the COVID-19 pandemic, and aimed to recruit 200 participants (a pragmatic sample size with no formal sample size calculations) in each of two sites (Nairobi and Kilifi). However, this target was not achieved due to a number of challenges including limitations in the number of participants to include/recruit in diffuclt circumstances, and some partcipants were not always unavailable for follow ups. Furthermore, some of the individual participants’ samples lacked sufficient numbers and volumes of PBMC and plasma for conducting immunology assays. We have discussed this as a study limitation (**see discussion, page 27, lines 596 – 607**).

*Prioritization of Peptide Pools: The decision to prioritize S1, S2, NP, and M pools when PBMCs were limited is reasonable, but it would be helpful to explain why these were chosen over others.*

Response: Thank you for your comment. We initially screened 22 peptide pools by testing their capacity to induce IFN-γ production in PBMC from a small group of COVID-19 patients, and then ranked them according the magnsitudes of their specific responses. When faced with samples with limited numbers of PBMCs, we prioritized S2, S1, NP, and M, as they ranked among the top four. This clarification has been added to the Methods section.

Page 9, line 194-199. **<** **Because of limitations of PBMC numbers in individual samples, we prioritized performing the IFN-γ ELISpot assay against the top 4 of 22 peptide pools, based on the magnitude of the responses they induced in a pre-screening optimization experiment. The 10 pools that elicited the highest IFN-γ responses are S2, S1, NP, M, ORF3, NSP 12B, NSP 3C, NSP 15+16, NSP 3B and ORF8.>**

Page 10, line 226-230. **<** **For the samples that did not have enough PBMC to be tested against all the peptide pools, we prioritised measurements against pools from S2, S1, NP and M proteins, as they induced the highest IFN-γ responses in our pilot screening experiment.>**

***Results***

*Comments*

*1. Clarity and Conciseness*

*Overly Dense Paragraphs: Several paragraphs (e.g., lines 286–305, 366–379) are data-heavy and difficult to parse. Consider breaking them into smaller, thematically grouped paragraphs to highlight key comparisons.*

Response: We agree with the reviewer, and have now broken the two result sections into smaller paragraphs (see page 16, lines 323 - 345 and page 20, line 422 - 435).

*Redundant Phrasing: Phrases like “significantly higher levels were observed…” are repeated frequently. Varying the language (e.g., “elevated,” “increased,” “amplified”) will help.*

Response: We agree with the reviewer – and we have revised the text to vary the language in the results section as suggested.

Pages 16 to 18 (lines 323 – 391; pages 19 to 21 (lines 402 to 451).

*2. Interpretation and Emphasis*

*Highlight Key Findings Early: The most striking results such as the association of higher IFN-γ responses with asymptomatic cases and elevated cytokines with severe disease should be emphasized earlier in each subsection.*

Response: We are grateful to the reviwer for the suggestion to improve the presentation of reults. We have revised the section titles to state the most important results upfront.

Page 16, line 323 - 325. **<** **Increased frequencies of *ex vivo* spike specific IFN-γ secreting cells at the time of COVID-19 diagnosis are associated with asymptomatic SARS-CoV-2 infection and rural residency >**

Page 19, lines 402 – 403 <**High plasma levels for SDF-1α are inversely associated with COVID-19 severity and rural residence>**

*Contextualize Findings: For example, the observation that asymptomatic Kilifi patients had higher IFN-γ responses than those in Nairobi (lines 332–336) is important. Consider briefly suggesting why this might be the case (e.g., environmental exposures, prior infections) even in the Results section.*

Response: We aimed to limit discussion in the results section but we agree with the reviewer that contexulaising the key results will improve the manuscript. We have revised accordingly to include summaries that places the key results into some appropriated context, based on what is known.

Furthermore, we have revised the section heads and the figure legends so that they now act as result summaries.

Section heads:

Page 16, lines 323 – 325. < **Increased frequencies of *ex vivo* spike specific IFN-γ secreting cells at the time of COVID-19 diagnosis are associated with asymptomatic SARS-CoV-2 infection and rural residency>**

**Page 19, lines 402 – 403. <High plasma levels for SDF-1α are inversely associated with COVID-19 severity and rural residence>**

**Page 21, lines 460 – 461. < Principal component analysis: clustering cytokine and chemokine responses by disease severity group>**

Figure legends: please see section below dealing with **Figures and Tables**.

Result summaries:

Page 18, line 386 - 391. **<** **In summary, asymptomatic patients had higher anti-SARS-CoV-2 spike IFN-γ secreting cells than the symptomatic clinical phenotypes at the time of COVID-19 diagnosis, suggesting a possible protective role. Additionally, the asymptomatic individuals from rural areas had higher frequencies of anti-SARS-CoV-2 spike and NP peptides at the time of diagnosis than their counterparts in Nairobi city, suggesting modulation by either environmental exposures, or pre-existing cross-reactive protection.>**

Page 21, line 447 – 451. **< Collectively, the analyses above is consistent with COVID-19 immunology literature showing that high levels of pro-inflammatory cytokines are associated with severe disease. However, the association of high levels of inflammatory cytokines and chemokines with residency in Nairobi compared to Kilifi, and the vice versa for SDF-1α, would suggest modulation of the immune response by environmental exposures.>**

*Clarify Sample Sizes: When reporting subgroup analyses (e.g., IFN-γ responses by location), clearly state the number of participants in each group to help assess robustness.*

Response: We agree with the reviewer that this would improve the result presentation. We have now included the numbers of individuals for the various sub-analyses in the figures (below the x axes). This is preferable to us as the text is already heavy with numbers.

***3. Figures and Tables***

*Figure Captions: Ensure that figure captions are descriptive and summarize the key takeaway. For example, Figure 1 could include a note like: “Higher IFN-γ responses to spike protein were observed in asymptomatic patients at diagnosis.”*

Response: We agree this would improve the manuscript. We have revised the figure legend titles to summarise the data in the respective figures.

**Page 17, line 366-367: <Figure 1. Higher IFN-γ responses to spike protein were observed in asymptomatic infection at the day diagnosis.>**

Page 19, lines 392 – 393. **<Figure 2. Higher IFN-γ responses to spike protein were observed in asymptomatic infections from Kilifi than Nairobi at the day diagnosis.>**

Page 20, line 415-416. **<** **Figure 3. High plasma levels of pro-inflammatory cytokines and SDF-1α are positively and inversely, respectively, associated with COVID-19 severity. >**

Page 22, line 474-475. **<** **Figure 5. Asymptomatic patients cluster together in scatter plots by Severity at day of diagnosis. >**

*Table 1: This is informative, but is it possible to add a column for p-values to highlight significant demographic or clinical differences?*

Response: We thank the reviewer for this suggestion. We have included a column with the p values for the respective comparisons where applicable.

Page 14 and 15, **<** **Table 1 >**

***Discussion and Conclusion***

*1. Clarity and Conciseness*

*Overly Long Sentences: Several sentences are dense and difficult to follow. For example:*

*“Thus, despite differences in levels of SARS-CoV-2 exposure, the immune systems of urban and rural residents may have had been pre-conditioned to respond differently…”*

*This could be simplified to:*

*“These findings suggest that prior environmental exposures may have conditioned immune responses differently in urban and rural populations.”*

Response: We agree with the reviewer that that sentence was cumbersome and have now rewritten it to make it easier to follow. Additionally, we have revised several other sentences in the discussion for the same purpose.

Page 26, line 588-591. **<** **Consequently, the immune systems of individuals residing in urban and rural environments may have been predisposed to exhibit divergent responses to SARS-CoV-2, with inhabitants of urban regions demonstrating a higher propensity to generate elevated concentrations of inflammatory cytokines, which are correlated with the severity of COVID-19.>**

See also: page 23, line 504 – 507; page 24, lines 533 – 536; page 25, lines 554 – 558; page 26 lines 570 – 573; and page 26, lines 565 – 567 for similar revisions.

*Repetitive Phrasing: The phrase “higher levels of cytokines and chemokines” is repeated frequently. Consider varying your language (e.g., “elevated inflammatory markers,” “enhanced cytokine expression”).*

Response: We have now revised the results and discussion to minimise the repetitions for “**higher levels**”.

*Typographical Errors:*

*Line 437: “the IFN-γ cellular response contribute” → should be “contributes.”*

Response: We thank the revieweing for noting this. we have coirrected the typo and scanned for others throught the document.

*2. Interpretation and Caution*

*Causal Language: Be cautious not to overstate causality. For example:*

*“SDF-1α may aid in the timely recruitment of T and other cells…”*

*This is speculative and should be framed as a hypothesis, e.g.:*

*“SDF-1α may play a role in recruiting immune cells to infection sites, potentially contributing to viral clearance.”*

Response: We agree.

Page 24, line 539-540. **<** **SDF-1α may play a role in recruiting immune cells to infection sites (52), potentially contributing to viral clearance>**

*Balance in Urban-Rural Interpretation: While the urban-rural immune response differences are compelling, the discussion could benefit from a more balanced tone. For instance, acknowledge that differences in healthcare access, reporting, or viral strain circulation could also contribute.*

Response: We agree, and have modified the language to include other possible explanations whilst toning down the language stating the compelling hypothesis.

Page 26, line 570 – 573. **<While this disparity may partly be explained by elevated rates of SARS-CoV-2 transmission in densely populated urban centers, or by more comprehensive case reporting (31,64,65) among other possibilities, we contemplated whether there existed credible biological causes.>**

*3. Structure and Emphasis*

*Highlight Key Findings Early: The most important findings such as the association of early IFN-γ responses with asymptomatic infection and the distinct cytokine profiles by geography should be emphasized more clearly at the beginning of the discussion.*

Response: We agree with this advice and have extended the first paragraph to state the key results upfront.

Page 23, line 496 - 503. **<** **Our investigation revealed that a heightened cellular-IFN-γ response to SARS-CoV-2 spike peptides at the time of diagnosis is associated with asymptomatic infection, in marked contrast to both mild and severe presentations of COVID-19. The converse was true for SDF-1α, whose plasma levels were inversely associated with disease severity at all time points. We additionally observed divergent immune profiles when contrasting COVID-19 patients from urban and rural settings, with individuals from rural areas exhibiting diminished plasma concentrations of inflammatory markers but heightened plasma SDF-1α and early-cellular IFN-γ responses.>**

*Conclusion Paragraph: The final paragraph is strong but could be more concise.*

Response: We have revised the conclusion paragraph for clarity.

Pages 27/28, from line 608-632. **<** **In conclusion, while the occurrence of severe disease was relatively infrequent in Kenya, the inflammatory cytokine profile observed in patients experiencing severe COVID-19 closely resembles that of individuals diagnosed with severe COVID-19 in North America and Europe. An elevated early secretion of IFN-γ by immune cells, along with heightened concentrations of the chemokine, SDF-1α, were correlated with asymptomatic infections, thereby implying potential protective functions of these immune responses in mitigating the advancement of the disease. Furthermore, the differential profiles of cytokines, chemokines, and IFN-γ secreting cells, observed between patients from Nairobi and those from Kilifi, provide a compelling biological explanation for the increased incidence of severe COVID-19 in SSA urban centers, in contrast to their rural counterparts. Collectively, these observations yield valuable understanding regarding the immune responses that may confer protection against COVID-19, as well as their modulation by varying environmental exposures in geographically distinct regions. Nonetheless, additional studies are required to extend and replicate these important findings as they could inform future control of COVID-19 and empower the control of similar pandemics.>**

We are grateful for the opportunity to address these concerns and trust that our revisions and responses will be met with favourable review. Please let me know if I can be of any further assistance.

Thank you for your continued consideration of the manuscript.

Yours sincerely,

Perpetual Wanjiku

KEMRI-Wellcome Trust Research Programme
